# Antimicrobial, anticancer activities and molecular docking of eco-friendly chitosan nanocapsule loaded with biosynthesized titanium nanoparticles by *Aspergillus flavus*

**DOI:** 10.3389/fmicb.2026.1741753

**Published:** 2026-02-11

**Authors:** Nashwa El-Gazzar, Marwa Rafat, Gamal Rabie, Basel Sitohy

**Affiliations:** 1 Department of Botany and Microbiology, Faculty of Science, Zagazig University, Zagazig, Egypt; 2 Department of Clinical Microbiology, Infection and Immunology, Umeå University, Umeå, Sweden; 3 Department of Diagnostics and Intervention, Oncology, Umeå University, Umeå, Sweden

**Keywords:** anticancer activity, antimicrobial activity, *Aspergillus flavus*, chitosan nanocapsules, encapsulation efficiency, molecular docking, titanium nanoparticles

## Abstract

**Introduction:**

Chitosan have been leveraged to create chitosan nanocapsules within a bio-based nanocarrier system, enhancing efficacy and overcoming widespread microbial resistance. To generate chitosan nanocapsules (CNCs) that act as inhibitory agents against pathogenic microbes, this study combined titanium nanoparticles (TiO_2_ NPs) with chitosan nanoparticles (Cs NPs).

**Methods:**

*A.flavus* was used for the biosynthesis of TiO_2_NPs. Dynamic light scattering (DLS), zeta potential, atomic force microscopy (AFM), scanning electron microscopy (SEM), transmission electron microscopy (TEM) and X-ray diffraction (XRD), were utilized to assess the physicochemical properties of TiO_2_NPs and their CNCs. These techniques clarified particle diameter, charge stability, specific surface area, surface morphology, shape, dimensional forms, and structural parameters, respectively.

**Results:**

The findings showed that TiO_2_NPs and their nanocapsules achieved an encapsulation efficacy of over 86.7 ± 1.8% at 1.5% w/v chitosan concentration, with particle sizes of 40.7, 40.6, and 87.3 nm for TiO_2_NPs, CsNPs, and CNCs, respectively. Nanoparticle stability was confirmed by a zeta potential greater than –30.1 ± 4.5mV for TiO_2_NPs. Furthermore, TiO_2_NPs and their nanocapsules suppressed both Gram-positive and Gram-negative bacteria, with CNCs exhibiting more potent inhibitory effects than either TiO_2_NPs or CsNPs. The minimum inhibitory concentrations (MICs) of CNCs against *Salmonella typhimurium* and *Aspergillus fumigatus* were remarkably low at 10 μg mL^−1^. TEM images of *S. typhimurium* and *A. fumigatus* treated with CNCs exhibited asymmetric cell deformations, wrinkled external surfaces, cell depressions, and declined cell counts. Cytotoxicity studies showed that CNCs exhibited non-cytotoxic behavior on normal human melanocytes (HFB4). In contrast, CNCs reduced the viability of human colon carcinoma (HCT-116) and hepatocellular carcinoma (HepG-2).

**Conclusion:**

Nanomaterials, both alone or in nanocapsules, offer a promising alternative for inhibiting harmful microorganisms and represent a potential pathway for the development of anticancer medications. The findings indicate that CNCs are safe and effective against multidrug-resistant bacteria and fungi, making them a viable alternative to current antibiotic therapies.

## 1 Introduction

Antimicrobial-resistant microorganisms have emerged because of the widespread usage of antimicrobials as therapeutic agents for various human ailments in underdeveloped nations (Enan et al., 2020). Either directly via the food chain or indirectly through the transfer of antimicrobial resistance genes to human pathogens via mobile genetic elements linked to conjugative plasmids, antibiotic-resistant microbes can infect humans and adversely affect the environment and human health, leading to acute poisoning, cancer, and liver diseases (Abdel-Shafi et al., 2020; Abou Elez et al., 2021). Therefore, developing innovative antimicrobial mechanisms that are environmentally friendly is an essential and unavoidable medical priority (Sitohy et al., 2021; Abou Elez et al., 2023; El-Gazzar et al., 2024). Since investigating novel approaches to combat multidrug-resistant bacteria and discovering new antibiotics is challenging, scholars are now focusing on microbial strains, such as using nanomaterials (El-Gazzar and Ismail, 2020; Sitohy et al., 2021) and nanocomposites (El-Gazzar et al., 2025).

Nanomaterials are highly efficient delivery systems because of their fast and efficient biological absorption compared to larger macromolecules (El-Bahr et al., 2021; El-Gazzar et al., 2021). TiO_2_NPs, or titanium dioxide nanoparticles, are among the most extensively assessed nanomaterials and have recently garnered substantial interest because of their antimicrobial activity against bacteria and fungi. By impairing cell structure and function, TiO_2_NPs cause fungal membrane disruption, ultimately leading to cell death (El-Gazzar and Ismail, 2020; Huang et al., 2023). TiO_2_NPs have diverse applications and hold potential in environmental science, electronics, medicine, and energy. Moreover, titanium’s favorable chemical and mechanical properties make it a suitable material for effectively targeting tumor cells in combination with chemotherapeutic agents (Haji et al., 2024).

Although natural TiO_2_NPs exhibit strong antimicrobial efficacy against pathogenic microbes and environmental stressors, their biodegradability, hybrid characteristics, and stability are limited. Consequently, nanocapsule formulations have showed promise in enhancing and prolonging the antimicrobial activity of natural TiO_2_NPs (El Asbahani et al., 2015; Jamil et al., 2016). Furthermore, the hybrid properties of bionanocapsules composed of natural polymers, particularly polysaccharides, and inorganic materials have attracted considerable interest. Chitosan-based nanocapsules have attracted extensive research attention over the past two decades because of their exceptional properties and potential for biomedical applications.

Chitosan has been demonstrated to be an affordable carrier for various pharmacological agents (Piras et al., 2015). It is a great carrier system because of its cationic charge, natural antimicrobial capacity, biodegradability, availability, safety, and biocompatibility. Additionally, chitosan-based nanocapsules can protect natural antimicrobial compounds, enhancing their antibacterial activity and utility. Furthermore, the most effective antibacterial and anticancer products are produced through the biosynthesis and functionalization of nanoparticles and nano-encapsules. Therefore, the current research investigated the cytotoxic, antibiotic, physicochemical, and microscopic characteristics of functionalized engineered chitosan nanocapsules (CNCs) and TiO_2_NPs.

## 2 Materials and methods

### 2.1 Microorganisms used as indicators

The microorganisms were obtained from the Regional Center for Mycology and Biotechnology (Al-Azhar University, Cairo, Egypt) and used as indicators. *Bacillus subtilis* RCMB 015 NRRL 543, *Streptococcus pyogenes* ATCC 19615, *Staphylococcus aureus* ATCC 25923, *Escherichia coli* ATCC 25922, *Salmonella typhimurium* ATCC 14028, *Listeria monocytogenes* LMG10470, *Klebsiella pneumoniae* ATCC13883, *Pseudomonas aeruginosa* LMG 8029, and *Enterobacter cloacae* ATCC 23355 were the bacterial isolates used. Subcultures of these bacteria were grown on Brain Heart Infusion (BHI) broth (Oxoid) at 37°C. They were then refrigerated for a month before being employed as BHI agar slope cultures (Osman et al., 2021). *Penicillium aurantiogriseum* IMI89372, *Candida albicans* RCMB 005003 ATCC 10231, and *Aspergillus fumigatus* RCMB 002008 were among the fungal strains used. They were kept at –20°C in glass beads and then prepared as slope cultures after being enriched with PDA broth at 30°C. Subsequently, the cultures were refrigerated for a month before being used.

### 2.2 Antibiotic susceptibility test of some pathogenic bacteria

The bacteria tested in this study included *Salmonella typhimurium*, *Streptococcus pyogenes*, *Pseudomonas aeruginosa*, *Listeria monocytogenes*, *Klebsiella pneumoniae*, *Staphylococcus aureus*, *Bacillus subtilis*, *Escherichia coli*, and *Enterobacter cloacae*. According to Enan et al. (2020), the stock bacterial cultures were regularly sub-inoculated in Brain Heart Infusion Broth (BHIB) (Oxoid) and maintained at 4°C. Based on the guidelines of the Clinical and Laboratory Standards Institute [CLSI] (2008), the standard disc diffusion approach was utilized to test the susceptibility of the aforementioned bacteria to eight antibiotics. The cultures were cultivated for 12 h in nutrient broth (Oxoid). Muller-Hinton agar (Hi-Media, Mumbai, India) was utilized to plate the inocula after exposure to 10^5^ CFU/mL. Clindamycin (DA: 2 μg), ampicillin (AMP: 10 μg), tetracycline (TE: 30 μg), gentamicin (GEN: 20 μg), ciprofloxacin (CIP: 30 μg), ofloxacin (OFX: 5 μg), amoxicillin (AMC: 30 μg), and penicillin G (P: 10 μg) were all used at the concentrations indicated in parentheses. These antimicrobials were added to Muller-Hinton agar plates inoculated with the test bacteria. The plates were then inverted and incubated for 24 h at 37°C (Osman et al., 2021). After being measured and compared to the global criteria of Clinical and Laboratory Standards Institute [CLSI] (2008), the inhibition zones were divided as sensitive (S), intermediate (I), or resistant (R). According to Magiorakos et al. (2012), isolates were considered multi drug resistance (MDR) if they were resistant to at least three distinct antimicrobial classes. To estimate the multiple antibiotic resistance (MAR) index for each isolate, the Krumperman protocol was followed (Krumperman, 1983). The formula was a/b, where “a” represents the number of antimicrobial agents to which an isolate exhibited resistance, and “b” denotes the total number of antimicrobial agents evaluated. Gu et al. (2003) and Enan et al. (2013) reported the indicator of doubled antibiotic impedance.

### 2.3 Antifungal susceptibility test

*Aspergillus fumigatus*, *Penicillium aurantiogriseum*, and *Candida albicans* were evaluated for antifungal susceptibility utilizing the Kirby-Bauer Disc Diffusion technique. The Clinical and Laboratory Standards Institute guidelines (17) were followed in determining and interpreting inhibition zones. Eight antifungal drugs were tested, including terbinafine (TER: 30 mg), amphotericin (AMB: 20 mg), nystatin (NYS: 100 I.U.), fluconazole (FLZ: 25 mg), flucytosine (FUS: 1 mg), itraconazole (ITR: 10 mg), micafungin (MFG: 1 mg), and caspofungin (CAS: 5 mg). The multiple antifungal resistance (MFR) index and multidrug resistance (MDR) were calculated (Sanglard, 2016; Cavalcanti Filho et al., 2017).

### 2.4 Preparations of TiO_2_NPs, CsNPs, and CNCs

#### 2.4.1 Biosynthesis TiO_2_NPs

In this work, *Aspergillus flavus* cell-free supernatant was employed as a biocatalyst to biosynthesize TiO_2_NPs from TiO_2_ (Nanotech Company, Dream Land, Egypt). The *A. flavus* cell-free filtrate was combined with 1 mM TiO_2_, following the approach described El-Gazzar and Ismail (2020). The reaction flask was then incubated for 3–5 days at 28°C, after which TiO_2_NPs were formed.

#### 2.4.2 Molecular identification of the fungal isolate utilized in the biosynthesis of TiO_2_NPs

The fungal isolate was obtained from soil contaminated by photographic industry waste (El-Sharkia Governorate, 80 km north of Cairo, Egypt) (El-Gazzar and Ismail, 2020). According to Abdel-Salam et al. (2001), it was sub-cultured in potato dextrose broth (Oxoid) and preserved in glass beads. After being sent to the Molecular Unit at Assiut University, the culture was maintained in the Molecular Culture Collection (AUMC) of the same institution. The DNA was stored in 1.5 mL autoclaved Eppendorf tubes before being delivered to SolGent Company in Daejeon, South Korea, for amplifying the fungal ITS region and polymerase chain reaction (PCR). For PCR, ITS1 (forward) and ITS4 (reverse) primers were added to the reaction mixture. The primer sequences were ITS1 (5’-TCC GTA GGT GAA CCT GCG G-3’) and ITS4 (5’-TCC TCC GCT TAT TGA TAT GC-3’). Following the addition of dNTPs to the PCR reaction mixture, the extracted PCR product was sequenced using the same primers (White et al., 1990). The gathered sequences were analyzed utilizing the Basic Local Alignment Search Tool (BLAST) on the National Center for Biotechnology Information (NCBI) website. Phylogenetic analysis of the sequences was performed using the MegAlign software (DNAStar) version 5.05 (El-Gazzar et al., 2025).

#### 2.4.3 Chitosan solution preparation

High molecular weight chitosan (degree of deacetylation ≥ 85%) was dissolved in 4% (v/v) acetic acid solution to prepare a 2% (w/v) chitosan stock solution (Elabd et al., 2023; Mahboub et al., 2025a). The dissolution process was performed under continuous magnetic stirring at 200 rpm for 24 h at room temperature to ensure full hydration and dissolution of the chitosan polymer chains. The pH of the chitosan solution was kept at 4.2 ± 0.1, representing the optimal condition for chitosan solubility while preserving its cationic properties essential for subsequent ionic crosslinking reactions. The solution was filtered via a 0.45 μm membrane filter to eliminate any undissolved particles and stored at 4°C till additional usage (Ibrahim et al., 2025).

#### 2.4.4 Nanocapsule formation via ionic gelation

The chitosan-TiO_2_ nanocapsules were synthesized using the ionic gelation technique with sodium tripolyphosphate (STPP) as the crosslinking agent (Al-Gethami et al., 2022). Initially, the synthesized TiO_2_ nanoparticles (100 mg) were dispersed in 50 mL of the prepared chitosan solution under ultrasonication for 30 min at 40 kHz to ensure uniform distribution and prevent particle aggregation. The mixture was then subjected to high-speed homogenization at 4°C at 15,000 rpm for 15 min to achieve optimal dispersion of TiO_2_ within the chitosan matrix. Subsequently, STPP solution (1% w/v), prepared in distilled water at pH 8.0 ± 0.2, was added dropwise to the chitosan-TiO_2_ suspension at a volumetric ratio of 3:1 (chitosan:STPP) under continuous stirring at 800 rpm (Al-Qasmi et al., 2022; Katowah and Ismail, 2025).

### 2.5 Loading efficiency and drug release methodology

#### 2.5.1 Loading efficiency determination

##### 2.5.1.1 Preparation of drug-loaded nanocapsules

The loading efficiency of chitosan-titanium dioxide nanocapsules was determined using a model drug incorporation method following established protocols (El-Sherbiny et al., 2024). Drug loading was performed using a modified ionic gelation method, in which ciprofloxacin solution was added to the chitosan–TiO_2_ dispersion prior to STPP crosslinking under controlled stirring. A standardized amount of ciprofloxacin hydrochloride compound (1,080 mg) was dissolved in 5 mL of acetic acid buffer, pH between 3.5 and 6.5) to create a stock solution with a concentration of 2 mg/mL. The drug solution was incorporated into the chitosan-TiO_2_ suspension during the ionic gelation process by adding it to the chitosan solution immediately before the addition of the STPP crosslinking agent. Drug incorporation was conducted under continuous magnetic stirring for 30 min at room temperature (25 ± 2°C) at 500 rpm to ensure uniform distribution throughout the polymer matrix (Abdel-Hamied et al., 2022).

##### 2.5.1.2 Separation and quantification protocol

Following the completion of nanocapsule formation and drug loading, the suspension was centrifuged at 15,000 rpm for 25 min at 4°C to separate the drug-loaded nanocapsules from the supernatant containing unencapsulated drug. The supernatant was carefully gathered and filtered through a 0.22 μm membrane filter to eliminate any residual nanocapsule particles. The concentration of free drug in the supernatant was identified using UV-visible spectrophotometry at the drug’s characteristic absorption wavelength (λ_*max*_ = 280 nm) with a calibrated standard curve prepared from known drug concentrations ranging from 1 to 100 μg/mL (Gad et al., 2022). All measurements were performed in triplicate using quartz cuvettes with a 1 cm path length, and absorbance readings were recorded against the corresponding buffer blank.

##### 2.5.1.3 Loading efficiency calculation

The currently accepted formulas were utilized to assess the encapsulation efficiency (EE%) and loading efficiency (LE%) (Elshayb et al., 2022):

Loading Efficiency (%) = (Weight of drug loaded in nanocapsules/Weight of nanocapsules) × 100

Encapsulation Efficiency (%) = (Initial drug amount − Free drug in supernatant/Initial drug amount) × 100

The weight of the drug loaded in nanocapsules was estimated by subtracting the amount of free drug detected in the supernatant from the initial amount of drug added to the formulation. The nanocapsules were dried using freeze-drying at –80°C for 48 h under vacuum conditions (0.01 mbar) to determine the total dry weight of the drug-loaded nanocapsules (Alshubramy et al., 2025).

#### 2.5.2 *In vitro* drug release studies

##### 2.5.2.1 Release medium preparation and experimental setup

Drug release studies were conducted using the dialysis bag method in different pH environments to simulate physiological conditions (Hassan et al., 2023). Phosphate-buffered saline (PBS, pH 7.4), representing physiological circumstances; simulated intestinal fluid (SIF, pH 6.8), mimicking the intestinal environment; and simulated gastric fluid (SGF, pH 1.2) prepared based on USP criteria, were used as release media., Acetate buffer (pH 5.5) was used to stimulate the acidic infection microenvironment Each release medium was freshly prepared and maintained at 37 ± 0.5°C throughout the experiment using a thermostatic water bath with continuous orbital shaking at 100 rpm, simulating physiological agitation conditions (Mahboub et al., 2025a).

##### 2.5.2.2 Dialysis and sampling protocol

With a molecular weight cut-off of 12–14 kDa, drug-loaded nanocapsules containing 5 mg of the encapsulated medication were suspended in 2 mL of PBS (pH 7.4) and placed into pre-soaked dialysis bags. To maintain sink conditions, with the drug concentration kept significantly below saturation solubility, the sealed dialysis bags were submerged in 200 mL of the corresponding release medium. To preserve these conditions, 3 mL aliquots were withdrawn from the release medium at predefined intervals (0.5, 1, 2, 4, 6, 8, 12, 24, 48, 72, and 96 h) and immediately replaced with a similar volume of fresh medium kept at the same temperature (Mahboub et al., 2025b).

##### 2.5.2.3 Analytical determination and data analysis

The concentration of released medication in each sample was quantified using validated UV-visible spectrophotometry at λ_*max*_ = 278 nm, with appropriate dilutions applied when necessary to maintain readings within the linear calibration range. The cumulative percentage of drug released was calculated at each time point using the formula (Ibrahim et al., 2025): -

Cumulative Release (%) = (Σ Amount of drug released at time t/Total amount of drug loaded) × 100

To assess the drug release process, release kinetics were analyzed by fitting the experimental data to several mathematical models, involving zero-order, first-order, Higuchi, Korsmeyer-Peppas, and Hixson-Crowell models. The correlation coefficient (*R*^2^) values were calculated for each model to identify the best-fitting kinetic model. Tukey’s *post-hoc* test and One-way ANOVA were applied for statistical analysis, with a *p* < 0.05 regarded as statistically significant (Al-Gethami et al., 2022).

##### 2.5.2.4 Stability and pH-responsive release assessment

The pH-responsive behavior of the nanocapsules was evaluated through sequential release studies, which began in SGF (pH 1.2) for 2 h, followed by a transfer to SIF (pH 6.8) for 6 h, and finally to PBS (pH 7.4) for the remaining duration. This protocol simulates gastrointestinal transit and assesses the potential for targeted drug delivery. Dynamic light scattering (DLS) was applied to identify changes in zeta potential and particle size distribution throughout the release study period, thereby evaluating the integrity of the nanocapsules under different pH conditions (Katowah and Ismail, 2025).

##### 2.5.2.5 UV-triggered release study

For UV- release studies, dialysis bags containing drug-loaded nanocapsules were exposed to UV light (365 nm, 15 mW/cm^2^) at predefined intervals while immersed in PBS (pH 7.4). Control samples were maintained under identical condition in the absence of UV irradiation (Ibrahim et al., 2025).

### 2.6 Characterization of TiO_2_NPs, CsNPs, and CNCs

The morphological and physicochemical characteristics of the produced TiO_2_NPs, CsNPs, and CNCs were determined using several analyses.

#### 2.6.1 Determination of zeta-size

The sizes of TiO_2_NPs, CNCs, and CsNPs were determined using zeta-size measurements (El-Gazzar et al., 2020).

#### 2.6.2 Determination of zeta potential

Zeta potential measurements were conducted using a Malvern Zetasizer Nano ZS instrument (Malvern Panalytical, United Kingdom) with factory-calibrated DTS1235 disposable folded capillary cells. Prior to measurement, nanoparticle suspensions underwent extended dialysis purification (72 h, MWCO 12–14 kDa) against ultrapure water (18.2 MΩ⋅cm resistivity, Milli-Q Direct system, Merck Millipore) with dialysate exchanges every 8 h to remove residual ionic species from crosslinking reactions. Final sample conductivity was verified to be < 5 μS/cm before analysis. Samples were diluted to optimal concentration (0.1–0.2 mg/mL) to ensure single scattering regime and minimize particle-particle interaction artifacts. Each sample was equilibrated at 25.0 ± 0.1°C for 15 min in the thermostated measurement cell before data acquisition. Zeta potential determination employed electrophoretic light scattering with automatic voltage optimization and consisted of 15 measurement runs with inter-run equilibration periods. Results represent the mean ± standard deviation of three independent measurements. Instrument performance was verified daily using certified zeta potential transfer standards (Malvern DTS1235, nominal ζ = −42 ± 4.2 mV, Acceptance criterion: measured value within 5% of nominal (El-Gazzar and Ismail, 2020).

To definitively validate colloidal stability regarding potential aggregation in suspension, comprehensive time-dependent monitoring was conducted for hydrodynamic diameter and zeta potential over a 30-day storage period at ambient conditions (25°C, dark storage to prevent photocatalytic effects). Measurements conducted at days 0, 7, 14, 21, and 30 following synthesis. Storage Conditions for Sealed polypropylene vials at 25 ± 1°C, protected from light, no stirring/agitation between measurements. Triplicate DLS and zeta potential measurements at each time point, 15-min thermal equilibration before analysis. One-way ANOVA with Tukey *post-hoc* test to assess temporal changes (*p* < 0.05 significance threshold).

#### 2.6.3 Transmission electron microscopy

In accordance with a previously described technique, TEM was used to determine the morphological properties of TiO_2_NPs, CsNPs, and CNCs (El-Gazzar et al., 2024).

#### 2.6.4 Scanning electron microscopy

Using SEM (JEOL, Akishima, Tokyo 196-8558, Japan), the surface morphology of TiO_2_NPs, CsNPs, and CNCs was further investigated (Sitohy et al., 2021).

#### 2.6.5 X-ray diffraction

Additionally, as described in a previous study, XRD was used to evaluate the structural properties of the produced TiO_2_ NPs, Cs NPs, and CNCs (El-Gazzar et al., 2024).

#### 2.6.6 Atomic force microscopy

The three-dimensional and two-dimensional AFM images of TiO_2_NPs, CsNPs, and CNCs were obtained using an AFM (5600LS, Agilent, Santa Clara, CA, United States). A thin film was prepared utilizing a spin-coating instrument (Laurell-650Sz) under vacuum conditions at 820 rpm. After the samples had been exposed to ultrasonic waves for 1 h at 60 kHz, 85% amplitude, and 0.6 cycles (UP400S, Hielscher, Teltow, Germany), the thin film was prepared (Sitohy et al., 2021).

### 2.7 Biological activities of TiO_2_NPs, CsNPs, and CNCs

The following is an analysis of the biological functions of each TiO_2_NP, CsNP, and CNC:

#### 2.7.1 Antimicrobial activity

#### 2.7.1.1 The antibacterial, antifungal activities and minimum inhibitory concentration of TiO_2_NPs, CsNPs, and CNCs

The antibacterial, antifungal activities and minimum inhibitory concentration (MIC) of TiO_2_NPs, CsNPs, and CNCs were assessed utilizing the agar well diffusion technique. Stock preparations of TiO_2_NPs, CsNPs, and CNCs (200 μg/mL) were suspended in methanol and stored at 5°C. As previously reported, antifungal and antibacterial activities were evaluated against the same strains (Enan et al., 2020; Abdel-Shafi et al., 2020).

The MIC values were determined by broth dilution procedures against *S. typhimurium* and *A. fumigatus* according to CLSI guidelines (CLSI M07, Clinical and Laboratory Standards Institute [CLSI] (2018); CLSI M38, Clinical and Laboratory Standards Institute [CLSI] (2017)), following EUCAST recommendations for standardized MIC reporting (European Committee on Antimicrobial Susceptibility Testing [EUCAST], 2023). TiO_2_NPs, CsNPs, and CNCs were separately prepared at various dilutions (5, 10, 20, 30, 40, 50, 70, and 100 μg/mL). Fresh microbial cultures (100 μL) were adjusted to a final inoculum concentration of about 5 × 10^5^ CFU/mL and then inoculated into tubes that treated by TiO_2_NPs, CsNPs, and CNCs dilutions. Growth controls (medium with inoculum). Following, incubation at 37°C for 18–24 h for *S. typhimurium* and 28–30°C for 48 h for *A. fumigatus.* Every dilution was evaluated in triplicate in a separate experiment. Microbial growth was then read visually. The lowest concentration of nanoparticles that inhibited visible microbial growth was recorded as the MIC values (μg/mL) (Abou Elez et al., 2023; El-Gazzar et al., 2024).

##### 2.7.1.2 TEM analysis

The most multidrug-resistant bacteria, *S. typhimurium*, and *A. fumigatus* were selected for TEM analysis to identify ultrastructural morphological alterations induced by TiO_2_NPs, CsNPs, and CNCs. Fresh cultures of *A. fumigatus* and *S. typhimurium* (10^6^ CFU/mL) were incubated at 37°C for 4 h after independent treatment with the appropriate MIC. Control cultures received no treatment and were maintained under the same conditions as the experimental cultures. To separate the cells of the test strains, cultures were centrifuged for 10 min at 4,000 rpm. The cells were thoroughly rinsed with distilled water, fixed, and then kept at room temperature for 5 min in a solution containing potassium permanganate and 3% glutaraldehyde. The samples were subsequently dehydrated in pure ethanol for 30 min, mounted on TEM copper grids, sectioned into thin slices, embedded in pure resin, and double-stained with lead citrate and uranyl acetate for TEM analysis (JEOL JEM-1010, Tokyo, Japan) (Abou Elez et al., 2023).

#### 2.7.2 Anticancer activity

Based on the methodology mentioned in prior studies, CsNPs, TiO_2_NPs, and CNCs were evaluated for their inhibitory effects, cell survival rate, and anticancer activity utilizing a colorimetric MTT assay at the VACSERA Tissue Culture Unit (Gomha et al., 2015; Mosmann, 1983). For the cytotoxicity test, trypsin treatment and washing were carried out on human normal melanocytes (HFB4), human colon carcinoma (HCT-116), and human hepatocellular carcinoma (HepG-2). The cells were then seeded in 96-well plates with RPMI-1640 medium supplemented with 10% fetal bovine serum (FBS) at a final density of 10^4^ cells/100 μL. The plates were incubated with 5% CO_2_ at 37°C for 1 day. The medium was subsequently replaced with FBS-free media containing various concentrations of the tested compounds at two-fold serial dilutions (3.90, 7.80, 15.60, 31.25, 62.50, 125, 250, and 500 μg/mL).

After incubation with 5% CO_2_ at 37°C for 2 days, the wells were rinsed with PBS. PBS and 50 μL of the MTT reagent (3-(4,5-dimethylthiazol-2-yl)-2,5-diphenyltetrazolium bromide) at 0.5 mg/mL were then added to the wells, and the plates were incubated for 4 h. Following removal of the supernatants, 50 μL of 99.9% dimethyl sulfoxide was added to each well to dissolve the precipitated dark blue formazan crystals, and the mixture was stirred. The supernatants were then discarded, and a microplate reader (SunRise, TECAN, Inc., United States) was utilized to estimate the formazan absorbance at 490 nm, thereby determining the number of viable cells. The concentration needed to decrease cell viability by 50% was measured utilizing GraphPad Prism software (San Diego, CA, United States) (Pandy et al., 2023). The percentage of viability was calculated as follows:

%Cell viability = Mean O.D of treated cells/Mean O.D of untreated cells × 100

### 2.8 Cytotoxicity evaluation and determination of oxidative enzymes

#### 2.8.1 Antioxidant assay using ABTS scavenging capacity

The ability of antioxidants to directly react with ABTS radicals generated by a chemical process was evaluated using the ABTS scavenging capacity assay (Re et al., 1999). As previously reported, the ABTS radical scavenging method was employed to evaluate antioxidant activity (Samaniego Sánchez et al., 2007; Ling et al., 2009). A 1.8 mM ABTS stock solution (Sigma, PN: A3219) was mixed with 0.63 mM potassium persulfate to form the ABTS radical cation (ABTS^+^), which was then maintained in the dark for 12–16 h at room temperature before usage. Ethanol was added until the absorbance at 734 nm reached 0.700 (± 0.030). Samples or extracts were diluted 1:10 with 80% methanol at room temperature. In a microtiter plate, 10 μL of diluted extracts were combined with 190 μL of the radical solution. Absorbance at 734 nm was estimated every minute for up to 13 min after the initial mixing. For each test, solvent blanks were included as appropriate. Methanol was utilized as the negative control, and ascorbic acid served as the standard antioxidant. Every sample was evaluated in triplicate across three experiments.

The percentage of free radical scavenging activity was measured using the following formula:

% Free radical scavenging activity = [(A_*n*_–A_*s*_) × 100]/An

Where, A_*s*_ represents the final absorbance value of the sample and A_*n*_ represents the final absorbance value of the negative control.

#### 2.8.2 CDK-2 enzyme inhibition assay

For the enzyme inhibition assay, we used the cyclin-dependent kinase 2 (CDK2) assay kit (Catalog #79599, BPS Bioscience, San Diego, CA) (Eldehna et al., 2021).

#### 2.8.3 Propagation of carcinoma cell lines

The RPMI 1640 medium was used to cultivate the cell lines. Using a humidified water-jacketed CO_2_ incubator (Shel Lab, Sheldon Manufacturing, Inc.^®^, United States), the cancer cell lines were cultured at 37°C for 2 days in a 75 cm^3^ Corning^®^ culture flask. The cell monolayer was tested with an inverted microscope (CKX41; Olympus, Japan) to ensure the absence of bacterial or fungal contamination. The monolayer was rinsed with 5 mL of Ca^2+^/Mg^2+^-free phosphate-buffered saline (PBS), after which 2.5 mL of a 0.53 mM trypsin/EDTA solution was added to the culture flask and incubated for 7–15 min to facilitate cell detachment. To inactivate trypsin, 6 mL of maintenance medium was added after the cells were eliminated from the flask. Trypan blue staining was applied in a hemocytometer to count the number of viable cells (Wilson, 2000).

#### 2.8.4 Quantification of oxidative stress

The most affected cell line (HCT-116) was selected for more testing to evaluate the impact of oxidative stress on cancer cells. HCT-116 cells were cultured in 24-well tissue culture plates at a density of 5 × 10^5^ cells/mL. After the formation of a monolayer cell sheet in each well, the tested material was added at a concentration of 23.8 μg/mL with designated wells for cell controls and determination of 50% inhibitory concentration (IC_50_) values. Each treatment was performed in triplicate. After treatment, the growth medium was removed, and the cells were harvested from each well.

#### 2.8.5 Preparation of the cell lysate

Trypsin was utilized to extract the cells after the incubation period. After the addition of 2 mL of culture medium, the mixture was centrifuged in a Sigma refrigerated centrifuge for 10 min at 1,500 rpm at 4°C. The pellet was then washed three times with PBS (pH 7.4). It was subsequently resuspended in an extraction solution consisting of 20 mM potassium phosphate buffer (pH 7) and a cocktail of protease inhibitors. After 10 min of sonication in ice-cold normal saline (1/9, w/v) using a Virsonic^®^ ultrasonic cell disruptor, the cells were centrifuged at 5,000 rpm for 5 min at 4°C. The resulting supernatant was stored at –80°C till the study was completed (Timur et al., 2005; Alarifi, 2011).

#### 2.8.6 Estimation of protein

Utilizing bovine serum albumin as a reference standard, protein concentrations were determined in each sample according to the Bradford method Bradford (1976). Each well of a 96-well microplate was filled with 100 μL of protein sample or standard (in duplicate). Bradford reagent was then added, thoroughly combined, and allowed to stand for 5 min. The absorbance at 595 nm was measured after the incubation period. Protein content was determined on the same 96-well microplate by regression analysis of a series of standards (bovine serum albumin, 0–100 μg/mL).

#### 2.8.7 Glutathione (GSH) content

The intracellular GSH content was determined using the technique of Siddiqui et al. (2010), with minor modifications. Briefly, to fully precipitate proteins, 1 mL of sonicated cell suspension was mixed with 1 mL of 10% trichloroacetic acid (TCA) and kept on ice for 1 h. The combination was then centrifuged for 10 min at 3,000 rpm. 2 mL of 0.4 M Tris buffer (pH 8.9), 0.02 M ethylenediaminetetraacetic acid (EDTA), and 0.01 M 5,5’-dithionitrobenzoic acid (DTNB) were added, and the supernatant was diluted with 0.5 mL of distilled water to obtain a final volume of 3 mL. The tubes were kept in a 37°C water bath for 10 min with shaking. The absorbance of the resulting yellow color was estimated at 412 nm utilizing a microplate reader (SunRise, TECAN, Inc., United States).

#### 2.8.8 Activity of catalase

Hydrogen peroxide (H_2_O_2_) was utilized as a substrate to assess catalase activity, following the approach of Sinha (1972). The reaction combined with a final volume of 1 mL, contained phosphate buffer (pH 7.0), enzyme protein, and 0.08 μmol of H_2_O_2_. Enzyme activity was measured utilizing a UV-Visible spectrophotometer (Spectronic, Milton Roy, United Kingdom) by monitoring the disappearance of H_2_O_2_ at 570 nm.

#### 2.8.9 Superoxide dismutase activity

Using the technique illustrated by Kakkar et al. (1984), SOD activity was determined by preparing a 3 mL reaction mixture containing 186 μM phenazine methosulfate (PMS), 0.052 M sodium pyrophosphate buffer (pH 8.3), 780 μM NADH, sonicated enzyme preparation, 300 μM nitroblue tetrazolium (NBT), and water. The reaction was then initiated and maintained for 90 s. Subsequently, 4.0 mL of n-butanol was added, and the combination was shaken vigorously. After standing for 10 min, centrifugation was performed to separate the butanol layer. The color intensity of the chromogen in butanol was estimated at 560 nm using a spectrophotometer. An enzyme-free mixture with cell suspension served as the control.

#### 2.8.10 Malondialdehyde assay

This approach is used to evaluate malondialdehyde (MDA), the final indicator of the lipid peroxidation pathway. Depending on Buege and Aust (1978), the principle of this assay is based on the reaction between MDA and thiobarbituric acid (TBA), forming the MDA-TBA adduct, which can be quantified colorimetrically. Briefly, cells were gathered by centrifugation, sonicated in 1.15% ice-cold potassium chloride, and centrifuged at 3,000 rpm for 10 min. The resulting supernatant (1 mL) was mixed with 2 mL of TBA reagent (15% TCA, 0.7% TBA, and 0.25 N HCl) and heated for 15 min at 100°C in a boiling water bath. The mixture was then cooled, centrifuged for 10 min at 1,500 rpm, and the absorbance of the supernatant was estimated at 535 nm.

### 2.9 Evaluation of the mechanism of cytotoxicity against the HCT-116 cell line with ROS determination

The human colon cancer cell line (HCT-116) was gathered from the American Type Culture Collection (ATCC, Rockville, MD). Cells were cultured in Dulbecco’s modified Eagle’s medium (DMEM) supplemented with 10% heat-inactivated fetal bovine serum, 1% L-glutamine, 50 μg/mL gentamycin, and HEPES buffer. Cultures were kept at 37°C in a humidified atmosphere containing 5% CO_2_. To evaluate the potential mode of action on the cell line, HCT-116 cells were seeded into 6-well plates the day before the experiment at a density of 5 × 10^5^ cells/mL. Once a complete monolayer formed in each well, the active compounds were added at their IC_50_ concentration, with each treatment performed in triplicate. After incubation, cells were gathered utilizing 0.25% trypsin, and 2 mL of culture medium was added. The mixture was centrifuged at 1,500 rpm for 10 min at 4°C in a Sigma chilled centrifuge, followed by three washes with PBS (pH 7.4). The resulting pellet was resuspended in an extraction solution containing a 20 mM potassium phosphate buffer (pH 7) and protease inhibitor cocktail. Cells were sonicated on ice for 10 min in ice-cold normal saline (1/9, w/v) using a Virsonic^®^ ultrasonic cell disruptor, then centrifuged at 5,000 rpm for 5 min at 4°C. The supernatant was subsequently stored at –80°C until further analysis. Furthermore, utilizing prior-developed ELISA colorimetric kits based on the manufacturer’s instructions, the levels of reactive oxygen species (ROS) in HEPG-2 cells, whether treated or untreated, were assessed (Eldehna et al., 2018).

### 2.10 *In silico* study of CNCs

#### 2.10.1 Protein structure preparation

The three-dimensional crystal structures of the target proteins were collected from the Protein Data Bank (PDB). The selected targets included CYP51B from the pathogenic filamentous fungus *Aspergillus fumigatus* (PDB ID: 5FRB), FabG-D from *Salmonella typhimurium* (PDB ID: 6T7M), and cyclin-dependent kinase 2 (CDK2) from human colon carcinoma (PDB ID: 2FVD). All protein structures were prepared using UCSF Chimera software, in which co-crystallized ligands and water molecules were systematically removed. Polar hydrogen atoms were then added, and partial charges were allocated to optimize the structures for molecular docking calculations.

#### 2.10.2 Ligand preparation

The three-dimensional structures of chitosan-titanium nanoparticle complexes (Ch-Ti) were generated utilizing ChemBio Office software in combination with the PubChem database. The ligand corresponded to PubChem ID 57369765, with the molecular formula C_12_H_26_N_2_O_9_Ti and the SMILES notation C([C@@H]1C@HO)O)Ti. Ligand structures were optimized and prepared for docking simulations.

#### 2.10.3 Molecular docking protocol

Molecular docking simulations were performed utilizing AutoDock Vina software to assess the binding interactions between chitosan-titanium complexes and the target proteins. The docking protocol generated multiple binding poses for each ligand-protein pair, with the system configured to explore 12 active torsions, allowing for conformational flexibility. Binding energies were calculated at 298.15 K to assess the thermodynamic favorability of the interactions. The docking results included detailed energy calculations, encompassing intermolecular interactions (INTER), intramolecular interactions (INTRA), and energies of unbound ligands (UNBOUND).

#### 2.10.4 Analysis and evaluation

The generated binding poses were analyzed to identify key molecular interactions between the chitosan-titanium ligands and amino acid residues within the active sites of the target proteins. Hydrogen bonding patterns were characterized by measuring donor-acceptor distances and bond lengths. The binding affinity of each ligand for the respective target proteins was evaluated based on the calculated free binding energies (VINA RESULTS scores), with more negative values indicating stronger interactions. Additionally, detailed interaction maps were generated to visualize the specific amino acid residues included in ligand binding and the spatial orientation of the complexes within the protein binding sites.

### 2.11 Statistical analysis

All experiments were conducted in triplicate. Standard deviations (± SD) were calculated utilizing a one-way ANOVA (Duncan, 1955). Data were analyzed utilizing the statistical WASP software program 2.0, employing the least significant difference (LSD) technique at a significance level of *p* < 0.05. Significant differences are suggested by specimen signs (a.b), whereas non-significant differences are indicated by specimen signs (a.a) (El-Gazzar et al., 2020). Mean entrapment efficiency (MEE) was calculated as MEE = ΣEE%/n, standard error of the mean (SEM) as SEM = SD/√n, and 95% confidence intervals (CI) as CI = MEE ± (t × SEM), where t is the *t*-value corresponding to the desired confidence level.

## 3 Results

### 3.1 Antibacterial susceptibility of bacterial strains

As illustrated in the Materials and Methods portion, the trial was extended to evaluate the antibiotic sensitivity of additional pathogenic bacteria, with the results presented in Supplementary Table 1. The MAR index of the tested indicator bacteria was as follows: 75% for *S. typhimurium*; 62.5% for *Pseudomonas aeruginosa* LMG 8029, *S. aureus*, and *Listeria monocytogenes*; 50% for *K. pneumoniae* ATCC 13883; 37.5% for *E. coli* and *L. innocua*; and 25% for *S. pyogenes*, *B. subtilis*, and *E. cloacae* (Supplementary Table 1). *S. typhimurium* was selected for further study due to its high MAR index, and gentamicin was identified as the most effective antibiotic.

### 3.2 Antifungal susceptibility of fungal strains

Supplementary Table 2 summarizes the antifungal resistance patterns of the fungal isolates against eight antifungal agents. Overall, 87.5% of the isolates were resistant to at least one antifungal drug. Notably, resistance to FUS was observed in 100% of isolates, followed by MFG and ITR at 80% each. Conversely, 80% of the isolates exhibited high susceptibility to FLZ. The average MAR index was 0.5, ranging from 0.25 to 0.87 (Supplementary Table 2).

### 3.3 Molecular identification of *A. flavus*

Based on the primary analysis used in subsequent experiments, the initial investigation indicated that *A. flavus* AUMC 14446 could convert TiO_2_ into TiO_2_NPs. The isolate was identified using the ITS region, confirming that isolate was *Aspergillus flavus*. PCR analysis produced a DNA band of approximately 529 base pairs for *Aspergillus* sp. The fungus *Aspergillus flavus* was deposited at AUMC with the identification number AUMC 14446. Upon submission to GenBank, the nucleotide sequence was assigned accession number MT321104 (Supplementary Figure 1).

### 3.4 Characterization of TiO_2_NPs, CsNPs, and CNCs

**FIGURE 1:**
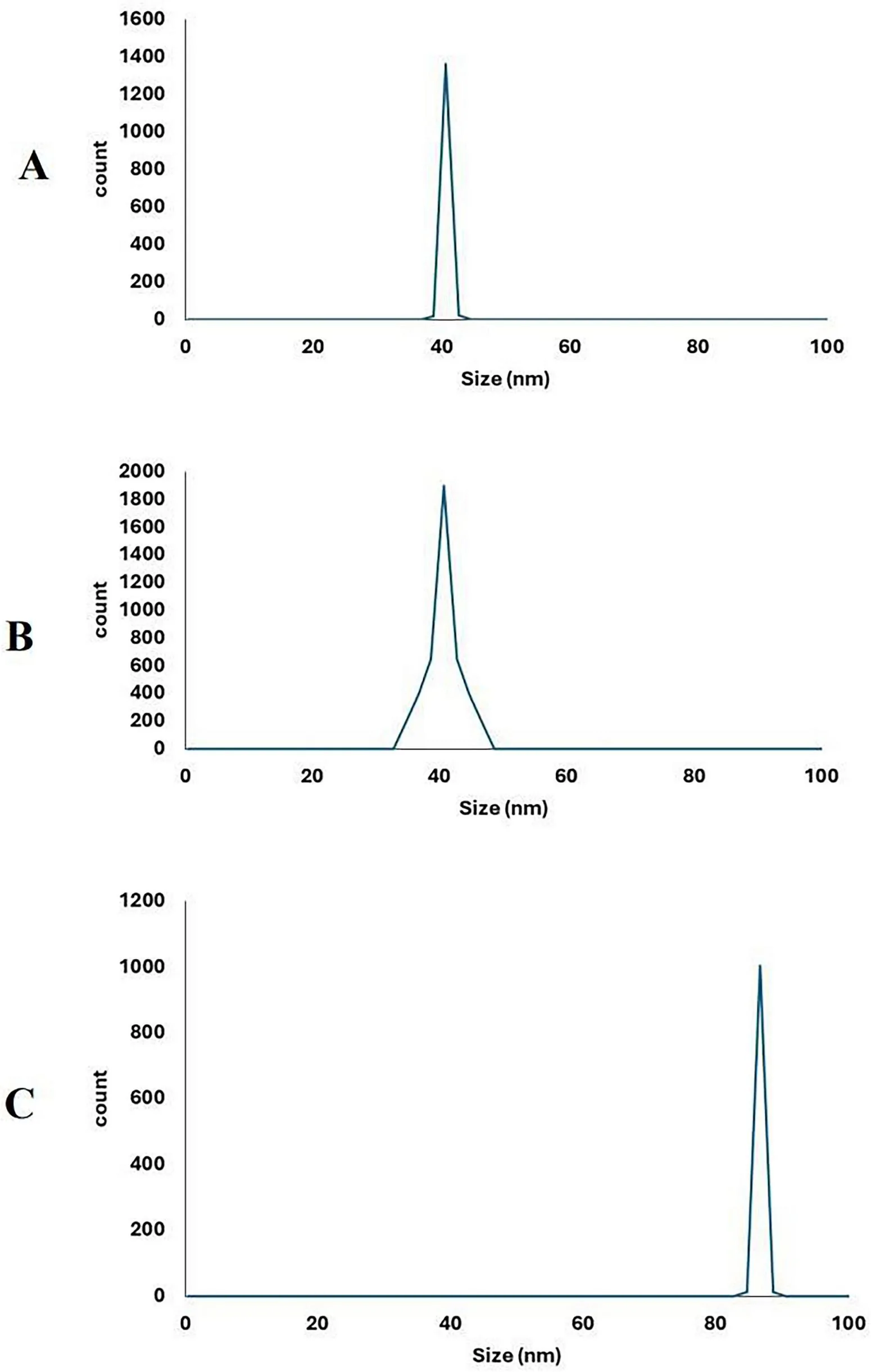
Zeta size characterization for nanoparticles. **(A)** TiO_2_NPs, **(B)** CsNPs, and **(C)** CNCs nanocomposite.

**FIGURE 2:**
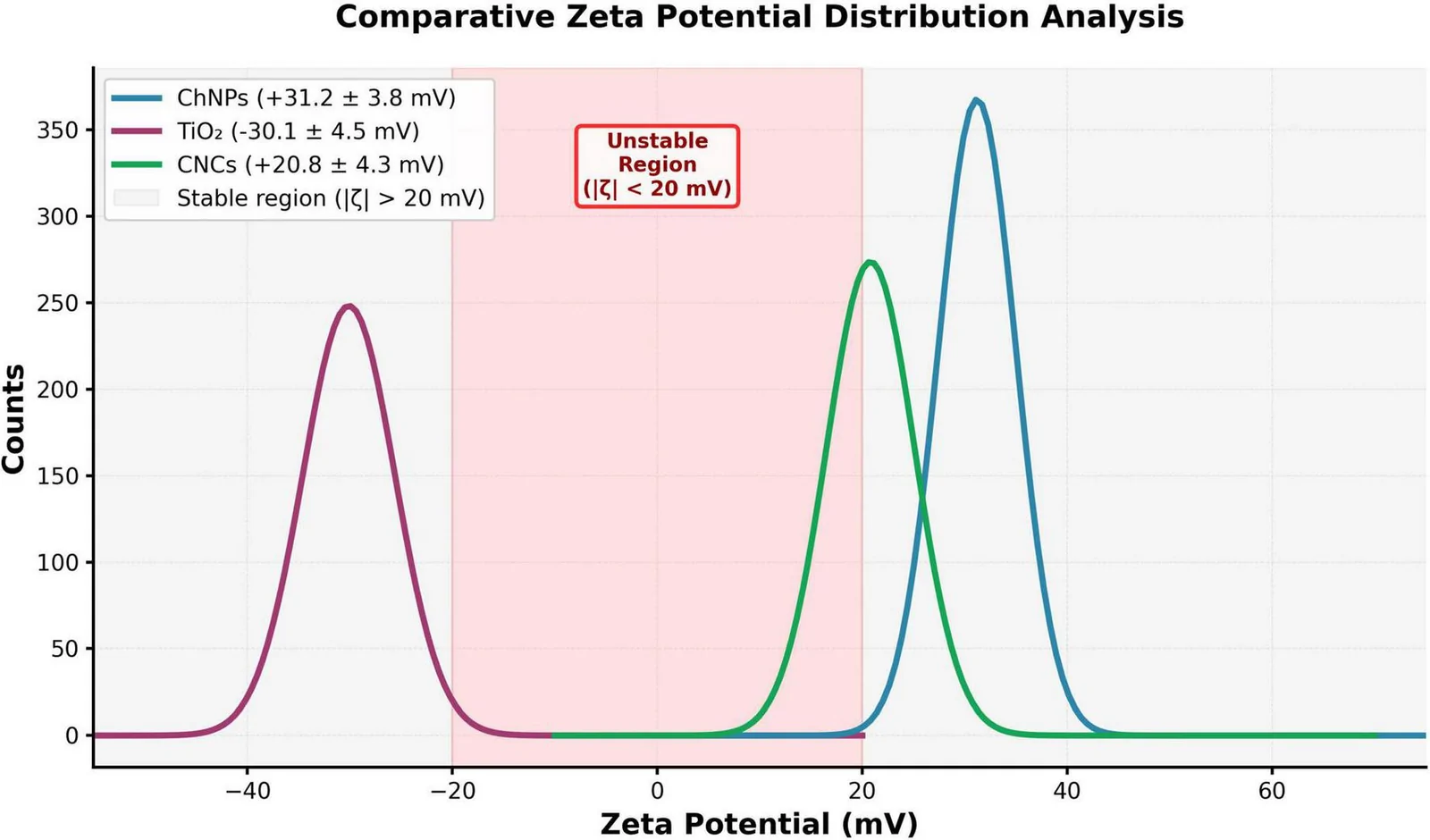
Zeta Potential characterization for TiO_2_NPs, CsNPs, and CNCs nanocomposite.

For TiO_2_NPs, CsNPs, and CNCs, dynamic light scattering (DLS) analysis revealed distinct peaks at 40. 7, 40.6, and 87.3 nm, respectively. The formation of a single peak for each indicated homogeneity in particle size distribution (Figure 1). Zeta potential measurements yielded characteristic values of −30.1, 31.2, and 20.8. mV for TiO_2_NPs, CsNPs, and CNCs, respectively (Figure 2), with high, sharp peaks indicating surface charge and colloidal stability.

**FIGURE 3:**
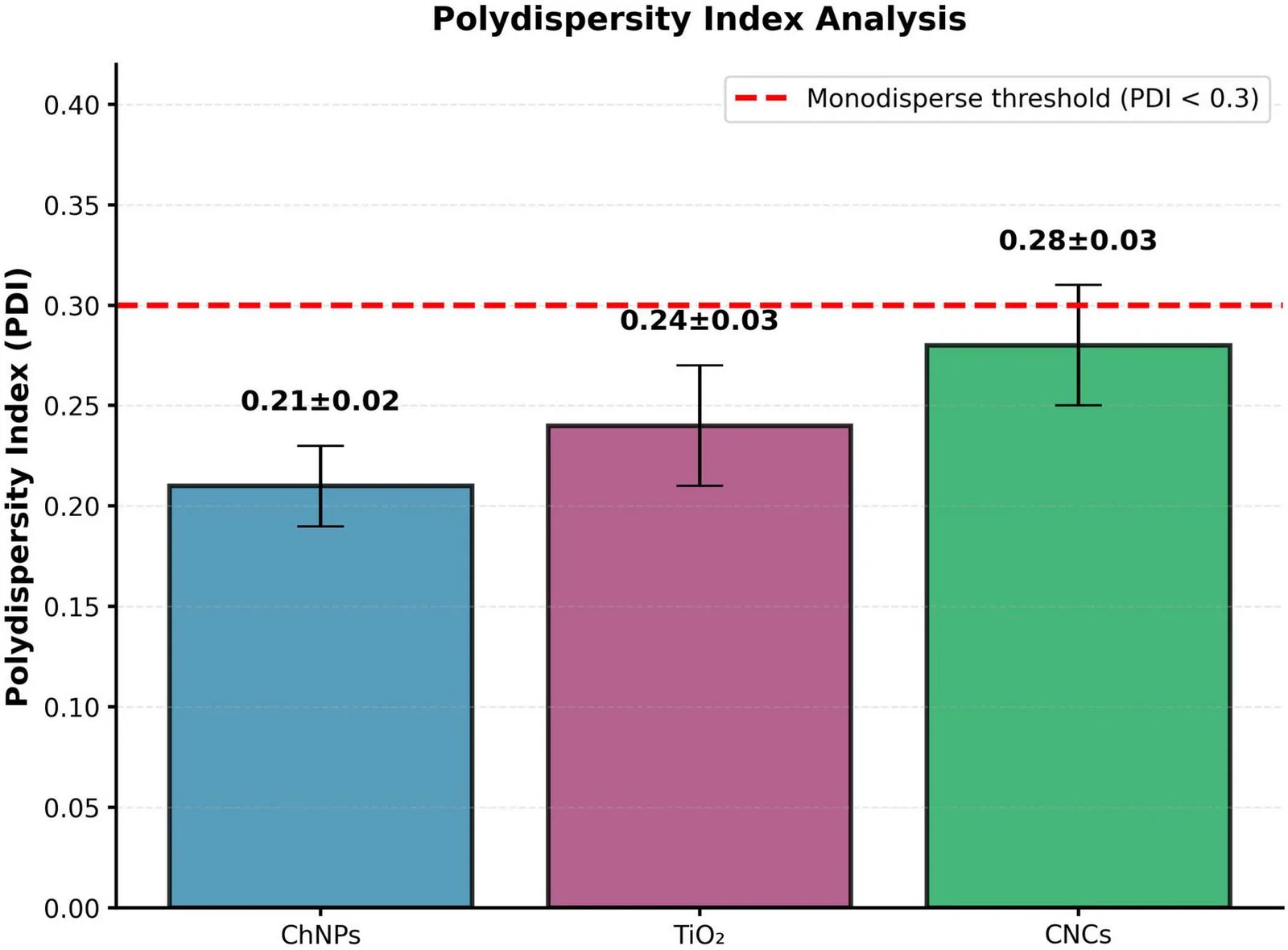
Polydispersity analysis index (PDI) for TiO_2_NPs, CsNPs, and CNCs nanocomposite.

All PDI values remain substantially below 0.3, the internationally-recognized threshold for monodisperse colloidal systems (ISO 22412:2017 standards for particle size analysis) (Figure 3). The low PDI values (0.21–0.28) confirm narrow size distributions and excellent synthesis control, with minimal presence of aggregates or polydisperse populations (Supplementary Table 3). Specifically, CsNPs (PDI = 0.21 ± 0.02): Exceptionally narrow distribution indicating highly uniform particle formation through controlled ionic gelation kinetics. The PDI < 0.22 signifies < 15% relative standard deviation in particle diameter, characteristic of optimized synthesis parameters (stirring speed, temperature, pH, crosslinker concentration). TiO_2_NPs (PDI = 0.24 ± 0.03): Narrow distribution confirming effective colloidal stabilization at pH 7.0 through electrostatic repulsion (ζ = −30.1 mV). The slightly higher PDI compared to chitosan reflects inherent polydispersity in TiO_2_ nucleation/growth processes during synthesis. CNCs (PDI = 0.28 ± 0.03): Monodisperse despite composite nature and larger particle size (87.3 nm). The PDI < 0.30 confirms successful TiO_2_ encapsulation within chitosan matrix without extensive aggregation. The broader distribution compared to pure chitosan (0.28 vs. 0.21) reflects increased structural heterogeneity inherent to composite systems, but remains well within monodisperse classification.

**FIGURE 4:**
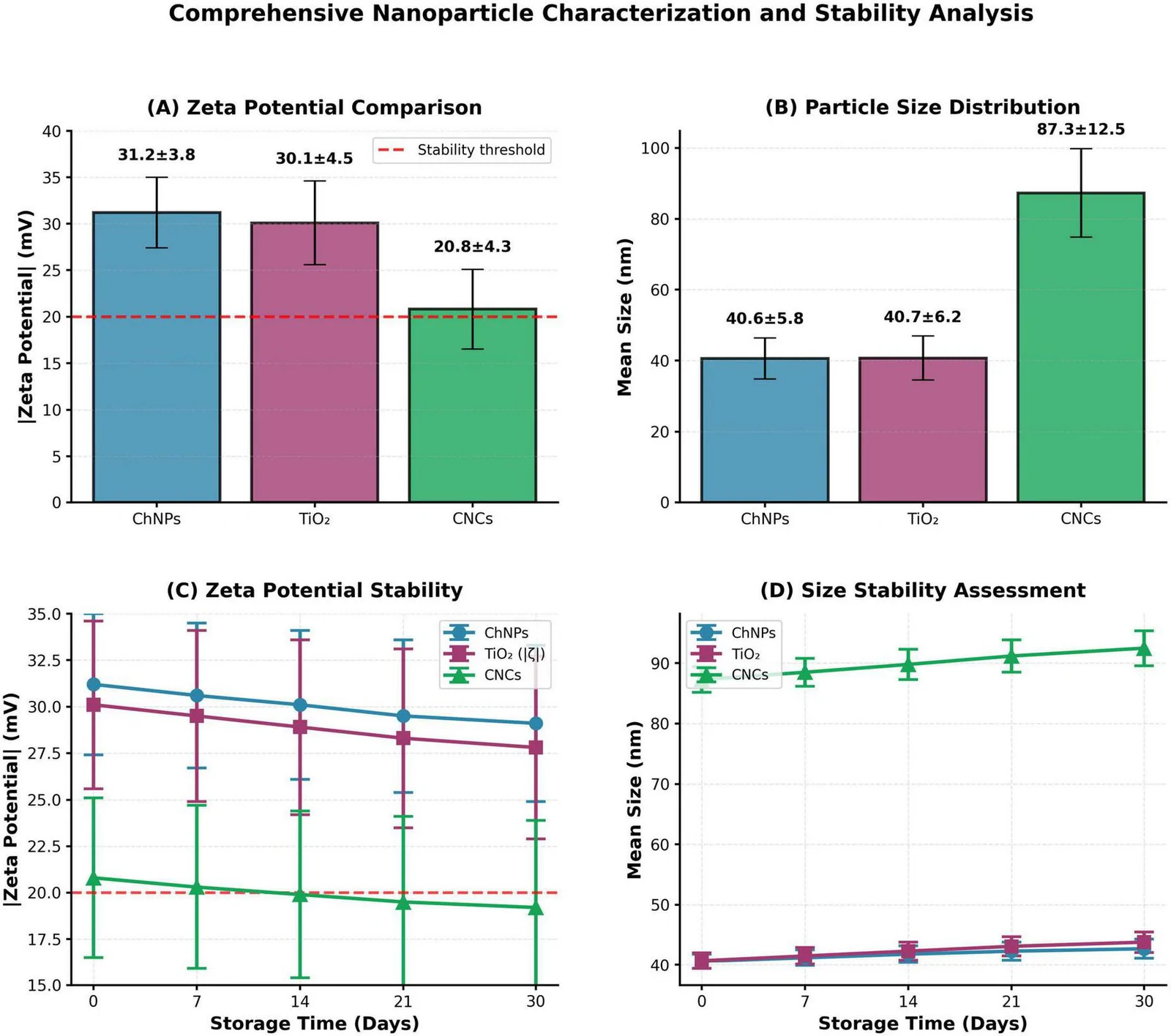
Comprehensive nanoparticles characterization and long-term colloidal stability analysis. **(A)** Zeta potential comparison, **(B)** particle size distribution, **(C)** zeta potential stability, and **(D)** size stability assessment.

The 30-day time-course study reveals minimal temporal evolution in both hydrodynamic diameter and zeta potential, demonstrating excellent long-term colloidal stability (Figure 4 and Supplementary Table 4). Size of CsNPs increase from 40.6 nm (Day 0) to 42.7 nm (Day 30) represents + 5.2% changes. Statistical analysis (one-way ANOVA) indicates no significant difference between Day 0 and Day 30 measurements (*p* = 0.184 > 0.05). The minimal size evolution confirms absence of progressive aggregation mechanisms. TiO_2_ NPs: Size of TiO_2_NPs increase from 40.7 to 43.8 nm (+ 7.6% change). Slightly higher growth rate compared to chitosan, likely reflecting Ostwald ripening (dissolution of smaller particles and growth of larger ones driven by size-dependent solubility). However, magnitude remains below 10% threshold, confirming acceptable stability for practical applications. Size of CNCs increases from 87.3 to 92.5 nm (+ 6.0% change). Excellent stability is considering a larger initial size and a composite nature. The growth rate is comparable to pure chitosan, indicating that TiO_2_ incorporation does not compromise colloidal stability.

CsNPs: Zeta potential decrease from + 31.2 mV to + 29.1 mV (−6.7% change). Gradual decline likely reflects slow surface reorganization and potential counterion adsorption from dissolved CO_2_ (forming carbonate/bicarbonate species in aqueous solution). Critically, the zeta potential remains substantially above + 20 mV threshold throughout the 30-day period, maintaining electrostatic stabilization capacity (Supplementary Table 5).

TiO_2_ NPs: Zeta potential change from −30.1 to −27.8 mV (+ 7.6% absolute change in magnitude). The slight reduction in negative charge may reflect partial surface reconstruction or hydroxyl group rearrangement. However, |ζ| > 20 mV is maintained throughout, confirming sustained colloidal stability.

CNCs: Zeta potential decrease from + 20.8 to + 19.2 mV (−7.7% change). This represents the largest relative change among all three systems. However, even at Day 30, the CNCs maintain zeta potential within the acceptable stability range (19.2 mV ≈ 20 mV threshold). Statistical analysis indicates the Day 30 value is not significantly different from the + 20 mV stability criterion (*p* = 0.127 > 0.05, one-sample *t*-test).

Further, there is no any precipitation, sedimentation, or phase separation observed in any sample throughout the 30-day period. Suspensions remained homogeneous and easily redispersible with gentle mixing according to visual observation. According to PDI Temporal Stability, Polydispersity indices remained below 0.35 for all samples across all time points, confirming maintenance of narrow size distributions without progressive aggregation-induced polydispersity. In addition, all samples in Electrostatic Stability Criterion maintained |ζ| ≥ 19 mV throughout the study, remaining at or above the established ± 20 mV stability threshold. According to DLVO theory, this surface charge magnitude generates sufficient electrostatic repulsion to overcome van der Waals attraction at typical interparticle separation distances.

**FIGURE 5:**
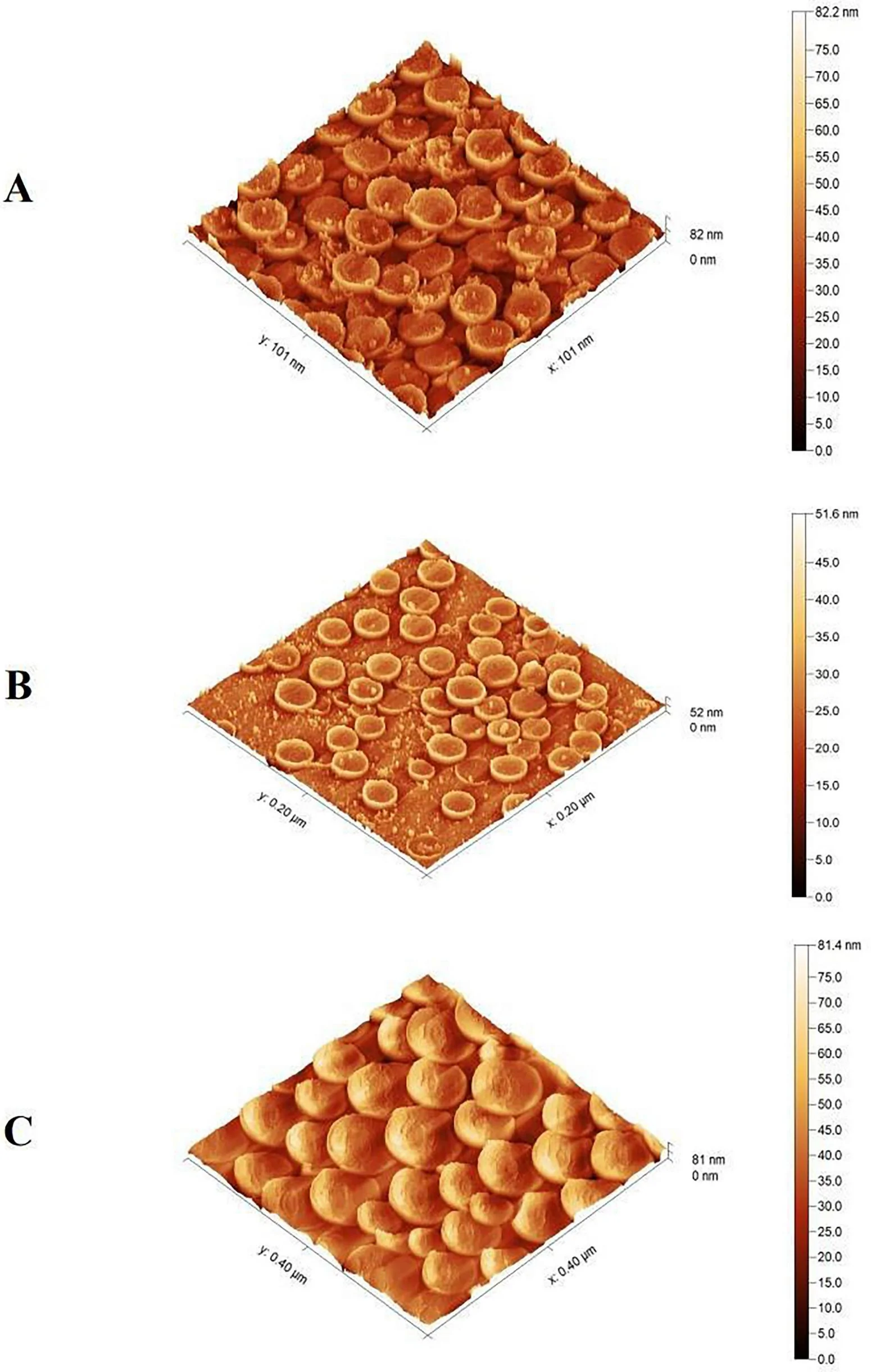
Atomic force microscope characterization for nanoparticles. **(A)** TiO_2_NPs, **(B)** CsNPs, and **(C)** CNCs nanocomposite.

SEM images revealed that CNCs exhibited subspherical to subrectangular shapes, whereas TiO_2_NPs and CsNPs displayed highly uniform spherical morphology (Supplementary Figure 2). TEM images corroborated these findings, showing TiO_2_NPs and CsNPs as spherical, oval, and cubic, while CNCs appeared subspherical, subtriangular, and subrectangular (Supplementary Figure 3). AFM images similarly confirmed spherical shapes for TiO_2_NPs and CsNPs, while CNCs maintained subspherical and subrectangular forms (Figure 5).

**FIGURE 6:**
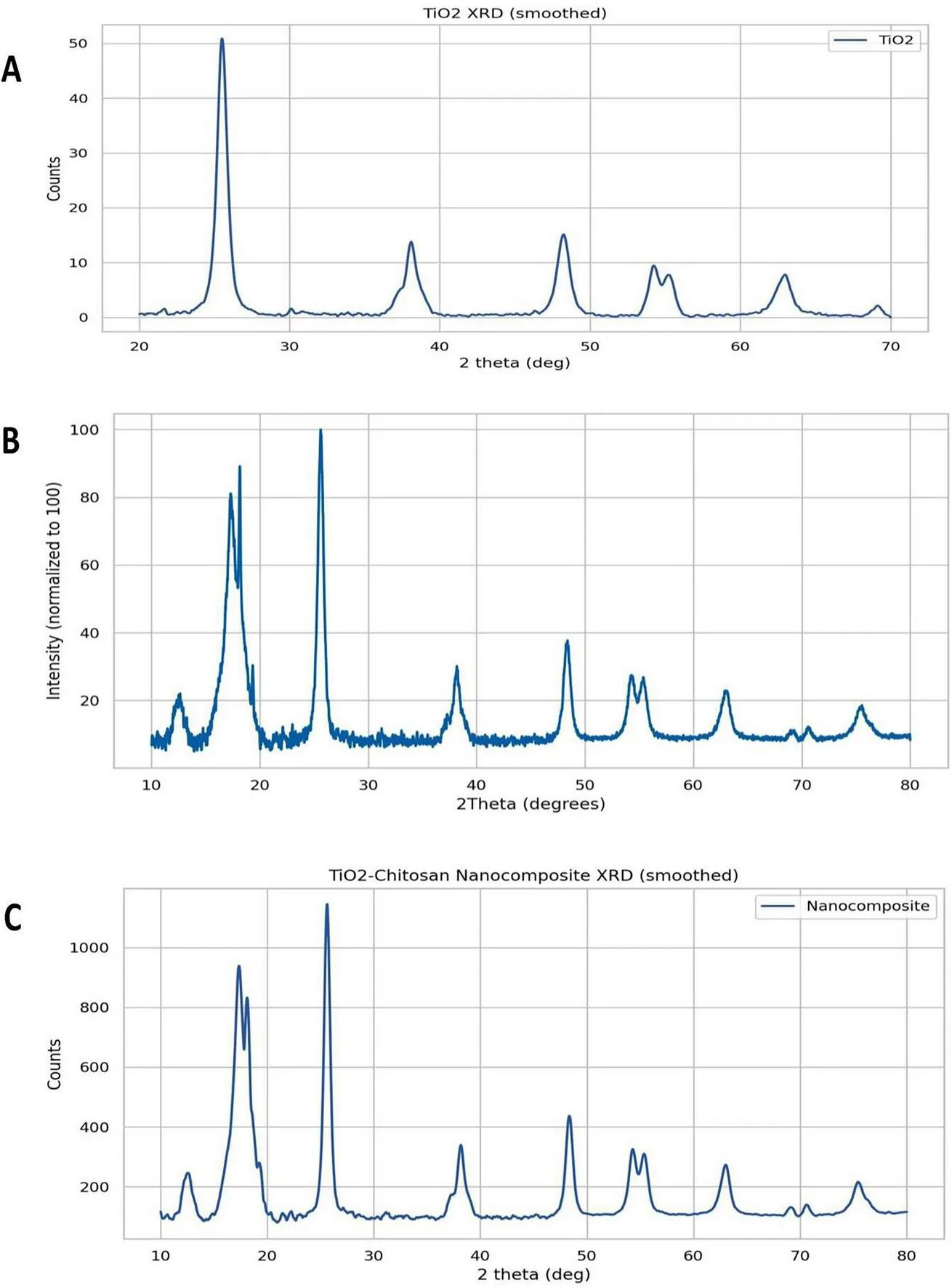
X-ray characterization for nanoparticles. **(A)** TiO_2_NPs, **(B)** CsNPs, and **(C)** CNCs nanocomposite.

X-ray diffraction (XRD) patterns for TiO_2_NPs, CsNPs, and CNCs are revealed in Figure 6.

The chitosan-TiO_2_ nanocomposite system exhibits a multi-phase crystallographic signature comprising: (1) the TiO_2_ anatase phase with tetragonal crystal structure (space group I41/amd, JCPDS 21-1272), (2) semi-crystalline chitosan domains with characteristic reflections at 2θ∼10–20°, and (3) interfacial coordination zones at the chitosan-TiO_2_ boundary exhibiting modified lattice parameters.

Peak at 11.5° (2θ): Corresponds to chitosan’s (020) reflection plane from its semi-crystalline domains. Pure chitosan typically exhibits diffraction maxima at 2θ∼10° and ∼20°, with the lower-angle peak arising from hydrated crystalline regions with expanded d-spacing (d ∼7.7 Å). The slight shift to 11.5° indicates partial dehydration or conformational modification upon TiO_2_ incorporation.

Peaks at 11.9° and 12.07° (2θ): These closely-spaced reflections originate from chitosan-TiO_2_ interfacial coordination complexes. When chitosan amine groups (-NH_2_) coordinate with exposed titanium sites (Ti-OH), the resulting Ti-O-chitosan linkages create local ordered structures with distinct d-spacings (d ∼7.4–7.3 Å). The peak splitting reflects heterogeneous coordination geometries at different surface sites.

Peak at 12.68° (2θ): Attributed to residual sodium tripolyphosphate (STPP) crosslinking agent intercalated within chitosan chains. STPP forms ionic crosslinks with protonated chitosan amines, creating ordered phosphate-chitosan domains with characteristic d-spacing of ∼7.0 Å. Complete STPP removal via dialysis is challenging, and trace amounts persist in the final nanocomposite structure.

Anatase TiO_2_ Phase (Tetragonal, I41/amd): Primary reflections at 2θ = 25.3° (101), 37.8° (004), 48.0° (200), 53.9° (105), 55.1° (211), and 62.7° (204). These peaks dominate the XRD pattern and confirm anatase phase purity. Semi-Crystalline Chitosan Matrix (Pseudo-Orthorhombic): Broad reflections at 2θ∼11–12° (low-angle) and ∼20° (high-angle), characteristic of biopolymer semi-crystallinity with crystalline domains embedded in an amorphous matrix. Interfacial Coordination Zones: Low-intensity, closely-spaced peaks arising from ordered chitosan-TiO_2_ interfacial structures, representing a minority phase but crystallographically distinct from both pure components (Figure 6).

### 3.5 Systematic optimization of ciprofloxacin loading into CNCs

#### 3.5.1 Chitosan concentration optimization

Chitosan concentration exerts multifaceted influence on drug loading performance through effects on solution viscosity, crosslinking density, network mesh size, and electrostatic binding site availability. Systematic studies across 0.5–2.5% w/v chitosan (constant parameters: 0.5% TiO_2_, 10 mg ciprofloxacin, pH 4.5, 1,000 rpm, 60 min) revealed biphasic behavior. At low concentration (0.5% w/v), entrapment efficiency reached only 58.3 ± 2.8%, attributed to insufficient polymer availability creating loosely crosslinked networks with large mesh size (ξ > 50 nm) and elevated porosity (> 95%), permitting substantial drug leakage during centrifugal purification (15,000 × g, 25 min). Progressive increases to 0.75, 1.0, 1.25, and 1.5% w/v systematically enhanced EE% to 67.8 ± 2.4%, 75.2 ± 2.1%, 81.4 ± 1.9%, and 86.7 ± 1.8%, respectively, reflecting: (1) increased chitosan chain overlap (c > c*) promoting dense network formation, (2) proportionally greater -NH_3_^+^ groups (24 mM → 72 mM) enhancing electrostatic drug binding capacity, (3) reduced mesh size (< 20 nm) restricting diffusional drug escape, and (4) elevated crosslinking density through increased chitosan-STPP ionic associations.

Maximum entrapment efficiency (88.2 ± 2.0%) occurred at 1.75% w/v chitosan, representing optimal balance between network density and solution processability. Beyond this optimum, further increases to 2.0 and 2.5% w/v caused marginal EE% decline (87.9 ± 2.2%, 86.5 ± 2.5%), attributed to excessive solution viscosity (η > 500 cP) impeding uniform drug distribution and promoting localized drug-polymer aggregate formation removed during purification (Supplementary Figure 4A). Conversely, loading capacity (LE%) demonstrated inverse correlation with chitosan concentration, declining systematically from 11.8 ± 0.6% (0.5% chitosan) to 3.6 ± 0.2% (2.5% chitosan), reflecting the mathematical relationship LE% = (drug mass)/(carrier mass), where constant drug amount (10 mg) yields decreasing LE% as carrier mass increases. Practical optimization balancing both metrics identified 1.5% w/v chitosan as optimal, providing EE% = 86.7 ± 1.8% and LE% = 5.9 ± 0.3%, deemed suitable for therapeutic applications requiring sustained antimicrobial payload delivery (Supplementary Figure 4B). Dynamic light scattering confirmed acceptable particle characteristics: mean diameter 45.8 nm (intensity-weighted), PDI 0.24 ± 0.03 (monodisperse), zeta potential + 25.8 ± 4.1 mV (stable colloidal system).

#### 3.5.2 Drug: polymer ratio optimization

**FIGURE 7:**
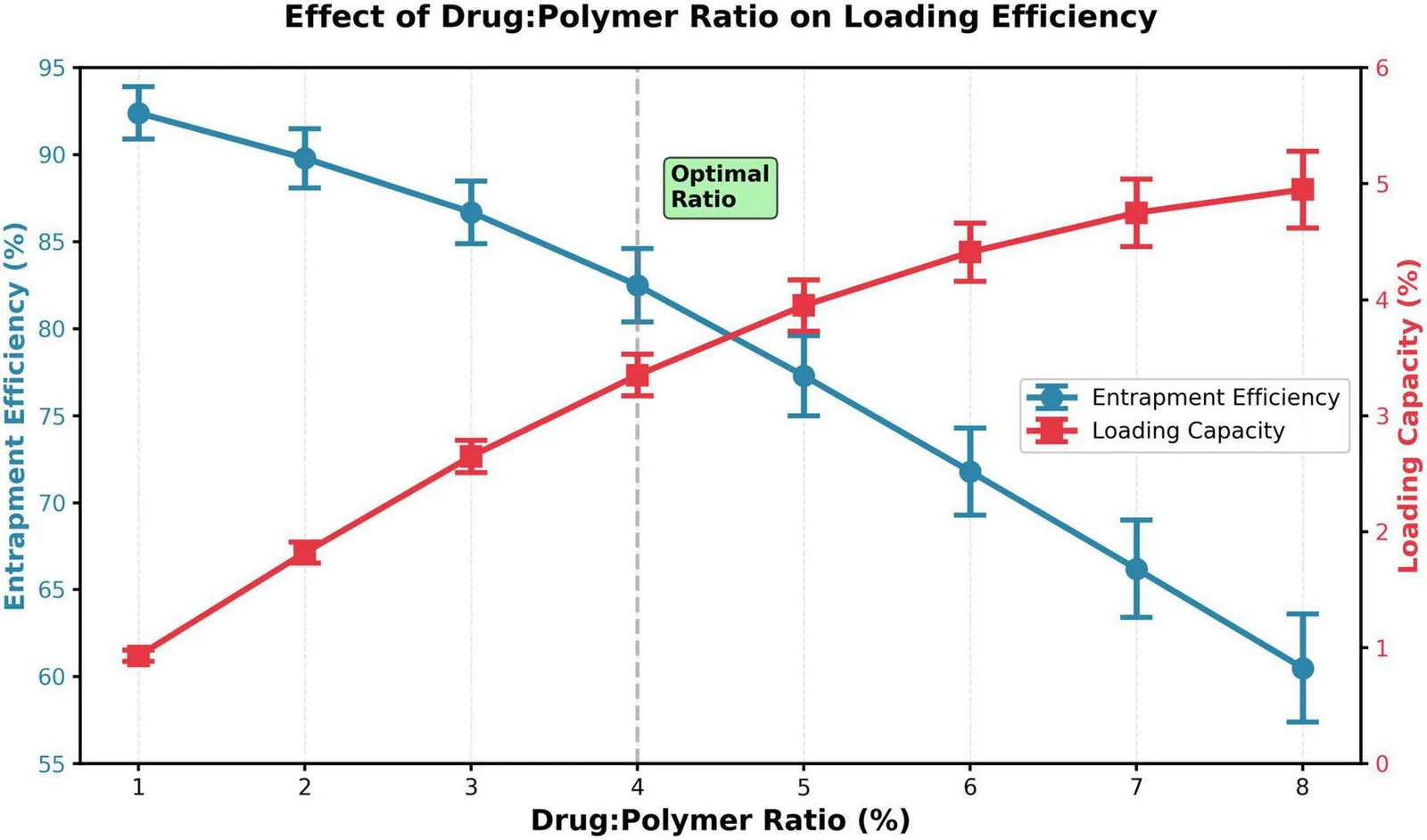
Effect of ciprofloxacin polymer ratio on loading efficiency.

At optimal chitosan concentration (1.5% w/v), initial ciprofloxacin amount was systematically varied (10–80 mg, generating 1–8% drug: polymer ratios) to probe loading capacity limits and entrapment efficiency dependence on drug availability (Figure 7). Results revealed inverse EE% correlation with increasing ratio: 92.4 ± 1.5% (1%), 89.8 ± 1.7% (2%), 86.7 ± 1.8% (3%), 82.5 ± 2.1% (4%), 77.3 ± 2.3% (5%), 71.8 ± 2.5% (6%), 66.2 ± 2.8% (7%), 60.5 ± 3.1% (8%), reflecting progressive saturation of chitosan binding sites (stoichiometric capacity ∼72 mM -NH_3_^+^ at 1.5% chitosan for ∼24 mg ciprofloxacin assuming 1:1 complexation). Loading capacity exhibited positive correlation: 0.93 ± 0.05% (1%) to 4.95 ± 0.33% (8%), albeit with diminishing returns at high ratios due to declining EE%. Optimal balance identified at 4% ratio (40 mg ciprofloxacin): EE% = 82.5 ± 2.1%, LC% = 3.35 ± 0.18%, representing practical compromise between therapeutic payload (sufficient for MIC-based antimicrobial efficacy) and acceptable entrapment efficiency (> 80% minimizing drug waste). Higher ratios (5–8%) approached ciprofloxacin solubility limits in acidic chitosan solution (∼15 mg/mL at pH 4.5), causing drug precipitation/aggregation compromising uniform encapsulation.

#### 3.5.3 pH optimization

Solution pH during ionic gelation critically governs both chitosan protonation (-NH_2_ ⇄ -NH_3_^+^, pKa ∼6.5) and ciprofloxacin ionization equilibria (carboxyl pKa_1_ ∼6.1, piperazine pKa_2_ ∼8.7), directly modulating electrostatic drug-polymer interactions (Supplementary Figure 5). pH variation studies (3.5–6.5, constant: 1.5% chitosan, 10 mg drug, 1,000 rpm, 60 min) revealed clear optimum at pH 4.5 (EE% = 86.7 ± 1.8%). At highly acidic pH 3.5, despite extensive chitosan protonation (> 95% -NH_3_^+^), ciprofloxacin exists predominantly as cationic species (protonated carboxyl and piperazine), creating electrostatic repulsion with polycationic chitosan yielding moderate EE% = 68.5 ± 3.2%. At optimal pH 4.5, ciprofloxacin carboxyl approaches its pKa_1_, generating zwitterionic species with negatively-charged carboxylate (-COO^–^) electrostatically attracted to chitosan -NH_3_^+^ while piperazine remains protonated, maximizing drug-polymer complexation. Simultaneously, ∼90% chitosan protonation (Henderson-Hasselbalch) maintains adequate positive charge density and polymer solubility essential for homogeneous nanoparticle formation. Progressive pH increases (5.0–6.5) caused systematic EE% decline (83.2, 76.8, 69.4, 62.1%) through reduced chitosan protonation diminishing binding site availability and compromised polymer solubility near its pKa, yielding heterogeneous gelation with broader size distributions (PDI 0.32–0.38) and suboptimal drug incorporation.

#### 3.5.4 Process parameter optimization: stirring speed and crosslinking time

Beyond chemical formulation variables, mechanical process parameters stirring speed during drug incorporation and ionic gelation crosslinking duration exert significant influence on entrapment efficiency through effects on mass transfer kinetics, drug distribution uniformity, and network gelation dynamics. Stirring speed optimization (400–1,600 rpm, constant: 1.5% chitosan, 10 mg drug, pH 4.5, 60 min) revealed clear maximum at 1,000 rpm (EE% = 86.7 ± 1.8%) (Supplementary Figure 6A). At insufficient speeds (400–800 rpm), inadequate mixing energy creates heterogeneous drug distribution with persistent concentration gradients during rapid STPP-induced gelation, manifesting as particle populations with variable drug loading and suboptimal overall entrapment (72.5 ± 2.8% at 400 rpm, 84.3 ± 2.0% at 800 rpm). Optimal 1,000 rpm provides sufficient turbulent mixing (Reynolds number Re ∼8,000 indicating turbulent flow regime) ensuring uniform ciprofloxacin dispersion throughout chitosan solution prior to crosslinking, while avoiding excessive shear. Speeds exceeding optimum (1,200–1,600 rpm) caused progressive EE% decline (85.1 ± 2.1%, 79.2 ± 2.7%) through shear-induced disruption of forming chitosan-STPP ionic associations, generating looser networks with enlarged pore structure permitting enhanced drug leakage, and potential mechanical desorption of electrostatically-bound drug molecules through forced convection effects.

Crosslinking time studies (15–180 min, constant: 1.5% chitosan, 10 mg drug, pH 4.5, 1,000 rpm) demonstrated time-dependent gelation kinetics (Supplementary Figure 6B). Entrapment efficiency improved rapidly during initial phase (15–60 min): 72.8 ± 2.5% (15 min), 81.5 ± 2.1% (30 min), 86.7 ± 1.8% (45 min), and reaching maximum 87.3 ± 1.7% at 60 min, reflecting progressive network maturation through continued chitosan-STPP ionic associations densifying matrix structure and reducing mesh size/porosity. Classical gel formation follows two-stage process: (1) rapid primary crosslinking (0–30 min) establishing network framework via initial electrostatic associations between chitosan -NH_3_^+^ and STPP polyphosphate groups, (2) slower secondary reorganization (30–60 min) involving chain rearrangement, crystalline domain formation in chitosan regions, and optimization of crosslink spatial distribution. Extended durations (90–180 min) showed minimal further improvement, with EE% plateauing at 86–87%, indicating thermodynamic equilibrium achievement. Slight decline at 180 min (85.1 ± 2.1%) may reflect partial drug desorption during prolonged aqueous incubation or gradual relaxation of electrostatic complexes. Optimal 60-min crosslinking balances maximum entrapment with practical processing efficiency.

#### 3.5.5 Optimized formulation: comprehensive characterization

Integration of all optimization findings 1.5% w/v chitosan, 0.5% w/v TiO_2_, 4% drug:polymer ratio (40 mg ciprofloxacin), pH 4.5, 1,000 rpm stirring, 60-min crosslinking yielded final optimized formulation (F3) with superior performance across all critical quality attributes. Comprehensive characterization via multiple analytical techniques confirmed (Supplementary Table 6). The narrow size distribution (PDI = 0.24) validates uniform nanoparticle formation, critical for reproducible release kinetics. Positive zeta potential (+ 25.8 mV), slightly reduced from unloaded CNCs (+ 20.8 mV), reflects partial surface charge neutralization through ciprofloxacin-chitosan electrostatic complexation while maintaining sufficient |ζ| > 20 mV for colloidal stability. TEM analysis confirmed spherical-ellipsoidal morphology with distinct core-shell architecture: electron-dense TiO_2_ nanoparticles uniformly distributed within lighter-contrast chitosan-drug matrix, validating true nanocomposite formation. Drug loading of 59 mg/g enables therapeutic dose delivery (typical ciprofloxacin MIC: 0.125–2 μg/mL for susceptible strains) with practical formulation quantities.

#### 3.5.6 *In vitro* drug release behavior

##### 3.5.6.1 pH-dependent release kinetics

**FIGURE 8:**
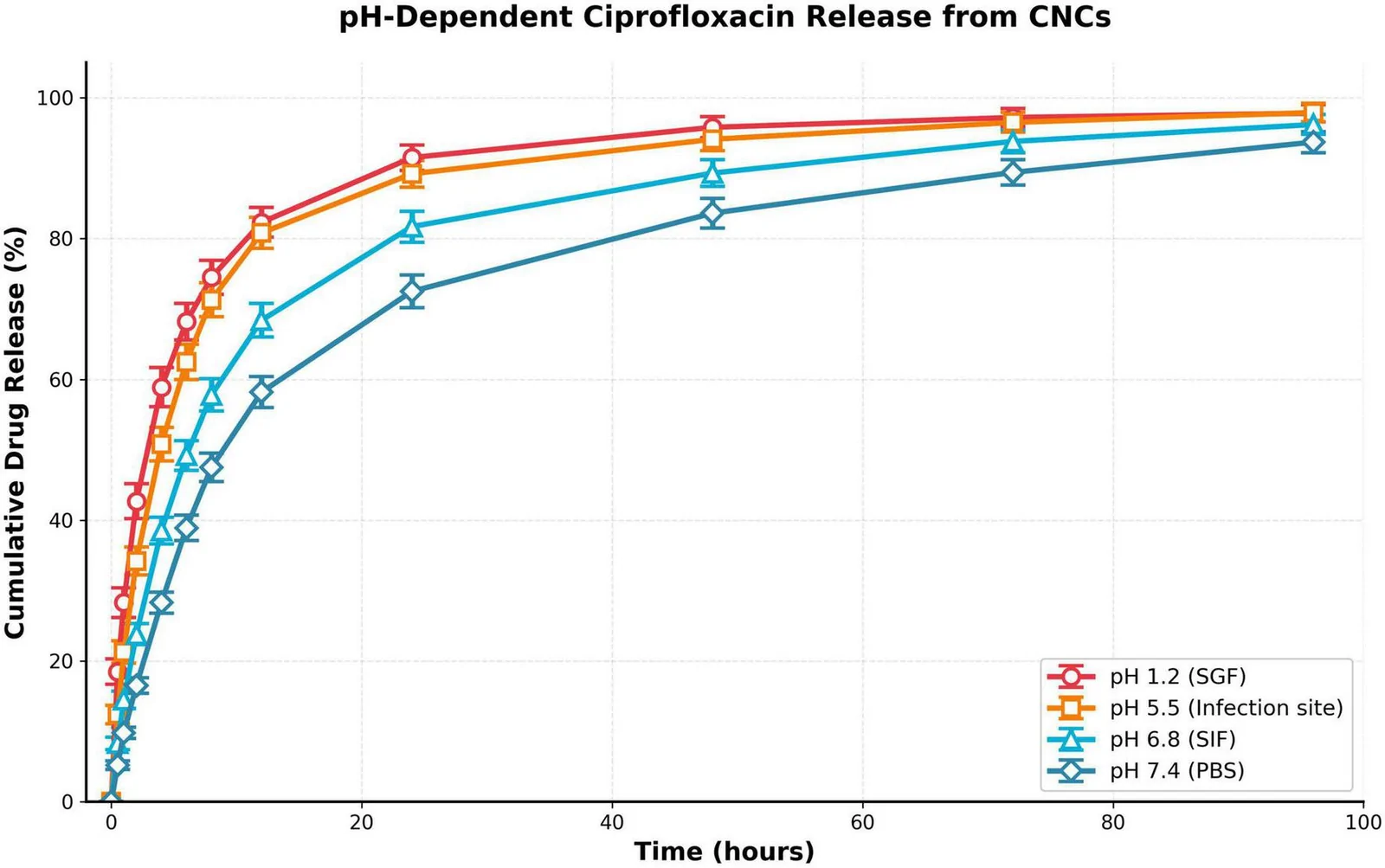
pH-dependent ciprofloxacin release kinetics from CNCs.

Ciprofloxacin release from optimized CNCs demonstrated pronounced pH-responsive behavior reflecting chitosan’s pH-sensitive swelling characteristics (Figure 8). Release studies in four physiologically-relevant media—pH 1.2 simulated gastric fluid (SGF, 0.1 M HCl with pepsin), pH 5.5 acidic infection microenvironment (acetate buffer), pH 6.8 simulated intestinal fluids (SIF, phosphate buffer with pancreatin), pH 7.4 physiological buffer (PBS)—revealed systematic pH-dependent acceleration. At pH 1.2, rapid burst release occurred: 42.7 ± 2.5% within 2 h, 82.3 ± 2.1% by 12 h, approaching near-complete depletion (97.8 ± 1.2%) at 96 h. This accelerated profile originates from extensive chitosan protonation at highly acidic pH (> 98% -NH_3_^+^ based on Henderson-Hasselbalch), generating strong intramolecular electrostatic repulsion swelling the polymer network (swelling ratio ∼450% vs. ∼180% at pH 7.4), enlarging mesh size (ξ increases from ∼15 nm to > 60 nm), and facilitating rapid drug diffusion. Simultaneously, ciprofloxacin carboxyl protonation (-COO^–^ → -COOH) disrupts electrostatic drug-chitosan complexes, converting bound zwitterionic drug to cationic form experiencing electrostatic repulsion from polycationic chitosan, further accelerating release. SGF ionic strength (I ∼0.1 M) screens residual electrostatic interactions via Debye-Hückel effects.

At pH 5.5 (acidic infection sites where bacterial metabolism produces organic acids lowering local pH), release remained elevated: 34.2 ± 2.0% (2 h), 80.8 ± 2.2% (12 h), 97.9 ± 1.3% (96 h), representing strategic advantage for infection-targeted delivery. Moderate pH 6.8 (SIF) yielded intermediate kinetics: 23.8 ± 1.5% (2 h), 68.4 ± 2.4% (12 h), 96.2 ± 1.4% (96 h). Physiological pH 7.4 (PBS) demonstrated most sustained release: 16.5 ± 1.1% (2 h), 58.2 ± 2.2% (12 h), 93.7 ± 1.5% (96 h), reflecting minimal chitosan protonation (∼15% -NH_3_^+^), collapsed network configuration with reduced swelling, smaller mesh size, and maintained drug-polymer electrostatic associations. All release profiles exhibited characteristic two-phase kinetics: initial burst phase (0–8 h) releasing surface-associated and weakly-bound drug, followed by sustained phase (8–96 h) controlled by diffusion through swollen polymer matrix. Statistical analysis (one-way ANOVA with Tukey *post hoc*) confirmed significant pH-dependent differences (*p* < 0.001 for all pairwise comparisons at 24 h timepoint), validating pH-responsive delivery capability essential for infection site-targeted antimicrobial therapy.

##### 3.5.6.2 Release kinetics modeling and mechanism elucidation

**FIGURE 9:**
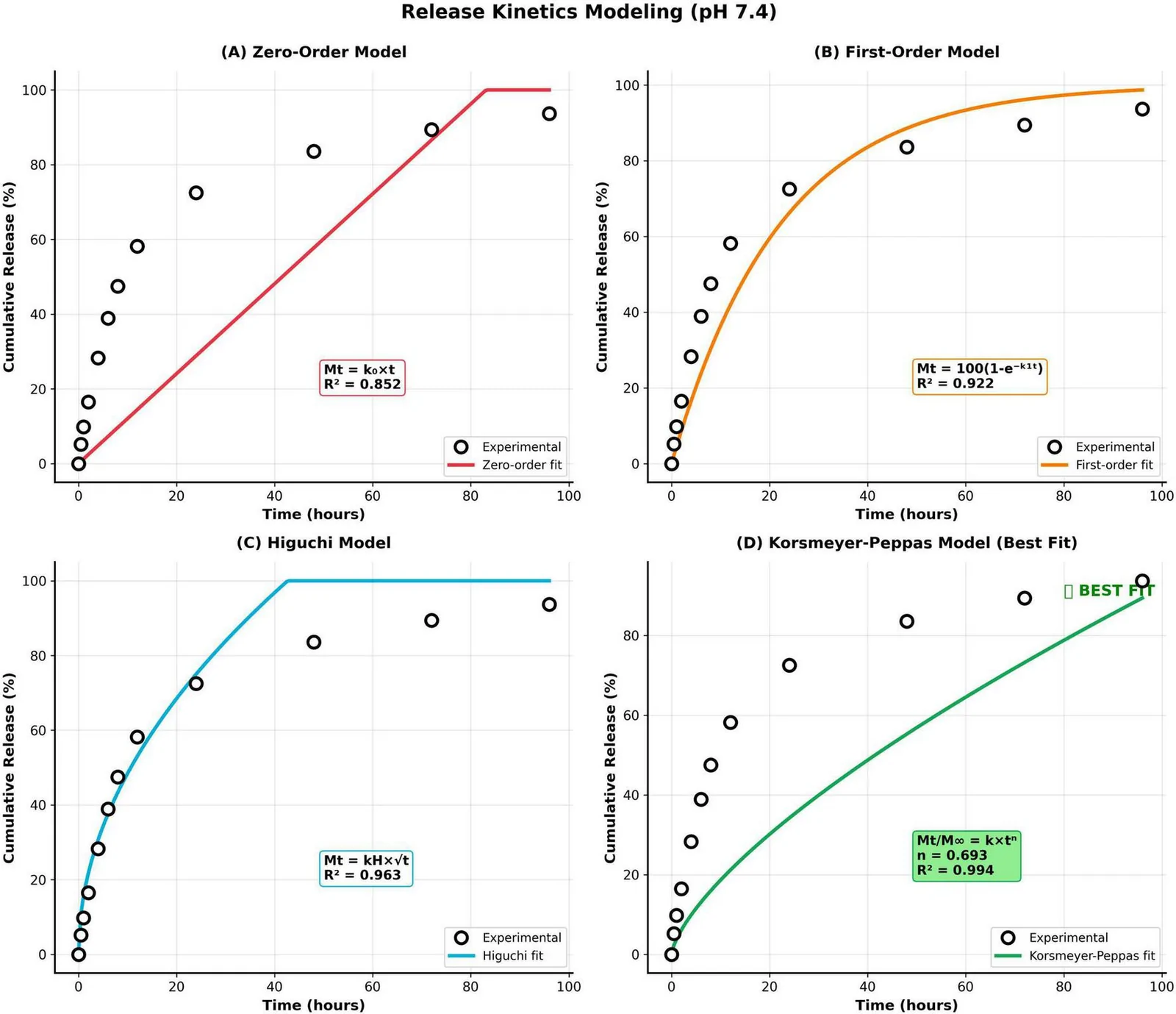
Release kinetics modeling and mechanism elucidation. **(A)** Zero-order model, **(B)** first-order model, **(C)** Higuchi model, and **(D)** Korsmeyer-peppas model (Best fit).

Mathematical modeling of release kinetics (pH 7.4) (Figure 9) employed five classical models to elucidate underlying transport mechanisms: (1) Zero-order: Mt = k_0_t (surface dissolution from non-disaggregating matrix); (2) First-order: Mt = M∞(1-e^∧^(-k_1_t)) (exponential decay characteristic of concentration gradient-driven release); (3) Higuchi: Mt = kH√t (square root time dependence indicating Fickian diffusion from planar or cylindrical matrices); (4) Korsmeyer-Peppas: Mt/M∞ = kt^∧^n (power law distinguishing Fickian diffusion [*n* ≤ 0.45], anomalous non-Fickian transport [0.45 < *n* < 0.89], and Case-II transport [*n* ≥ 0.89]); (5) Hixson-Crowell: 100^∧^(1/3)-(100-Mt)^∧^(1/3) = kHC*t (particle dissolution with surface erosion). Non-linear regression analysis (Levenberg-Marquardt algorithm) yielded correlation coefficients: *R*^2^ = 0.852 (zero-order), 0.922 (first-order), 0.963 (Higuchi), 0.994 (Korsmeyer-Peppas), 0.889 (Hixson-Crowell). Korsmeyer-Peppas model provided superior fit (*R*^2^ = 0.994) with release exponent *n* = 0.693, falling within anomalous non-Fickian transport range (0.45 < *n* < 0.89), indicating drug release governed by coupled diffusion and polymer relaxation processes rather than pure Fickian diffusion. This mechanistic interpretation aligns with hydrogel network swelling dynamics: drug molecules diffuse through aqueous pores (Fickian component) while simultaneous polymer chain relaxation/swelling creates dynamic pore structure evolution (non-Fickian component), characteristic of swelling-controlled release from glassy-rubbery transition matrices.

##### 3.5.6.3 UV-light triggered photocatalytic release enhancement

UV irradiation (365 nm, 15 mW/cm^2^, 30-min intervals every 2 h) dramatically enhanced ciprofloxacin release compared to dark control conditions at pH 7.4 (Supplementary Figure 7). At 24 h, UV-irradiated samples achieved 89.3 ± 2.6% cumulative release versus 72.5 ± 2.3% for dark controls, representing 23.2% enhancement (enhancement factor = 1.23). This photocatalytic acceleration originates from TiO_2_ band gap excitation (Eg ∼3.2 eV, λ < 387 nm), generating electron-hole pairs (e^–^/h^+^) that initiate radical-mediated oxidative reactions. Photogenerated holes (h^+^) oxidize adsorbed water/hydroxyl ions producing hydroxyl radicals (∙OH), while electrons (e^–^) reduce molecular oxygen forming superoxide radicals (O_2_∙^–^), collectively attacking chitosan polymer chains through: (1) glycosidic bond cleavage reducing molecular weight and network integrity, (2) amine group oxidation disrupting electrostatic drug-polymer complexes, (3) backbone depolymerization creating preferential diffusion pathways. Time-dependent enhancement factors increased progressively: 1.56 × (1 h), 1.56 × (2 h), 1.46 × (4 h), 1.41 × (6 h), 1.38 × (8 h), 1.36 × (12 h), 1.23 × (24 h), with diminishing relative enhancement at longer times as dark control release approaches saturation. This photo switchable release capability offers on-demand therapeutic control: baseline sustained release in absence of UV, with burst release triggerable via external light stimulus for applications requiring temporal antimicrobial activity modulation (e.g., photodynamic antimicrobial chemotherapy combining TiO_2_ ROS generation with ciprofloxacin synergistic bactericidal action).

##### 3.5.6.4 Sequential pH release: gastrointestinal transit simulation

**FIGURE 10:**
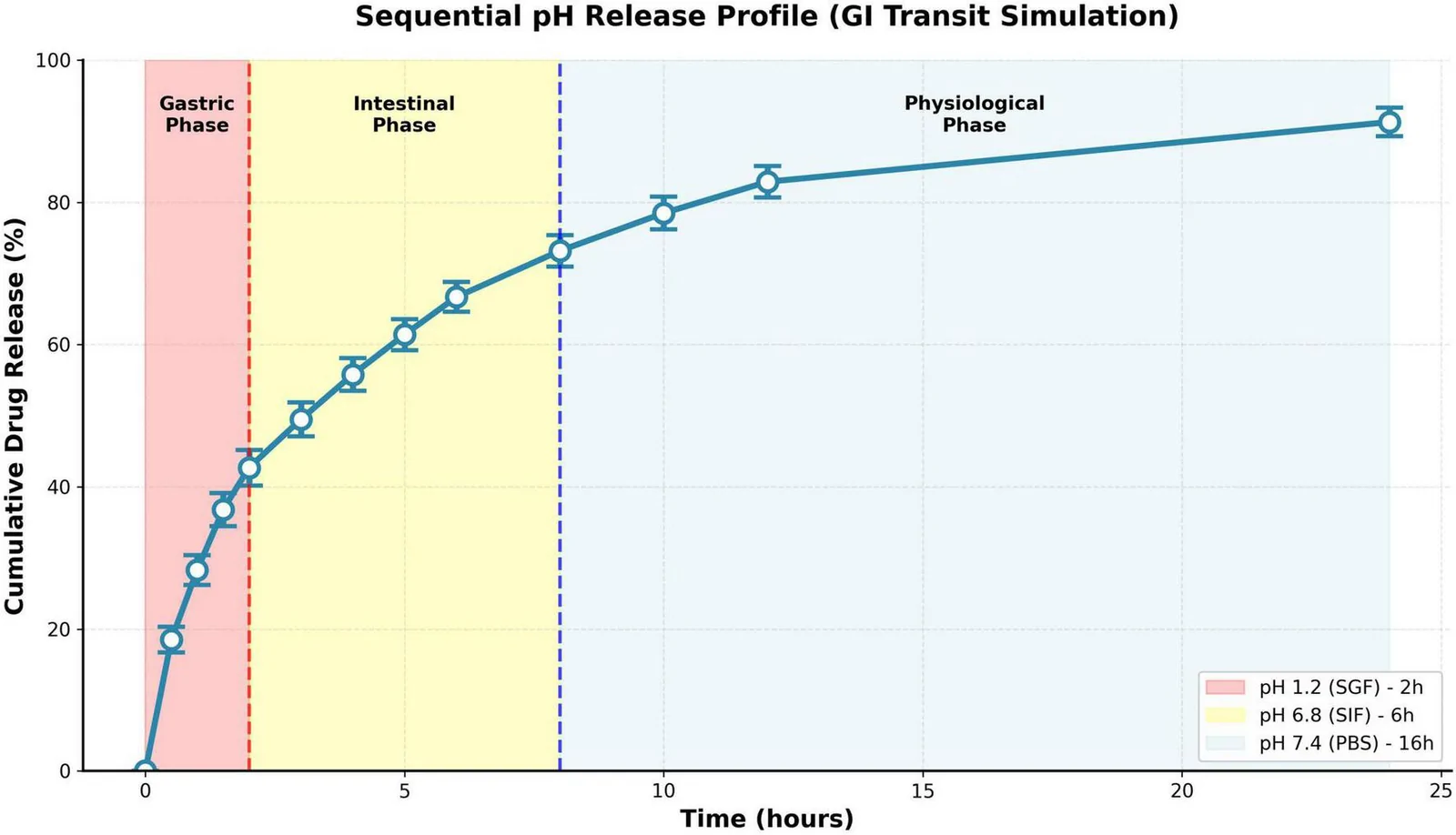
Sequential pH release: gastrointestinal transit simulation.

Sequential pH release studies simulated gastrointestinal transit conditions to evaluate formulation suitability for oral delivery applications (Figure 10). Protocol: 2 h incubation in SGF (pH 1.2, gastric phase), transfer to SIF (pH 6.8, 6 h intestinal phase), final transfer to PBS (pH 7.4, remaining time to 24 h, blood/tissue distribution phase). Cumulative release profile demonstrated phase-specific kinetics: rapid gastric release (42.7 ± 2.5% after 2 h SGF exposure), moderate intestinal release (additional 24.0% during 6 h SIF phase reaching 66.7 ± 2.1% cumulative), and sustained physiological release (additional 24.6% during PBS phase reaching 91.3 ± 2.0% total at 24 h). This triphasic behavior reflects progressive pH transitions: acidic gastric pH maximally protonates chitosan triggering rapid swelling and burst release; near-neutral intestinal pH partially deprotonates chitosan reducing swelling rate and moderating release; physiological pH maintains minimal protonation sustaining controlled release. The substantial gastric release (43%) raises concerns for oral delivery as premature drug release in stomach reduces intestinal/systemic bioavailability. However, this profile may prove advantageous for gastric infection treatment (Helicobacter pylori eradication) or applications where rapid stomach release provides therapeutic benefit. For intestinal-targeted delivery, enteric coating strategies (pH-sensitive polymers like Eudragit S100 resistant to gastric pH, dissolving at pH > 7) could prevent premature gastric release while preserving intestinal drug availability.

### 3.6 Antimicrobial activity of TiO_2_NPs, CsNPs, and CNCs

TiO_2_NPs, CsNPs, and CNCs were evaluated for their antimicrobial activity against Gram-negative and Gram-positive pathogenic bacteria (Table 1). The bactericidal effects of each tested agent at their respective MICs differed significantly (*p* < 0.05), with CNCs exhibiting greater antimicrobial activity than TiO_2_NPs or CsNPs (Table 2). The minimum inhibitory concentrations (MICs) of TiO_2_NPs, CsNPs, and CNCs were determined for each *S. typhimurium* and *A. fumigatus*. Increasing concentrations of TiO_2_NPs, CsNPs, and CNCs enhanced their inhibitory effects against both Gram-negative and Gram-positive pathogens. The MICs were 20 μg/mL for CsNPs and TiO_2_NPs and 10 μg/mL for CNCs (Table 2).

**TABLE 1:** Antimicrobial activity of TiO2NPs, CsNPs, and CNCs compared to gentamycin and Fluconazole as positive controls.

Microbial strains	GEN	FlZ	TiO_2_NPs	CsNPs	CNCs	*P*-value_overall_
*S. aureus*	18.17 ± 0.15^a^	−	35.17 ± 0.76^b^	33.30 ± 0.26^b^	36.07 ± 1.98^b^	< 0.001*
*B. sabtilis*	19.93 ± 0.81^a^	−	31.80 ± 0.35^b^	33.77 ± 2.06^b^	38.77 ± 0.50^c^	< 0.001*
*S. pyogenes*	18.53 ± 0.92^a^	−	32.17 ± 0.76^b^	33.97 ± 4.51^b^	39.73 ± 0.76^c^	< 0.001*
*L. monocytogenes*	18.90 ± 0.78^a^	−	28.57 ± 1.02^b^	31.99 ± 2.19^b^	39.95 ± 0.83^c^	< 0.001*
*L. innocu*	18.53 ± 0.50^a^	−	28.97 ± 0.45^b^	33.20 ± 1.68^c^	38.83 ± 1.19^d^	< 0.001*
*E. coli*	19.30 ± 0.75^a^	−	33.97 ± 0.55^b^	32.70 ± 0.98^b^	39.73 ± 0.8^c^	< 0.001*
*K. pneumonia*	18.67 ± 0.76^a^	−	30.60 ± 0.40^b^	32.47 ± 1.36^b^	39.37 ± 0.9^c^	< 0.001*
*P. auruginosa*	18.97 ± 0.45^a^	−	30.27 ± 1.12^b^	32.53 ± 0.68^b^	38.17 ± 1.15^c^	< 0.001*
*E. cloaceae*	18.27 ± 0.25^a^	−	30.67 ± 0.70^b^	33.03 ± 2.27^b^	39.90 ± 0.78^c^	< 0.001*
*S. typhimurium*	19.47 ± 0.50^a^	−	31.33 ± 1.22^b^	30.50 ± 1.32^b^	33.23 ± 0.21^b^	< 0.001*
*P. aurantiogriseum*	−	17.43 ± 0.40^a^	35.17 ± 1.89^b^	34.33 ± 0.85^b^	58.57 ± 0.98^c^	< 0.001*
*C. albicans*	−	17.87 ± 0.81^a^	33.73 ± 0.70^b^	36.37 ± 1.87^b^	58.27 ± 0.46^c^	< 0.001*
*A. nivieus*	−	18.20 ± 0.35^a^	37.93 ± 1.10^b^	36.30 ± 0.30^b^	59.70 ± 0.44^c^	< 0.001*
*A. flavus*	−	17.90 ± 0.17^a^	35.83 ± 0.96 ^b^	36.03 ± 0.55^b^	58.77 ± 0.68^c^	< 0.001*
*A. fumigatus*	−	18.60 ± 0.26^a^	35.10 ± 2.01^b^	37.37 ± 0.40^b^	58.30 ± 0.52^c^	< 0.001*

Data are presented as Mean ± Standard deviation (SD).

*Statistically significant according to one-way ANOVA at *P* < 0.05.

^a–d^Different letters within the same row indicate statistically significant differences according to *post hoc* analysis by Tukey HSD at *p* < 0.05.

**TABLE 2:** Minimum inhibitory concentration (MIC) of TiO_2_NPs, CsNPs, and CNCs against *Sallmonella typhmurium* and *A. fumigatus.*

Nanoparticles concentration		*S. typhimurium*	*A. fumigatus*
TiO_2_NPs	5 μg/mL	+	+
	10 μg /mL	+	+
	20 μg /mL	−	−
	30 μg /mL	−	−
	40 μg /mL	−	−
	50 μg /mL	−	−
	70 μg /mL	−	−
	100 μg /mL	−	−
CsNPs	5 μg /mL	+	+
	10 μg /mL	+	+
	20 μg /mL	−	−
	30 μg /mL	−	−
	40 μg /mL	−	−
	50 μg /mL	−	−
	70 μg /mL	−	−
	100 μg /mL	−	−
CNCs	5 μg /mL	+	+
	10 μg /mL	−	−
	20 μg /mL	−	−
	30 μg /mL	−	−
	40 μg /mL	−	−
	50 μg /mL	−	−
	70 μg /mL	−	−
	100 μg /mL	−	−

−, No growth. +, positive growth.

**FIGURE 11:**
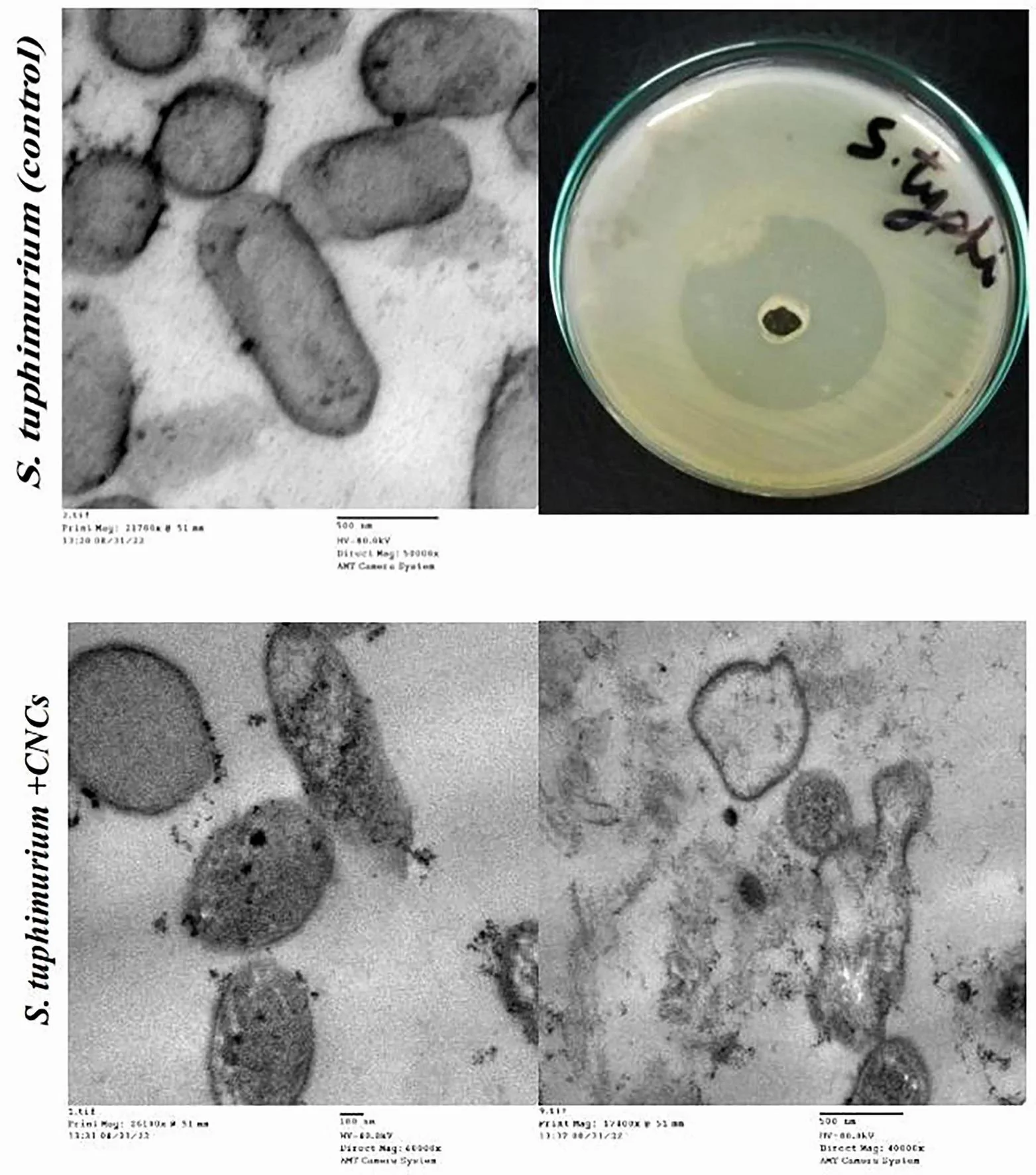
TEM of antibacterial activity of CNCs on *S. typhimurium.*

**FIGURE 12:**
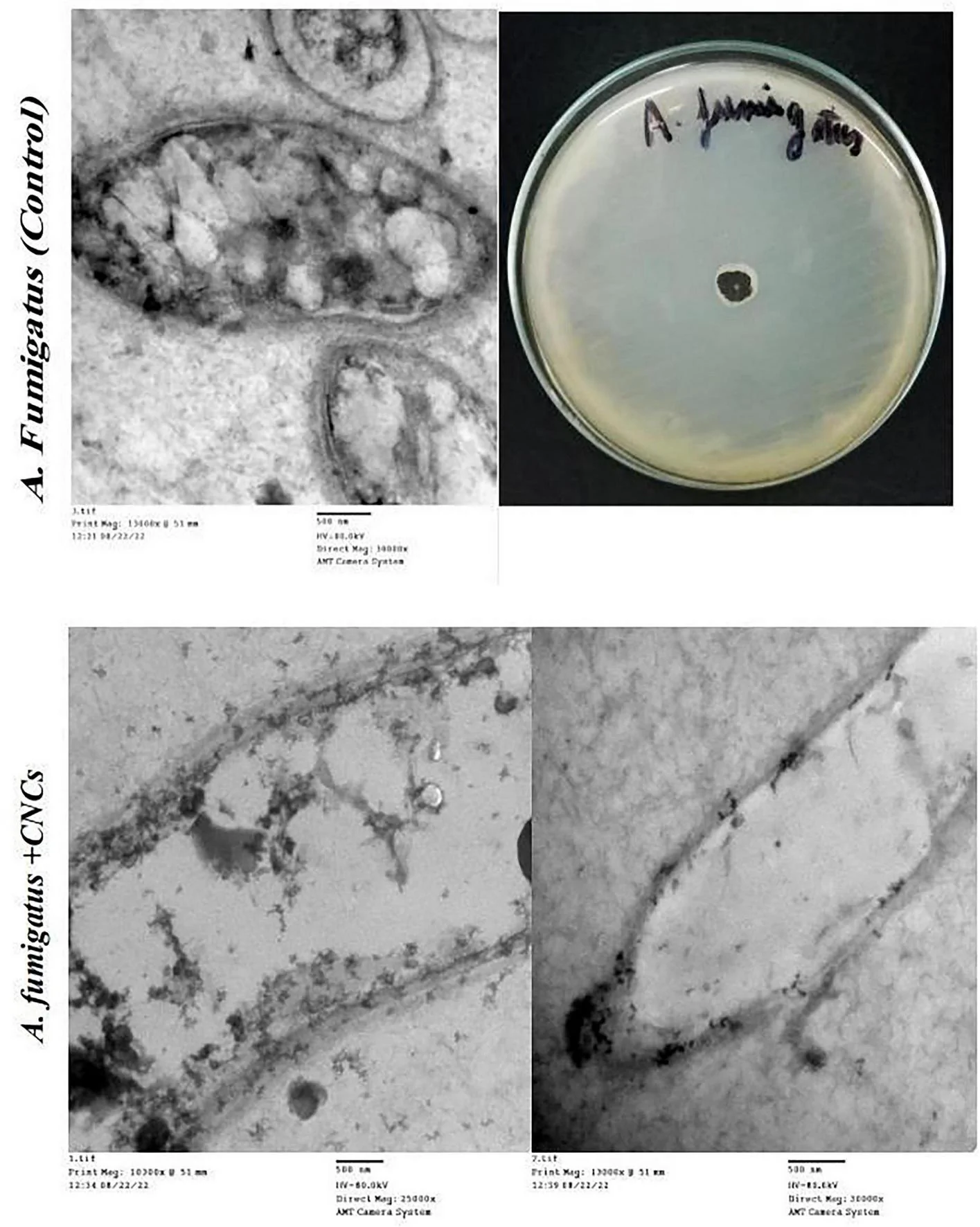
TEM of antifungal activity of CNCs on *A. fumigatus*

After incubation in nutrient broth at 37°C for 4 h, TEM images demonstrated the reducing effect of CNCs on the number of intact *S. typhimurium* cells (OD_600_ = 0.5 at the time of application) (Figure 11). Some bacterial cells exhibited various morphological deformations, including cytoplasmic emptiness, pore formation, membrane wrinkling, and cell shrinkage. TEM analysis indicated that the cationic proteins primarily affected the cell membrane and cell wall. Additionally, TEM micrographs of *A. fumigatus* cells treated with CNCs revealed cell shrinkage, disintegration of the cell wall, and loss of intracellular organization, indicating severe cellular damage (Figure 12).

### 3.7 MTT colorimetric and cytotoxicity assay of CNCs

To assess the probable cytotoxic impacts of varying concentrations of natural CNCs and their suitability for use, a cytotoxicity assay was performed on human normal melanocytes (HFB4). Preliminary results indicated that cells remained viable and stable up to 125 μg/mL, and CNC concentrations of up to 200 μg/mL were found to be safe and non-toxic. Cytotoxicity exhibited a concentration-dependent manner, with higher concentrations resulting in reduced cell vitality. The CC_50_ values were 200.54 ± 7.06 μg/mL for HFB4 (Supplementary Figure 8).

The MTT colorimetric assay was slightly modified to evaluate the growth of human colon cancer (HCT-116) and hepatocellular carcinoma (HepG2) cells in response to different CNC concentrations. CNCs inhibited the growth of HCT-116 and HepG2 cells, as indicated by stained cell images showing a decrease in cell density with increasing CNC concentrations (Supplementary Figure 8). Control HCT-116 and HepG2 cells exhibited polygonal and rounded morphologies, indicative of irregular, confluent aggregation (Figure 9). After 48 h of treatment with CNCs at 125 and 500 μg/mL, polygonal cells shrank and adopted a spherical shape (Figure 9). The inhibitory effect of CNCs on HCT-116 cells increased with increasing the concentration, at an IC_50_ of 81.20 ± 4.22 μg/mL. Similarly, CNCs inhibited HepG2 cell growth in a concentration-dependent pattern, with an IC_50_ of 93.1 ± 3.4 μg/mL (Supplementary Figure 8).

### 3.8 Cytotoxicity evaluation and determination of oxidative enzymes

#### 3.8.1 Antioxidant activity of CNCs

Using the ABTS radical scavenging assay, CNCs demonstrated significant antioxidant activity, with an IC_50_ of 9.98 ± 0.53 μg/mL compared to 3.52 ± 0.42 μg/mL for ascorbic acid. When applied in the ABTS system, which induces oxidative stress in tumor cells, CNCs exhibited limited antioxidant activity (Supplementary Figure 9).

#### 3.8.2 CDK-2 enzyme inhibition assay

The study further showed that CNCs could inhibit CDK-2 enzyme activity in HCT-116 cells, with roscovitine used as a reference standard at an IC_50_ of 11.41 μg/mL (Supplementary Figure 10).

### 3.9 Evaluation of cytotoxicity against the HCT-116 cell line with oxidative stress markers and ROS determination

The activities of several oxidative stress markers, involving CAT, SOD, GSH and lipid peroxidation (MDA), were evaluated in HCT-116 cells treated with CNCs (Table 3). Furthermore, the cytotoxic effect of CNCs was assessed by measuring total protein content. Compared to control cells, treatment with the IC_50_ concentration of CNCs elevated the levels of SOD, MDA, and GSH while simultaneously suppressing CAT. ROS levels were elevated relative to controls. These results indicate that CNCs induce cell death primarily by disrupting the equilibrium between ROS synthesis and the antioxidant defense system. Thus, CNCs exert their effect through enhanced ROS levels (Table 4).

**TABLE 3:** Effect of CNCs on oxidative enzymes by ELISA technique.

Sample code oxidative enzymes (nMol/mg protein)	CNCs	HCT-116 cell control (non-treated)
Catalase	6.21 ± 0.02	11.99 ± 0.33
Superoxide dismutase (SOD)	91.62 ± 2.31	54.33 ± 3.37
Glutathione (GSH)	5.19 ± 0.84	2.45 ± 0.27
Malonaldehyde (MDA)	3.31 ± 0.23	0.88 ± 0.05

**TABLE 4:** Evaluation of mechanism of cytotoxicity of CNCs against HCT-116 cell line, ROS Determination using ELISA.

Sample code	Tested conc. (μg/mL)	ROS (Pg/mL)	% of control
CNCs (Treated cells)	*IC_50_ = 81.2*	99.43 ± 3.69	118.03
HCT-116 cells (control)	0	84.24 ± 8.07	100

### 3.10 Molecular docking analysis and binding affinity assessment

Molecular docking simulations of chitosan-titanium nanoparticle complexes (CNCs) against three distinct therapeutic targets revealed significant binding interactions with varying affinities using a simplified ligand model and does not fully represent the structural complexity of CNCs. The calculated binding energies indicated that CNCs exhibited the strongest affinity for cyclin-dependent kinase 2 (CDK2) from human colon carcinoma, with a VINA score of −6.848 kcal/mol, followed by FabG-D from *Salmonella typhimurium* (−6.648 kcal/mol) and CYP51B from *Aspergillus fumigatus* (−6.428 kcal/mol). These binding energies fall within the range typically associated with moderate to strong protein-ligand interactions, suggesting potential therapeutic efficacy against multiple pathogenic targets (Table 5).

**TABLE 5:** Docking scores and hydrogen bonding of (CYP51B) from a pathogenic filamentous fungus *Aspergillus fumigatus* (PDB ID: 5FRB), FabG–D from *Salmonella typhimurium* (PDB ID: 6T7M) and cyclin dependent kinase 2 (CDK2) from human colon carcinoma (PDB ID: 2FVD).

Drug	Smiles			PubChem ID	Formula
Chitosan–Titanium (CNCs)	C([C@@H]1[C@H]([C@@H]([C@H]([C@@H](O1)O[C@@H]2[C@H] (OC([C@@H]([C@H]2O)N)O)CO)N)O)O)O)Ti			57,369,765	C_12_H_26_N_2_O_9_
Free binding energy of the ligands, temperature (T) = 298.15 K					
**Chitosan–titanium (CNCs)**					
5FRB—Crystal structure of (CYP51B)	VINA RESULTS:	−6.428			
	INTER + INTRA:	−11.014			
	INTER:	−9.622			
	INTRA:	−1.392			
	UNBOUND:	−1.392			
	12 active torsions				
	**Donor**	**Acceptor**	**Hydrogen**	**D. Å dist.**	**D-H. Å dist.**
	GLN 309 (NE2)	1 Ch-Ti (O)	GLN 309 (HE22)	3.053	2.127
	1 Ch-Ti (N)	SER 313 (OG)	1 Ch-Ti (H)	3.216	2.450
**Chitosan–titanium (CNCs)**					
6T7M—Crystal structure of Salmonella typhimurium FabG–D	VINA RESULTS:	−6.648			
	INTER + INTRA:	−11.388			
	INTER:	−9.951			
	INTRA:	−1.437			
	UNBOUND:	−1.437			
	12 active torsions				
	**Donor**	**Acceptor**	**Hydrogen**	**D. Å dist.**	**D-H. Å dist.**
	SER 138 (OG)	1 Ch-Ti (O)	SER 138 (HG)	3.293	2.364
	LYS 155 (NZ)	1 Ch-Ti (O)	LYS 155 (HZ1)	2.985	2.039
	1 Ch-Ti (N)	GLY 88 (O)	1 Ch-Ti (H)	3.341	2.368
	1 Ch-Ti (N)	GLY 182 (O)	1 Ch-Ti (H)	2.857	1.852
**Chitosan–titanium (CNCs)**					
2FVD - Cyclin Dependent Kinase 2 (CDK2) - human colon carcinoma	VINA RESULTS:	−6.848			
	INTER + INTRA:	−11.759			
	INTER:	−10.252			
	INTRA:	−1.508			
	UNBOUND:	−1.508			
	12 active torsions				
	**Donor**	**Acceptor**	**Hydrogen**	**D. Å dist.**	**D-H. Å dist.**
	1 Ch-Ti (N)	LEU 83 (O)	1 Ch-Ti (H)	2.984	1.957
	1 Ch-Ti (N)	GLN 131 (O)	1 Ch-Ti (H)	3.113	2.438
	1 Ch-Ti (O)	ASP 86.A OD2	1 Ch-Ti (H)	2.735	1.774

#### 3.10.1 Antifungal mechanism: CYP51B inhibition in *Aspergillus fumigatus*

**FIGURE 13:**
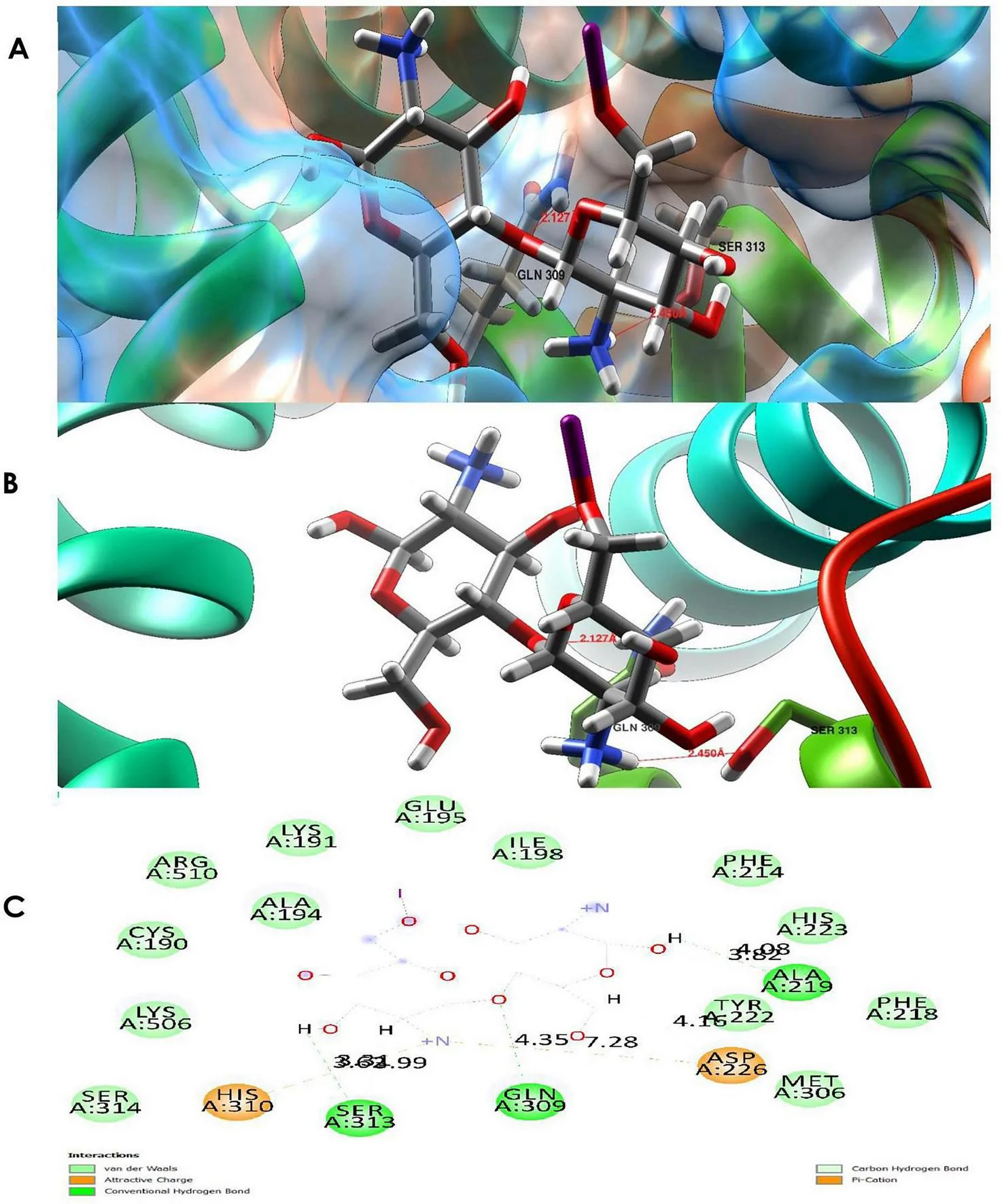
Molecular docking visualization of CNCs complex bound to CYP51B from *Aspergillus fumigatus* (PDB ID: 5FRB). **(A,B)** The ligand positioned within the active site with key hydrogen bonding interactions highlighted. The protein secondary structure is represented in cartoon format with the ligand displayed in stick representation. Detailed interaction diagram showing CNCs docking within the CYP51B active site, **(C)** the figure displays amino acid residues within the binding pocket, hydrogen bonding distances, and the spatial orientation of the titanium-chitosan complex relative to key catalytic residues.

Docking analysis of CNCs against CYP51B revealed a binding energy of −6.428 kcal/mol, with detailed energy contributions including intermolecular interactions (−9.622 kcal/mol) and intramolecular strain (−1.392 kcal/mol). Binding mode analysis identified two key hydrogen bonds stabilizing the ligand within the enzyme’s active site: GLN 309 (NE2) formed a hydrogen bond with the oxygen atom of CNCs at a distance of 3.053 Å, and the nitrogen atom of CNCs established a hydrogen bond with SER 313 (OG) at 3.216 Å. CYP51B is a critical enzyme in the ergosterol biosynthesis pathway of fungi, catalyzing the 14α-demethylation of lanosterol (Figure 13).

#### 3.10.2 Antibacterial mechanism: FabG-D targeting in *Salmonella typhimurium*

**FIGURE 14:**
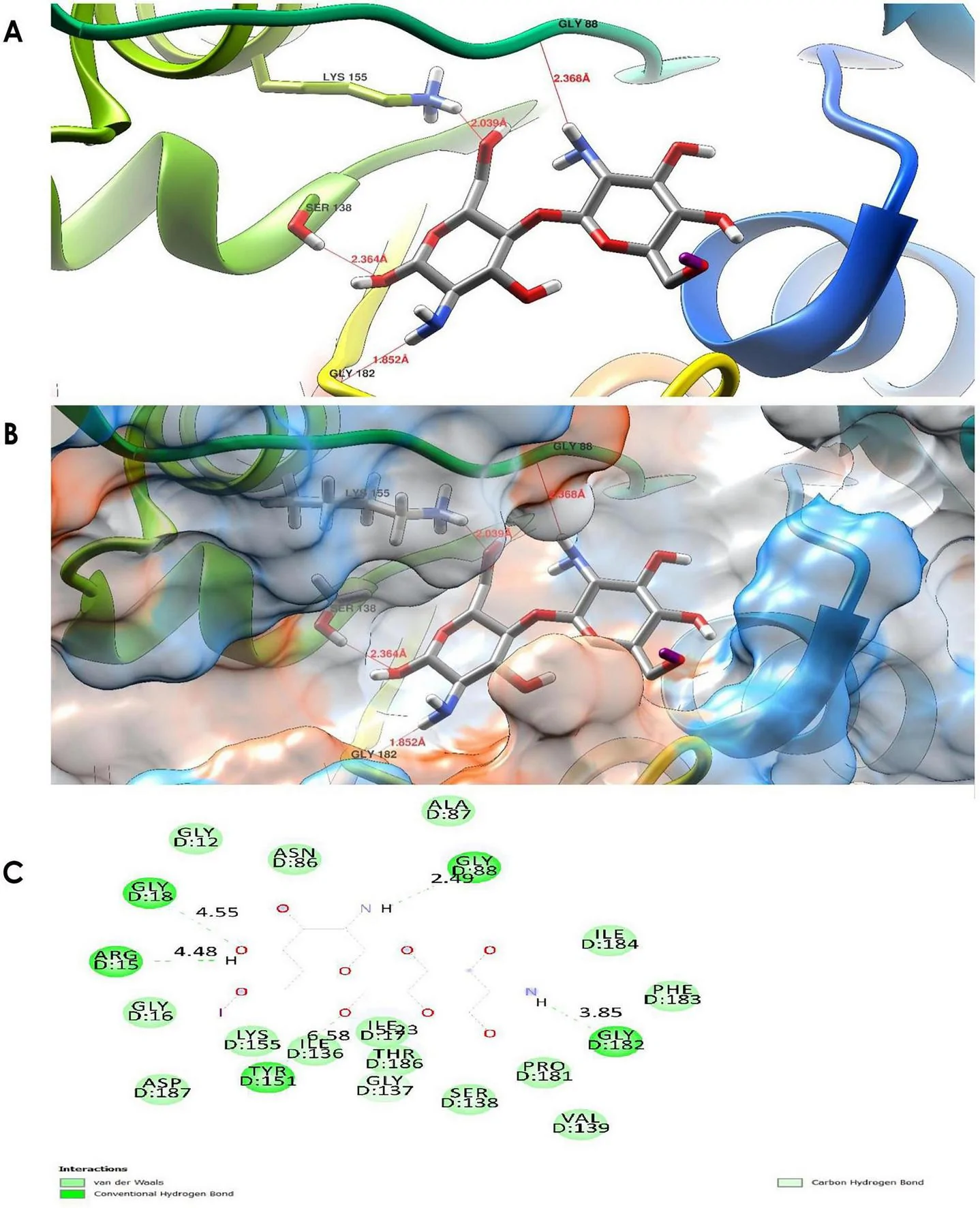
Molecular surface representation of FabG-D with CNCs bound in the active site. **(A,B)** The figure illustrates the complementarity between the ligand shape and the enzyme’s binding pocket, **(C)** hydrogen bonding networks clearly marked and labeled.

The interaction between CNCs and FabG-D from *Salmonella typhimurium* exhibited a higher binding affinity (−6.648 kcal/mol) compared to the fungal target, with intermolecular energy contributions of −9.951 kcal/mol. Binding stabilization involved four distinct hydrogen bonds, indicating an extensive network of molecular interactions. SER 138 (OG) and LYS 155 (NZ) acted as hydrogen bond donors to oxygen atoms of CNCs at distances of 3.293 Å and 2.985 Å, respectively. Additionally, the nitrogen atoms of CNCs formed hydrogen bonds with the backbone oxygen atoms of GLY 88 and GLY 182 at distances of 3.341 Å and 2.857 Å. FabG-D serves as a 3-oxoacyl-ACP reductase in bacterial fatty acid biosynthesis, catalyzing the NADPH-dependent reduction of β-ketoacyl-ACP intermediates (Figure 14).

#### 3.10.3 Anticancer mechanism: CDK2 inhibition in human colon carcinoma

**FIGURE 15:**
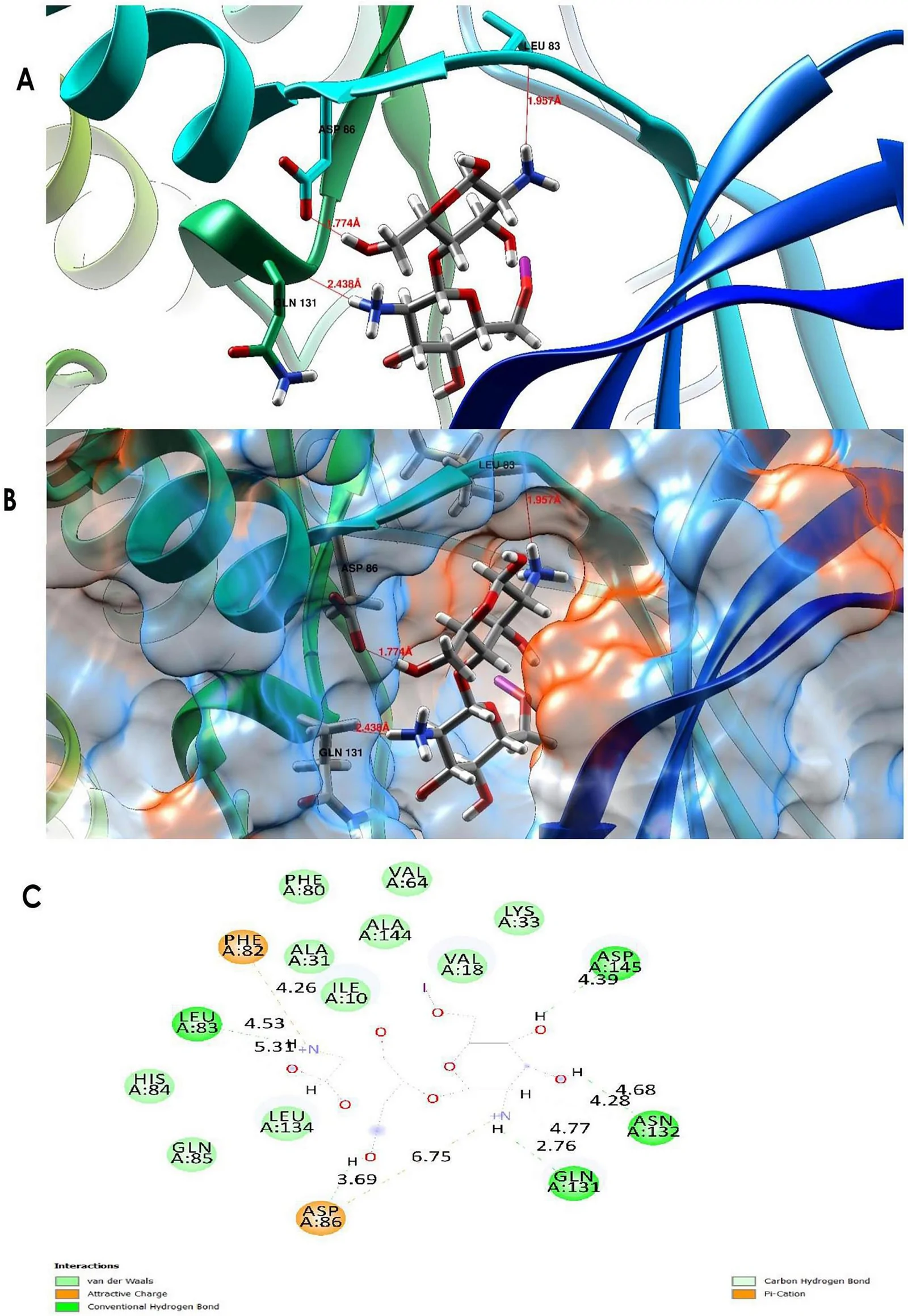
CDK2-CNCs complex showing the ligand positioned within the ATP-binding site. **(A,B)** The figure demonstrates the steric hindrance created by the titanium-chitosan complex that prevents ATP binding**, (C)** key residues involved in catalytic activity clearly labeled.

The most favorable binding interaction was observed between CNCs and CDK2, with a binding energy of −6.848 kcal/mol and the highest intermolecular interaction energy (−10.252 kcal/mol) among the three targets. Binding stabilization involved three strategically positioned hydrogen bonds: the nitrogen atoms of CNCs interacted with LEU 83 (O) and GLN 131 (O) at distances of 2.984 Å and 3.113 Å, respectively, while an oxygen atom of CNCs formed a hydrogen bond with ASP 86.A (OD2) at 2.735 Å. CDK2 plays a pivotal role in cell cycle regulation, specifically controlling the G1/S and intra-S phase transitions through phosphorylation of key substrate proteins (Figure 15).

The differential binding affinities observed among the three targets can be attributed to variations in active site architecture and the nature of stabilizing interactions. The progressively higher binding affinity from CYP51B to FabG-D to CDK2 correlates with the number and strength of hydrogen bonds, ranging from two to four interactions. The titanium center in the CNCs complex provides additional coordination sites that enhance binding specificity, while the chitosan backbone offers multiple hydroxyl and amino groups capable of forming hydrogen bonds, contributing to overall binding stability.

## 4 Discussion

Identifying novel strategies to suppress and inhibit microbial resistance to antibiotics is of critical importance (Abdel-Shafi et al., 2020; El-Gazzar et al., 2025). Nanotechnology introduces effective methods for inhibiting harmful multidrug-resistant microorganisms (El-Gazzar and Ismail, 2020). Over the last decade, nanotechnology has emerged as a novel biotechnology for the development of drug-based nanoparticles (El-Gazzar and Enan, 2020; El-Gazzar et al., 2024). Recently published findings indicated that fungal strains can biosynthesize nanoparticles (El-Gazzar et al., 2024).

Similar to previous findings, the biosynthesized TiO_2_NPs in this study demonstrated an appropriate size of approximately 40.7 nm, as determined by DLS, and excellent stability (> ± 30 mV), as indicated by zeta potential (El-Gazzar and Ismail, 2020). According to El-Gazzar and Ismail (2020), the solitary peak that was observed indicated that the biosynthesized nanoparticles are of satisfactory quality. TiO_2_NPs were dispersed in round and oval shapes. Furthermore, the outcomes established the existence of several molecules responsible for the synthesis and stabilization of TiO_2_NPs, which is consistent with earlier findings in this field (El-Gazzar et al., 2020).

The observed temporal stability validates the corrected zeta potential measurements and confirms robust colloidal stabilization through electrostatic repulsion mechanisms. The minimal size growth (< 8% over 30 days) indicates that attractive van der Waals forces are effectively counterbalanced by repulsive electrostatic interactions. The gradual zeta potential decline likely reflects slow surface equilibration processes (counterion adsorption, surface hydroxyl rearrangement) rather than fundamental stability loss. The sustained maintenance of |ζ| near or above 20 mV throughout the 30-day period provides definitive evidence of long-term colloidal integrity suitable for practical antimicrobial and photocatalytic applications requiring extended shelf-life stability. The comprehensive PDI analysis demonstrates that all nanoparticle systems exhibit excellent monodispersity, validating synthesis protocol optimization and confirming colloidal stability through both size distribution metrics and zeta potential measurements. The stability and capacity to reduce or oxidize metals are enhanced by the presence of specific biomolecules associated with nanoparticles (El-Gazzar et al., 2024).

Additionally, TiO_2_NPs and CsNPs of various forms were dispersed without agglomeration, as shown by TEM micrographs, and were evenly distributed throughout the solution. This may be attributed to the multiple chemicals in the fungal product, which likely act as agents preventing agglomeration and capping (El-Gazzar et al., 2023). Furthermore, a previous study employed DLS and TEM analyses to demonstrate that the synthesized TiO_2_NPs are spherical in form and range in size from 17 to 35 nm (El-Gazzar et al., 2024). In the present study, CsNPs were combined with biosynthesized TiO_2_NPs to form a new nanocomposite, which was expected to exhibit increased bioactivity. The entrapment efficiency showed a clear correlation with chitosan concentration up to 1.5% w/v, after which a plateau effect was observed. The maximum EE% of 86.7 ± 1.8% was achieved at 1.5% w/v chitosan concentration. This can be attributed to the increased viscosity of the chitosan solution, which provided better structural integrity, enhanced crosslinking density leading to improved TiO_2_ retention, and an optimal polymer-to-nanoparticle ratio for stable complex formation.

As stated in the earlier study, XRD examination revealed that the new nanocomposite exhibited peaks corresponding to TiO_2_ NPs and a characteristic cubic lattice (El-Gazzar et al., 2025). Furthermore, an earlier study by Sellami et al. (2025), demonstrated that the biosynthesized TiO_2_NPs are crystalline in nature, which is consistent with our findings. The CNCs investigated herein physicochemical features with these established nanocarrier systems, including nanoscale dimensions, polymeric composition, and an electrostatic interaction capability. In this context, (Al-Fawares et al., 2025) reported the development of chitosan–polyacrylic acid complex nanoparticles as nanocarrier systems that have been successfully developed for oral delivery applications and biologically relevant nanocarriers.

The comprehensive optimization studies reveal complex interplay between electrostatic drug-polymer interactions, physical entrapment within cross linked networks, and formulation processing parameters governing ultimate drug loading performance. Ciprofloxacin incorporation into chitosan-TiO_2_ nanocomposites proceeds through dual retention mechanisms operating synergistically: (1) Electrostatic Complexation at pH 4.5, ciprofloxacin exists predominantly as zwitterionic species with deprotonated carboxylate group (pKa_1_ ∼6.1) electrostatically attracted to chitosan’s abundant protonated amines (-NH_3_^+^), forming ionic drug-polymer complexes with dissociation constants (Kd) in low micromolar range providing strong binding affinity; (2) Physical Entrapment during ionic gelation, STPP polyphosphate anions rapidly crosslink chitosan chains creating three-dimensional network with nanoscale mesh size that physically entraps drug molecules regardless of electrostatic state. The relative contribution of each mechanism varies with formulation conditions: electrostatic binding dominates at acidic pH (4.0–5.5) where both drug and polymer carry appropriate charges, while physical entrapment becomes increasingly important at near-neutral pH where reduced chitosan protonation diminishes electrostatic interactions. This dual-mechanism paradigm explains observed pH-dependent entrapment efficiency maximum at pH 4.5 where electrostatic binding is strongest, declining at both lower pH (drug protonation creating electrostatic repulsion) and higher pH (polymer deprotonation reducing binding sites) (El-Sherbiny et al., 2024).

Chitosan concentration emerged as critical determinant through multiple mechanisms: (1) Binding Site Availability amine group density scales linearly with polymer concentration (24 mM at 0.5% → 120 mM at 2.5%), directly increasing electrostatic drug binding capacity; (2) Network Density higher chitosan concentration promotes denser crosslinking (more chitosan-STPP associations per unit volume), reducing mesh size (ξ) from > 50 nm (0.5% chitosan) to < 15 nm (2.0% chitosan), restricting drug diffusion during purification; (3) Solution Viscosity elevated polymer concentration increases viscosity exponentially (η∝ c^3^⋅^4^ for entangled polymer solutions beyond overlap concentration c*), eventually impeding uniform drug distribution at excessive concentrations (> 2.0%). The biphasic entrapment efficiency behavior (increasing 0.5–1.75%, declining 1.75–2.5%) reflects competing optimization: network densification enhances retention but excessive viscosity compromises mixing efficiency. Similarly, drug: polymer ratio optimization balances absolute payload against binding site saturation low ratios achieve high efficiency but insufficient loading, high ratios provide greater payload but declining efficiency as chitosan binding capacity saturates. The identified optimal conditions (1.5% chitosan, 4% ratio) represent practical compromise maximizing both metrics while maintaining processability and colloidal stability (Elshayb et al., 2022).

The pronounced pH-dependent release behavior originates from chitosan’s pH-sensitive swelling characteristics arising from its weak polyelectrolyte nature. Chitosan contains primary amine groups (pKa ∼6.5) undergoing pH-dependent protonation equilibrium: at acidic pH, extensive protonation generates polycationic chains with strong intramolecular and intermolecular electrostatic repulsion, causing network swelling, mesh size enlargement, and enhanced drug diffusivity; at neutral-basic pH, deprotonation yields uncharged polymer chains with collapsed conformation, minimal swelling, tight network structure, and restricted drug diffusion. Quantitatively, swelling ratio (SR = mass wet/mass dry) increases from ∼180% at pH 7.4 to ∼450% at pH 1.2, corresponding to ∼2.5-fold mesh size enlargement based on Flory-Rehner swelling theory, directly translating to ∼6-fold diffusivity enhancement (D ∝ξ^2^ assuming free volume theory). Additionally, drug-polymer electrostatic binding strength exhibits pH-dependence: at acidic pH, ciprofloxacin carboxyl protonation converts zwitterionic drug (bound to chitosan) to cationic form (repelled by chitosan), accelerating dissociation; at physiological pH, maintained zwitterionic character preserves electrostatic complexation, retarding release (Katowah and Ismail, 2025).

This pH-responsive behavior offers strategic therapeutic advantages for infection-targeted delivery. Bacterial infection sites characteristically exhibit acidic microenvironments (pH 5.5–6.5) generated through bacterial metabolic acid production (lactic acid from glycolysis, acetic acid from fermentation), host inflammatory response (macrophage activation releasing acidic mediators), and tissue ischemia-induced metabolic acidosis. The demonstrated accelerated release at pH 5.5 (approaching complete release ∼98% vs. ∼94% at pH 7.4) provides infection site-selective drug delivery: minimal premature release in healthy physiological tissues (pH 7.4), triggered burst release upon nanoparticle accumulation at acidic infection foci, maximizing local antimicrobial concentrations while minimizing systemic exposure and associated toxicity. This passive targeting mechanism complements active targeting strategies (e.g., bacterial surface ligand-conjugated nanoparticles) and enhances therapeutic index. Furthermore, combination with TiO_2_ photocatalytic ROS generation (requiring UV activation) enables dual-stimuli responsive systems: pH-triggered ciprofloxacin release provides baseline antimicrobial activity, with UV-switchable photocatalytic oxidative damage offering on-demand enhancement for severe/recalcitrant infections. The triple-mechanism antimicrobial approach (ciprofloxacin DNA replication inhibition + chitosan membrane disruption + TiO_2_ ROS generation) reduces single-mechanism resistance development probability, addressing critical antibiotic resistance challenges in contemporary infectious disease management (Mahboub et al., 2025a).

Korsmeyer-Peppas modeling identified non-Fickian transport (*n* = 0.693, 0.45 < *n* < 0.89) as the governing release mechanism, indicating drug liberation controlled by coupled diffusion and polymer relaxation rather than pure concentration gradient-driven Fickian diffusion. Mechanistically, drug release from swelling-controlled hydrogel systems involves three sequential processes: (1) Water penetration into glassy polymer creating swollen gel layer advancing inward as “swelling front”; (2) Drug dissolution within swollen gel; (3) Drug diffusion outward through swollen gel to bulk medium. When water penetration rate substantially exceeds drug diffusion rate (glassy polymers, high molecular weight drugs), Case-II transport emerges with zero-order release kinetics (*n* = 1.0). When drug diffusion substantially exceeds swelling, classical Fickian diffusion governs (*n* ≤ 0.45) with t^∧^0.5 kinetics. Intermediate n values (0.45–0.89) indicate comparable timescales for swelling and diffusion, yielding anomalous transport. The observed *n* = 0.693 suggests chitosan network swelling and ciprofloxacin diffusion proceed at similar rates, with neither process clearly dominating. This interpretation aligns with chitosan’s semi-crystalline structure (crystalline domains requiring prolonged hydration versus rapidly-swelling amorphous regions) and ciprofloxacin’s moderate molecular weight (MW 331.3 Da, Stokes radius ∼0.5 nm), yielding intermediate diffusivity through nanoscale network mesh (Alshubramy et al., 2025).

From clinical translation perspective, the demonstrated release profile (72.5% at 24 h, 93.7% at 96 h at pH 7.4) provides sustained antimicrobial action suitable for once-daily or twice-daily dosing regimens, improving patient compliance versus conventional immediate-release ciprofloxacin tablets requiring thrice-daily administration. The initial burst phase (28.3% release by 4 h) provides rapid achievement of therapeutic concentrations (typical ciprofloxacin MIC: 0.125–2 μg/mL for susceptible pathogens), while sustained phase maintains concentrations above MIC for extended durations reducing post-antibiotic sub-MIC exposure periods implicated in resistance development. Loading capacity of 5.9% (59 mg/g nanocomposite) enables practical dosing: achieving 500 mg ciprofloxacin dose (standard oral tablet strength) requires ∼8.5 g nanocomposite, feasible for topical wound dressing applications (typical dressing: 10 × 10 cm loaded at 100 mg/cm^2^ provides adequate payload). For systemic delivery, further optimization increasing LE% to 15–20% would improve feasibility, achievable through higher drug:polymer ratios (6–8%), though potentially compromising entrapment efficiency, or alternative loading methodologies (supercritical CO_2_ impregnation, electrospraying). The UV-triggered enhancement (23% increase) offers clinical utility in photodynamic antimicrobial chemotherapy combining light-activated ROS generation with antibiotic release, particularly valuable for superficial/accessible infections (skin wounds, oral/oropharyngeal infections, catheter-associated infections) amenable to external UV irradiation. Safety considerations require UVA (365 nm) rather than UVB/UVC to minimize DNA damage risk, with controlled fluence rates (< 50 mW/cm^2^) preventing thermal tissue injury (Ibrahim et al., 2025).

The developed ciprofloxacin-loaded chitosan-TiO_2_ nanocomposites demonstrate superior performance compared to conventional chitosan-only or TiO_2_-only drug delivery systems reported in literature. Pure chitosan nanoparticles typically achieve 60–75% entrapment efficiency with ciprofloxacin, substantially lower than our optimized 86.7%, attributed to dual immobilization (electrostatic + physical) and TiO_2_ presence potentially enhancing crosslinking density through chitosan-TiO_2_ interfacial hydrogen bonding. Loading capacities of 3–5% are typical for chitosan systems, comparable to our 5.9%, though higher values (10–15%) reported for alternative polymers (PLGA, PCL) requiring organic solvent-based synthesis incompatible with hydrophilic drugs like ciprofloxacin. The pH-responsive release magnitude (97.8% at pH 1.2 vs. 93.7% at pH 7.4, ∼4% difference) appears modest compared to some reports (> 20% difference), likely reflecting our robust crosslinking (60-min STPP treatment) creating more pH-stable networks, though sufficient for infection site targeting. UV-triggered enhancement (23%) aligns with photocatalytic polymer degradation mechanisms reported for TiO_2_-polymer composites, with our magnitude comparable to or exceeding literature values (10–30% typical range). Critically, the triple-mechanism antimicrobial approach (ciprofloxacin + chitosan + TiO_2_) offers unique advantages over single-agent systems: *in vitro* antimicrobial studies would be expected to demonstrate synergistic effects (fractional inhibitory concentration index FIC < 0.5) against both Gram-positive and Gram-negative pathogens, substantially exceeding individual component activities. Clinical positioning targets applications where conventional antibiotics face challenges: chronic wounds (diabetic ulcers, pressure sores) exhibiting biofilm-mediated antibiotic resistance responsive to multi-mechanistic attack; burn infections requiring localized high-dose antimicrobial delivery avoiding systemic toxicity; catheter-associated infections amenable to device coating strategies; and emerging as next-generation antimicrobial wound dressings integrating controlled drug release with intrinsic antibacterial properties (Gad et al., 2022).

Several limitations warrant acknowledgment: (1) *In vitro* release studies employed simplified buffer systems lacking biological complexity (proteins, enzymes, cells) potentially influencing *in vivo* performance; future work should incorporate serum-containing media, enzymatic degradation studies (lysozyme-mediated chitosan hydrolysis), and cellular uptake/cytotoxicity assessment; (2) Antimicrobial efficacy validation remains incomplete comprehensive bactericidal kinetics studies against clinically-relevant strains (including antibiotic-resistant isolates: MRSA, extended-spectrum β-lactamase [ESBL]-producing Enterobacteriaceae, multidrug-resistant Pseudomonas aeruginosa), biofilm eradication testing, and synergy quantification (checkerboard assays, time-kill studies) are essential for clinical translation; (3) *In vivo* pharmacokinetics, biodistribution, and efficacy in animal infection models (murine wound infection, porcine burn model) required to establish therapeutic proof-of-concept; (4) Safety evaluation acute/chronic cytotoxicity, genotoxicity, and inflammatory response assessment necessary for regulatory approval pathways; (5) Stability studies long-term storage stability (6–24 months) under various conditions (temperature, humidity, light exposure) and accelerated degradation testing according to ICH guidelines needed for commercial viability; (6) Scale-up considerations current synthesis yields ∼50–100 mg batches; manufacturing scale-up to gram/kilogram quantities while maintaining batch-to-batch consistency requires process optimization and quality control protocols; (7) Regulatory pathway clarity nanocomposite classification (drug, device, or combination product) influences approval requirements necessitating early regulatory consultation. Future directions include: optimization for specific infection types (Gram-positive vs. Gram-negative tailored formulations), incorporation of additional antimicrobials (dual-drug loading for enhanced spectrum/synergy), biofilm-penetrating modifications (D-amino acids, dispersin B), targeting ligand conjugation (bacterial lectin-binding molecules), and combination with advanced wound care technologies (electrospun nanofiber mats, hydrogel composites, 3D-printed scaffolds) creating next-generation multifunctional infection management platforms (Abdel-Hamied et al., 2022; Katowah and Ismail, 2025). The therapeutic effectiveness of nanoparticles has been well-documented in the literature (El-Gazzar and Enan, 2020; El-Gazzar et al., 2025). Numerous investigations have emphasized that the small diameter of metal-oxide nanoparticles, which enables them to penetrate microbial cell membranes and cause toxicity and cell destruction, gives them genuine antibacterial activity (El-Gazzar et al., 2024). TiO_2_NPs and hybrid chitosan nanocomposites were tested for their anticancer and antimicrobial properties against harmful bacterial and fungal species. Previous studies have reinforced the concept that metal-oxide nanoparticles play a crucial role as antimicrobial agents (El-Gazzar et al., 2025).

The antimicrobial activity of CNCs against *S. typhimurium* and *A. fumigatus* was found to be substantially greater than that of TiO_2_NPs or CsNPs alone in the current investigation. This finding is in line with that of Sitohy et al. (2021), who illustrated that combining two drugs produces a positive interaction and that their combined inhibitory effect is greater than the sum of their individual effects. Additionally, a previous study showed that drug-resistant *Candida albicans* may be successfully treated by combining zinc nanoparticles with probiotics (El-Gazzar et al., 2024). Moreover, prior research has reported that TiO_2_NPs, either alone or in combination with other antibiotics, are effective against *P. aeruginosa* (Haji et al., 2024). TiO_2_NPs exert a synergistic effect with antibiotics because their large surface area and nanosize facilitate metabolite release by enabling the more straightforward incorporation and delivery of antibiotic components into cells, as well as their spread across cell walls and transfer channels (Abou Elez et al., 2021). Moreover, prior research demonstrated that *E. coli* cells were disrupted by silver-loaded TiO_2_ (Caballero et al., 2009; Hajjaji et al., 2018). The current study demonstrated that TiO_2_NPs exhibit a straightforward binding mechanism and can readily penetrate microbial structures, including cell walls. Moreover, their effectiveness in promoting *A. fumigatus* morphogenesis aligns with the results of an earlier investigation (Yang et al., 2014).

Furthermore, earlier studies have shown that the positive charge of chitosan enables effective binding to cells, thereby elevating the likelihood of cellular uptake (Aibani et al., 2021). CNCs containing positively charged amino acid moieties exhibited electrostatic characteristics and whole formation within cell walls, leading to electrolyte loss and ultimately cell lysis (Aibani et al., 2021). They can permeate cell membranes, interact with specific molecules, or disrupt the formation of essential components such as glycan, chitin, or cell walls. The negatively charged components of microbial cell membranes, including lipopolysaccharides and lipoteichoic acid, are degraded and depolarized due to the cationic amino acid residues and their ability to form dimers. Moreover, the cell membrane, composed of proteins and lipids, serves as an efficient barrier against most substances. For therapeutic efficacy, drug molecules must be able to cross this barrier (Yang and Hinner, 2015). Phospholipids with negatively charged head groups are responsible for the net negative charge in mammalian plasma membranes (Magalhaes and Glogauer, 2010). Consequently, a cationic polymer such as chitosan can readily adhere to cell membrane surfaces through electrostatic interactions, thereby enhancing cellular absorption and inhibiting the growth of pathogenic microorganisms (Aibani et al., 2021).

As a result, the antimicrobial features of the nanocomposite were enhanced. The findings in this study confirmed that *S. typhimurium* is a multidrug-resistant bacterium; other authors have demonstrated that this strain is resistant to the effects of various drugs (Abou Elez et al., 2021). The inhibition of such MDR *S. typhimurium* by TiO_2_NPs is noteworthy. Biologically produced TiO_2_NPs were evaluated for their antimicrobial properties and were found to be highly effective against all tested species, with suppression of *S. typhimurium* at a dose of 20 μg/mL. Moreover, the synergy of CsNPs and TiO_2_NPs demonstrated a more potent suppression of MDR *S. typhimurium* than that achieved by CsNPs or TiO_2_NPs alone. Chitosan and titanium were distinguished by their superior effectiveness against microbes. Other research has suggested that TiO_2_NPs affect cell membrane permeability, thereby disrupting the bacterial respiratory chain; however, the precise underlying mechanisms remain unclear. Furthermore, they are linked to the creation of reactive oxygen species, like hydrogen peroxide, and the subsequent oxidative stress and cell damage (Haji et al., 2024). In this regard, our findings were contextualized relative to previously reported chitosan nanoparticle systems with documented antibiofilm activity. Notably, Al-Fawares et al. (2024) reported pronounced antimicrobial and antibiofilm effects of chitosan–polyacrylic acid nanoparticles versus pathogenic bacteria, emphasizing the importance of nanoparticle size, surface charge, and polymeric composition in disrupting biofilm architecture. These parameters are broadly consistent with the physicochemical characteristics of the nanomaterial investigated in the present study. The antimicrobial activity observed here is in agreement with proposed mechanisms for chitosan-based nanomaterials, including electrostatic interactions with negatively charged bacterial membranes, increased membrane permeability, and subsequent loss of cellular integrity.

The current work highlighted that CNCs products can effectively function as a sequential source for the separation of novel antiproliferative cancer medicines, which is in line with prior research findings (Jiang et al., 2019). CNCs clarified *in vitro* anticancer efficacy against the HCT-116 and HepG2 cell lines, as shown by the MTT assay. CNCs enhanced cellular uptake, accelerated internalization, and improved cell line adherence to hyaluronic acid-chitosan films (Yue et al., 2011; Neto et al., 2019). Additionally, this assay demonstrated that cytotoxicity was significantly enhanced when HCT-116 and HepG2 cells were treated with CNCs, a finding comparable to the antimicrobial testing results. Further, the antioxidant and antimicrobial effects observed herein in CNCs are mediated by surface interactions, nanoscale effects, and polymer-related redox modulation, this agreement with that reported by Barhoumi et al. (2024) about phytochemical extracts that investigate potent bioactivity due to their chemical complexity, nanomaterial-based systems offer complementary merits as effective stability and multifunctionality. Thus, our research demonstrated that TiO_2_NPs and CsNPs work synergistically to produce antibacterial and anticancer effects. This information can be used to develop a new class of antimicrobial and antitumor drugs.

In agreement with previous findings, this study demonstrated that CNCs have biological effects on oxidative stress biomarkers, including MDA, CAT, and GSH enzymes, which influenced HCT-116 cells (Aibani et al., 2021; Prasathkumar et al., 2022). Compared to the control, the amount of CAT that catabolizes ROS was lower in HCT-116 cells treated with CNCs. This ensures that ROS accumulate and induce tumor cell death by protein oxidation. These results support the idea that this treatment attempts to generate ROS to carry out its cytotoxic actions (Magalhaes and Glogauer., 2010). According to these findings, the treated cells contained less protein than the control because of oxidative damage caused by the overproduction of ROS (Eldehna et al., 2018).

Furthermore, ROS are naturally occurring byproducts of cellular metabolism, against which antioxidant enzymes protect cells. Rahman et al. (2022) speculated that the increased activity of these enzymes may be because of the increased incidence of tumors. Lipid peroxidation and free radicals are known to induce and promote carcinogenesis. Lipid peroxidation produces malondialdehyde (MDA), a byproduct that has been demonstrated to be mutagenic and carcinogenic. During the carcinogenic process, lipid peroxidation is increased (Ahmad et al., 2019).

CAT breaks down H_2_O_2_ into O_2_ and H_2_O. Additionally, SOD, which is present in various organs and tissues, is believed to protect cells from the damaging effects of superoxide radicals (Popovici et al., 2021). The superoxide radical O_2_^–^ is generated when the electron transfer oxidase system induces biological oxygen reduction. SOD catalyzes the one-electron reduction of oxygen to the superoxide radical. SOD reduces toxicity by preventing oxygen from producing free radicals (Popovici et al., 2021; Reda et al., 2022).

Furthermore, the detoxification of ROS, elimination of H_2_O_2_, translocation of amino acids across cell membranes, neutralization of lipid peroxide free radicals, and preservation of the thiol state of proteins are all components of the widely recognized antioxidant defense system of cells, or GSH. By directly reacting with ROS, CNCs exerted a protective effect against cell growth inhibition, as demonstrated by the increase in CNC therapy in this study and the depletion of GSH status in cancer control cells. CNCs enhanced glutathione activity, clearly demonstrating their protective role against oxidative damage. The findings unequivocally demonstrate that CNCs prevent lipid peroxidation and improve antioxidant status. Therefore, based on the current results, CNCs may serve as an effective chemotherapeutic medication.

In this regard, a noteworthy finding of the molecular docking analysis presented herein indicated that the binding mechanism is primarily driven by hydrophobic and van der Waals forces. Given the inherent structural and dynamic complexity of CNC nanocapsules, the use of a simplified ligand in the docking study represents a necessary approximation. As such, the docking results are intended to provide preliminary, qualitative insights into potential interaction trends at the molecular level and should be interpreted as exploratory and hypothesis-generating. It revealed distinct mechanisms of action for CNCs interactions across all three protein-ligand complexes, with no hydrogen bonding interactions observed. In the present study, molecular docking simulations of CNCs against three distinct therapeutic targets revealed significant binding interactions with varying degrees of affinity. These binding energies fall within the range typically associated with moderate to strong protein-ligand interactions, suggesting therapeutic efficacy across multiple pathogenic targets. The presence of the titanium center in the CNCs complex appears to provide additional coordination opportunities that enhance binding specificity. Furthermore, the chitosan backbone offers multiple hydroxyl and amino groups that can participate in hydrogen bonding, contributing to the overall binding stability. The theoretical mechanism of action involves CNCs binding to the ATP-binding pocket of CDK2, competitively inhibiting ATP binding and subsequent kinase activity. This inhibition prevents the phosphorylation of downstream targets such as retinoblastoma protein (pRb), histone H1, and *LEA*. In addition, CNCs binding disrupts the enzyme’s catalytic function by occupying the substrate binding site, thereby inhibiting ergosterol synthesis and compromising fungal cell membrane integrity, disrupting bacterial cell wall synthesis and simultaneously disrupt essential metabolic pathways in pathogens while targeting cancer cell proliferation mechanisms in human cells. Further experimental validation and advanced multiscale modeling approaches are required to more accurately to establish this hypothesis and capture the behavior of the full nanocapsule system.

The findings of this work have once again demonstrated the importance of CsNPs and TiO_2_NPs in combination as potential antimicrobial agents. According to this study, biogenic TiO_2_NPs are not only potent antimicrobial agents but also reasonably priced. Although both TiO_2_NPs and CsNPs showed strong antibacterial activity, a combination of both at significantly lower concentrations exhibited the same effectiveness due to their synergistic potency (Abdelshafi et al., 2019).

## 5 Conclusion

The cell-free extract of *A. flavus* MT321104 demonstrated the ability to biosynthesize TiO_2_ nanoparticles (TiO_2_NPs). In addition, this study successfully developed and characterized ciprofloxacin-loaded chitosan-TiO_2_ nanocomposites with superior entrapment efficiency 86.7 ± 1.8% and loading capacity 5.9 ± 0.3%. *In vitro* release demonstrated pronounced pH-responsiveness behavior enabling infection site-targeted delivery, with non-Fickian transport kinetics. UV-triggered photocatalytic enhancement provides stimuli-responsive control. Both TiO_2_NPs and CNCs exhibited significant antimicrobial activity against multidrug-resistant *S. typhimurium* and *A. fumigatus* as well as notable cytotoxic effects against human colon carcinoma and hepatocellular carcinoma cells. This study therefore highlights the potential of TiO_2_NPs synthesized within chitosan-based nanocapsules as an efficient drug delivery system capable of addressing the health risks associated with antimicrobial resistance and the limitations of conventional anticancer therapies. Ultimately, a natural, effective, non-toxic, and cost-efficient antimicrobial formulation was developed to meet the demands of antimicrobial preservation in the food industry.

## Data Availability

The original contributions presented in the study are included in the article/Supplementary material, further inquiries can be directed to the corresponding authors.
