# Correction: Antimicrobial, anticancer activities and molecular docking of eco-friendly chitosan nanocapsule loaded with biosynthesized titanium nanoparticles by *Aspergillus flavus)*

**DOI:** 10.3389/fmicb.2026.1980880

**Published:** 2026-10-29

**Authors:** Nashwa El-Gazzar, Marwa Rafat, Gamal Rabie, Basel Sitohy

**Affiliations:** 1 Department of Botany and Microbiology, Faculty of Science, Zagazig University, Zagazig, Egypt; 2 Department of Clinical Microbiology, Infection and Immunology, Umeå University, Umeå, Sweden; 3 Department of Diagnostics and Intervention, Oncology, Umeå University, Umeå, Sweden

**Keywords:** anticancer activity, antimicrobial activity, *Aspergillus flavus*, chitosan nanocapsules, encapsulation efficiency, molecular docking, titanium nanoparticles

In the **Abstract**, page 1, the MIC value is corrected to state that the MIC value is 10 μg/mL only for *S. typhimurium* and *Aspergillus fumigatus* (removing the inadvertent mention of 20 μg/mL and, respectively), matching the **Results** section.

The incorrect sentence stated:

“The minimum inhibitory concentrations (MICs) of CNCs against *Salmonella typhimurium* and *Aspergillus fumigatus* were remarkably low, at 20 and 10 μg mL^−1^, respectively.

The corrected sentence now reads:

“The minimum inhibitory concentrations (MICs) of CNCs against *Salmonella typhimurium* and *Aspergillus fumigatus* were remarkably low at 10 μg mL^−1^.”

In the **Materials and methods** section, *2.8.4 Quantification of oxidative stress*, page 6 the cell line reference to HepG-2 was incorrect.

The incorrect sentences stated:

“The most affected cell line (HepG-2) was selected for more testing to evaluate the impact of oxidative stress on cancer cells. HepG-2 cells were cultured in 24-well tissue culture plates at a density of 5 × 10^5^ cells/mL.”

The corrected sentences now read:

“The most affected cell line (HCT-116) was selected for more testing to evaluate the impact of oxidative stress on cancer cells. HCT-116 cells were cultured in 24-well tissue culture plates at a density of 5 × 10^5^ cells/mL.”

In the **Discussion** section, page 23, paragraph four there was a typographical error.

The incorrect sentence stated:

“The maximum EE% of 78.4 ± 2.3% was achieved at 1.5% w/v chitosan concentration.”

The corrected sentence now reads:

“The maximum EE% of 86.7 ±1.8% was achieved at 1.5% w/v chitosan concentration.”

In the **Conclusion** section, page 31 there was a typographical error in the accession number presented.

The incorrect sentence stated:

“The cell-free extract of *A. flavus* KF946095 demonstrated the ability to biosynthesize TiO2 nanoparticles (TiO2NPs).”

The corrected sentence states:

“The cell-free extract of *A. flavus* MT321104 demonstrated the ability to biosynthesize TiO2 nanoparticles (TiO2NPs).”

Various pieces of data were incorrect in **Results**, *3.10.1 Antifungal mechanism: CYP51B inhibition in Aspergillus fumigatus*, page 23, Table 3.

**Table 3:** Effect of CNCs on oxidative enzymes by ELISA technique.

**Sample code oxidative Sample code oxidative enzymes (nMol/mg protein)**	**CNCs**	**HCT-116 cell control (non-treated)**
Catalase	6.21 ± 0.02	11.99 ± 0.33
Superoxide dismutase (SOD)	91.62 ± 2.31	54.33 ± 3.37
Glutathione (GSH)	5.19 ± 0.84	2.45 ± 0.27
Malonaldehyde (MDA)	3.31 ± 0.23	0.88 ± 0.05

- Catalase Assay: Sample 2 updated to 6.21 ± 0.02; HCT-116 Control updated to 11.99 ± 0.33
- GSH Assay: Sample 2 updated to 5.19 ± 0.84; HCT-116 Control updated to 2.45 ± 0.27
- MDA Assay: Sample 2 updated to 3.31 ± 0.23; HCT-116 Control updated to 0.88 ± 0.05

The corrected Table 3 is below.

There were minor unintentional typographical errors and mathematical inconsistencies in Supplementary Tables 1, 2 in the tabular presentation of the inhibition zone values that were incorrectly recorded in cm. We have corrected the typographical error and expressed the inhibition-zone diameters in mm with mean and SD. Specifically, small inhibition zones representing resistance were updated to explicit resistance indicators (R) to strictly align with standard breakpoint criteria (CLSI guidelines) and to harmonize the calculated MAR index across some tested strains that we would like to correct to ensure the accuracy and consistency of the published record.

In addition, we identified typographical error for (ATCC) number for three isolates in Supplementary Table 1. The corrected values are consistent with those reported in the main text.

To maintain complete consistency between the tables and the narrative text, minor numerical adjustments were also made in the **Methods and Results** section, *2.3 Antifungal susceptibility test*, page 3.

We identified an inconsistency between the number of antifungal agents stated in the Methods section and those presented in the Supplementary Table 2.

The incorrect sentence stated:

“Nine antifungal drugs were tested, including terbinafine (TER: 30 mg), amphotericin (AMB: 20 mg), nystatin (NYS: 100 I.U.), fluconazole (FLZ: 25 mg), ketoconazole (KET: 100 mg), flucytosine (FUS: 1 mg), itraconazole (ITR: 10 mg), micafungin (MFG: 1 mg), and caspofungin (CAS: 5 mg).”

The corrected sentence now reads:

“Eight antifungal drugs were tested, including terbinafine (TER: 30 mg), amphotericin (AMB: 20 mg), nystatin (NYS: 100 I.U.), fluconazole (FLZ: 25 mg), flucytosine (FUS: 1 mg), itraconazole (ITR: 10 mg), micafungin (MFG: 1 mg), and caspofungin (CAS: 5 mg).”

In the **Results** section, *3.1 Antibacterial susceptibility of bacterial strains*, page 8 the below typographical errors were corrected.

The incorrect sentence stated:

“The MAR index of the tested indicator bacteria was as follows: 75% for S. typhimurium; 62.5% for *Pseudomonas aeruginosa* LMG 8029, *S. aureus*, and *Listeria monocytogenes*; 50% for *K. pneumoniae* ATCC 43816; 37.5% for *E. coli* and *L. innocua*; and 25% for *S. pyogenes, B. subtilis*, and *E. cloacae* (Supplementary Table 1).”

The corrected sentence now reads:

“The MAR index of the tested indicator bacteria was as follows: 75% for *S. typhimurium*; 62.5% for *Pseudomonas aeruginosa* LMG 8029, *S. aureus*, and *Listeria monocytogenes*; 50% for *K. pneumoniae* ATCC 13883; 37.5% for *E. coli* and *L. innocua*; and 25% for *S. pyogenes, B. subtilis*, and *E. cloacae* (Supplementary Table 1).”

In Supplementary Table 1 the typographical error and mistake in the MAR index (presented as percentage %) should be corrected as a decimal point.

The MAR index of the tested indicator bacteria should be corrected to 0.75 for *Salmonella typhimurium*; 0.625 for *Staphylococcus aureus, Klebsiella pneumoniae* ATCC 13883 and *Enterobacter cloacae*; 0.50 for *Listeria monocytogenes, Escherichia coli* and *Pseudomonas. aeruginosa*; 0.375 for *Streptococcus pyogenes* and *Listeria. innocua* and 0.25 for *Bacillus. Subtilis*.

The corrected Supplementary Table 1 has been published.

In the **Results** section, *3.2 Antifungal susceptibility of fungal strains*, page 8 there was an inconsistency in the abbreviation of one antifungal agent. The abbreviation should be corrected to be consistent with the terminology used in the **Methods** section. The typographical error of (MIC) should be corrected into (MFG) and (FUZ) should be corrected into (FLZ).

The incorrect sentences stated:

“Notably, resistance to FUS was observed in 100% of isolates, followed by MIC and ITR at 80% each. Conversely, 80% of the isolates exhibited high susceptibility to FUZ.”

The corrected sentences read:

“Notably, resistance to FUS was observed in 100% of isolates, followed by MFG and ITR at 80% each. Conversely, 80% of the isolates exhibited high susceptibility to FLZ.”

In the **Results** section, *3.5.6.4 Sequential pH release: gastrointestinal transit simulation*, page 19 the title of **Table 1** contains a typographical error.

The incorrect title stated:

“Antimicrobial activity of TiO_2_NPs, CsNPs, and CNCs compared to gentamycin and ketoconazole as positive controls.”

The corrected title reads:

“Antimicrobial activity of TiO_2_NPs, CsNPs, and CNCs compared to gentamycin and Fluconazole as positive controls.”

The original version of this article has been updated.

