# Supplementary material for: Antimicrobial, anticancer activities and molecular docking of eco-friendly chitosan nanocapsule loaded with biosynthesized titanium nanoparticles by Aspergillus flavus
Source: Front Microbiol. 2026 Feb 11;17:1741753. doi: 10.3389/fmicb.2026.1741753 (PMC12932164; doi:10.3389/fmicb.2026.1741753)
Supplement: Supplementary file 1 [file Data_Sheet_1.docx]

| **^Bacterial strains^** | | **^Antibacterial resistance^** | | | | | | | | |
| --- | --- | --- | --- | --- | --- | --- | --- | --- | --- | --- |
|  |  | **^Patterns^** | | | | | | | | **^MAR index^** |
|  |  | **^DA^** | **^AM^** | **^TE^** | **^GEN^** | **^OFX^** | **^AMC^** | **^P^** | **^CIP^** |  |
| **^Gram^**  **^positive^** | ***S. aureus*  DSM 1104** | **^0^** | **^1.9^** | **^2.7^** | **^2.3^** | **^0^** | **^0^** | **^0^** | **^0^** | **^5 (62.5%)^** |
|  | ***St. pyogenes* ATCC 19615** | **^0.9^** | **^0^** | **^3^** | **^2.1^** | **^0^** | **^1.2^** | **^1.7^** | **^0.9^** | **^2 (25%)^** |
|  | ***Liseria monocytogenus* LMG10470** | **^0^** | **^0^** | **^1.8^** | **^3^** | **^0^** | **^1^** | **^0^** | **^0^** | **^5 (62.5%)^** |
|  | ***L.innocua*** | **^3^** | **^0^** | **^1.2^** | **^2.1^** | **^0^** | **^0.9^** | **^0^** | **^0.9^** | **^3( 37.5%)^** |
|  | ***B.sabtulis*** | **^3^** | **^1.2^** | **^0.9^** | **^1.2^** | **^0^** | **^1.9^** | **^0^** | **^1.4^** | **^2 (25% )^** |
| **^Gram negative^** | ***E.coli* LMG 8223** | **^0.9^** | **^0^** | **^1^** | **^2.1^** | **^0^** | **^1.6^** | **^0^** | **^0.9^** | **^3 (37.5%)^** |
|  | ***Pseudomonas aeruginosa* LMG 8029** | **^0^** | **^0^** | **^0.8^** | **^1.2^** | **^0^** | **^0^** | **^0^** | **^1.7^** | **^5 (62.5%)^** |
|  | ***K. pneumonia* ATCC 43816** | **^0^** | **^2.9^** | **^0^** | **^0^** | **^0^** | **^1.1^** | **^1.7^** | **^1.1^** | **^4 (50%)^** |
|  | ***E. cloacae*** | **^1.2^** | **^2.1^** | **^0^** | **^0.9^** | **^0^** | **^0.9^** | **^1.1^** | **^0.9^** | **^2( 25%)^** |
|  | ***S.typhimurium*** | **^0^** | **^0^** | **^0^** | **^3.2^** | **^0^** | **^0^** | **^0^** | **^3^** | **^6(75%)^** |
|  | **^Isolates resistance^**  **^(%)^** | **^5(10)^**  **^50%^** | **^6(10)^**  **^60%^** | **^3(10)^**  **^30%^** | **^1(10)^**  **^10%^** | **^10(10)^**  **^100%^** | **^3(10)^**  **^30%^** | **^7(10)^**  **^70%^** | **^2(10)^**  **^20%^** |  |

**Supplementary Table 1. Antibiotic sensitivity test of the studied bacteria based on the diameter of inhibition zone (mm)**

Multiple antibiotic resistance index was calculated by using the following formula: MAR Index = Number of antibiotics to which the isolate was resistant/Total number of antibiotics tested. Clindamycin (DA: 2 µg), Ampicillin (AM: 10 µg), Tetracycline (TE: 30 µg), Ciprofloxacin (CIP: 30 µg), Ofloxacin (OFX: 5 µg), Amoxicillin (AMC: 30µg), Penicillin G (P: 10 µg), Gentamycin (GEN: 20 µg)

**Supplementary Table 2. Antifungal susceptibility results of fungal isolates**

| **^Fungal strains^** | | **^Antifungal resistance^** | | | | | | | | |
| --- | --- | --- | --- | --- | --- | --- | --- | --- | --- | --- |
|  |  | **^Patterns^** | | | | | | | | **^MAR index^** |
|  |  | **^FUZ^** | **^ITR^** | **^AMP^** | **^NYS^** | **^FUS^** | **^CAS^** | **^MIC^** | **^TER^** |  |
|  | ***P. aurantiogriseum*** | **^0^** | **^0^** | **^1.9^** | **^2^** | **^0^** | **^0^** | **^0.9^** | **^0.8^** | **^4 (50%)^** |
|  | ***C. albicans*** | **^0.9^** | **^0^** | **^0^** | **^0.9^** | **^0^** | **^1.2^** | **^0^** | **^0^** | **^5 (62.5%)^** |
|  | ***A.nivieus*** | **^1.6^** | **^1.3^** | **^2.1^** | **^1.3^** | **^0^** | **^1^** | **^0^** | **^0.9^** | **^2 (25%)^** |
|  | 1. ***flavus*** | **^1.2^** | **^0^** | **^1.6^** | **^0^** | **^0^** | **^0^** | **^0^** | **^1.2^** | **^5 (62.5%)^** |
|  | 1. ***fumigatus*** | **^1.2^** | **^0^** | **^0^** | **^0^** | **^0^** | **^0^** | **^0^** | **^0^** | **^7(87.5%)^** |
|  | **^Isolates resistance^**  **^(%)^** | **^1(5)^**  **^20%^** | **^4(5)^**  **^80%^** | **^2(5)^**  **^40%^** | **^2(5)^**  **^40%^** | **^5(5)^**  **^100%^** | **^3(5)^**  **^60%^** | **^4(5)^**  **^80%^** | **^2(5)^**  **^40%^** |  |

^1^ AMP: amphotericin B, NYS: nystatin, FUZ: fluconazole, ITR: itraconazole, FUS: flucytosine, CAS: caspofungin, MFG: micafungin, TER: terbinafine

**Supplementary Table3.** Polydispersity index analysis for all nanoparticle systems using dynamic light scattering (DLS) intensity autocorrelation function analysis via the cumulants method**.**

| Sample | PDI Value | Classification | Size Distribution |
| --- | --- | --- | --- |
| Chitosan NPs | 0.21 ± 0.02 | Highly Monodisperse | 40.6 ± 5.8 nm |
| TiO₂ NPs | 0.24 ± 0.03 | Monodisperse | 40.7 ± 6.2 nm |
| CNCs | 0.28 ± 0.03 | Monodisperse | 87.3 ± 12.5 nm |

**Supplementary Table4.** Temporal Evolution of Hydrodynamic Diameter

| Time (Days) | ChNPs Size (nm) | TiO₂ Size (nm) | CNCs Size (nm) |
| --- | --- | --- | --- |
| 0 | 40.6 ± 1.2 | 40.7 ± 1.3 | 87.3 ± 2.1 |
| 7 | 41.2 ± 1.3 | 41.5 ± 1.4 | 88.5 ± 2.3 |
| 14 | 41.8 ± 1.4 | 42.3 ± 1.5 | 89.8 ± 2.5 |
| 21 | 42.3 ± 1.5 | 43.1 ± 1.6 | 91.2 ± 2.7 |
| 30 | 42.7 ± 1.6 | 43.8 ± 1.7 | 92.5 ± 2.9 |

**Supplementary Table5.** Temporal Evolution of Zeta Potential

| Time (Days) | ChNPs ζ (mV) | TiO₂ ζ (mV) | CNCs ζ (mV) |
| --- | --- | --- | --- |
| 0 | +31.2 ± 3.8 | -30.1 ± 4.5 | +20.8 ± 4.3 |
| 7 | +30.6 ± 3.9 | -29.5 ± 4.6 | +20.3 ± 4.4 |
| 14 | +30.1 ± 4.0 | -28.9 ± 4.7 | +19.9 ± 4.5 |
| 21 | +29.5 ± 4.1 | -28.3 ± 4.8 | +19.5 ± 4.6 |
| 30 | +29.1 ± 4.2 | -27.8 ± 4.9 | +19.2 ± 4.7 |

## Characterization Values

| Sample | Mean Size (nm) | PDI | Zeta Potential (mV) | Colloidal Stability |
| --- | --- | --- | --- | --- |
| Chitosan NPs | 40.6 ± 5.8 | 0.21 ± 0.02 | +31.2 ± 3.8 | Excellent (>±30 mV) |
| TiO₂ NPs | 40.7 ± 6.2 | 0.24 ± 0.03 | -30.1 ± 4.5 | Excellent (>±30 mV) |
| CNCs | 87.3 ± 12.5 | 0.28 ± 0.03 | +20.8 ± 4.3 | Good (>±20 mV) |

**Supplementary Table 6. Optimized Formulation: Comprehensive Characterization** **via multiple**

**analytical techniques**

| Parameter | Value | Characterization Method |
| --- | --- | --- |
| Entrapment Efficiency (%) | 86.7 ± 1.8 | UV spectrophotometry (λ=278 nm) |
| Loading Capacity (%) | 5.9 ± 0.3 | Gravimetric + UV quantification |
| Mean Hydrodynamic Diameter (nm) | 45.8 ± 1.2 | Dynamic Light Scattering (DLS) |
| Polydispersity Index (PDI) | 0.24 ± 0.03 | Cumulants analysis (DLS) |
| Zeta Potential (mV) | +25.8 ± 4.1 | Electrophoretic light scattering |
| TEM Core Diameter (nm) | 38.2 ± 6.5 | Transmission electron microscopy |
| Drug Loading (mg/g) | 59 ± 3 | HPLC quantification |
| pH | 6.2 ± 0.3 | pH meter (aqueous suspension) |
| Colloidal Stability | Excellent (\|ζ\| > 20 mV) | DLVO theory classification |
| Storage Stability (30 days) | <8% size change | DLS time-course monitoring |

**
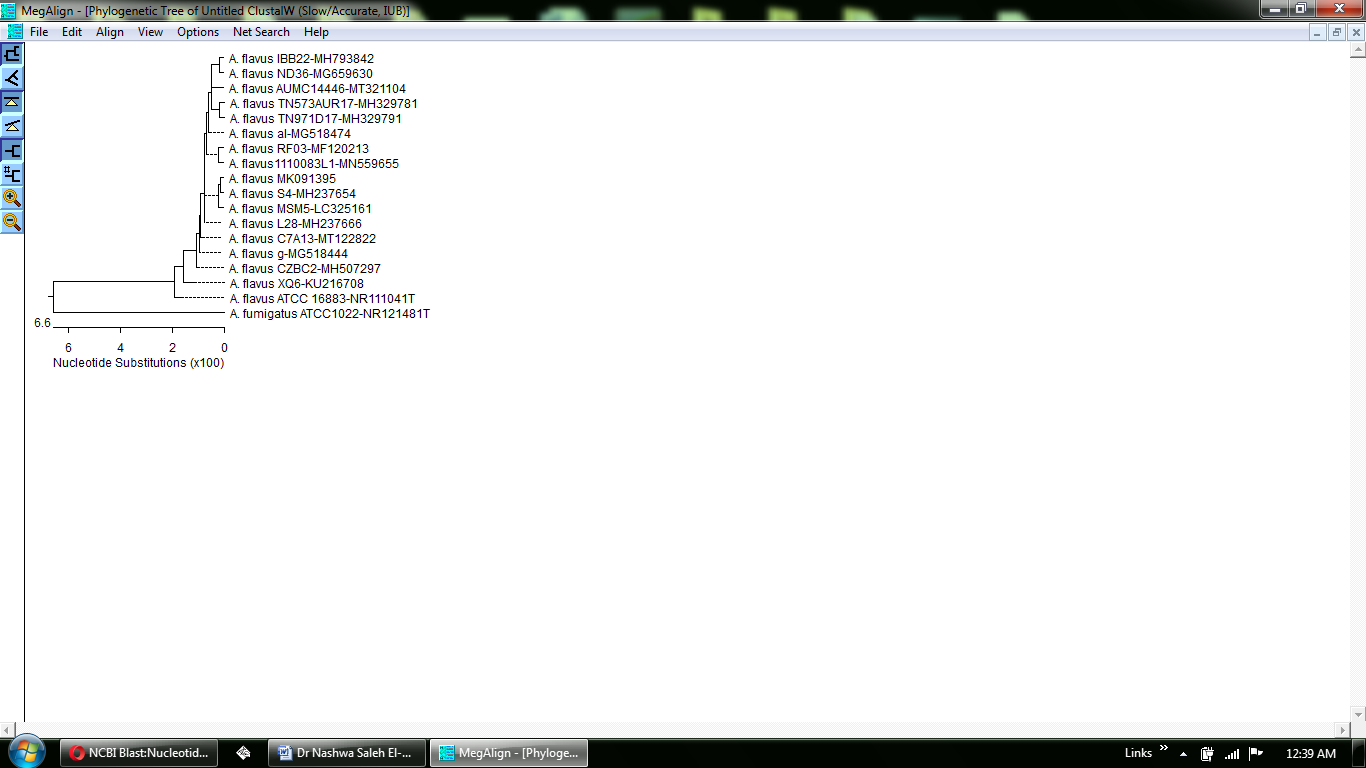
**

**Supplementary Figure 1.** Phylogenetic tree based on ITS sequences of rDNA of *A. flavus* AUMC14446 (arrowed) isolated in the present aligned with closely related sequences accessed from the GenBank. (*A.* = *Aspergillus*, *A. fumigatus* is included as an outgroup strain. **Note:** ITS sequences of *A. flavus* AUMC14446 (Genank accession No.MT321104) showed 99.81% identity and 100% coverage with several strains of *A. flavus* and 99.06% and 100% covaerage with the type strain of *A. flavus* (ATCC16883, gb:NR111041).

*
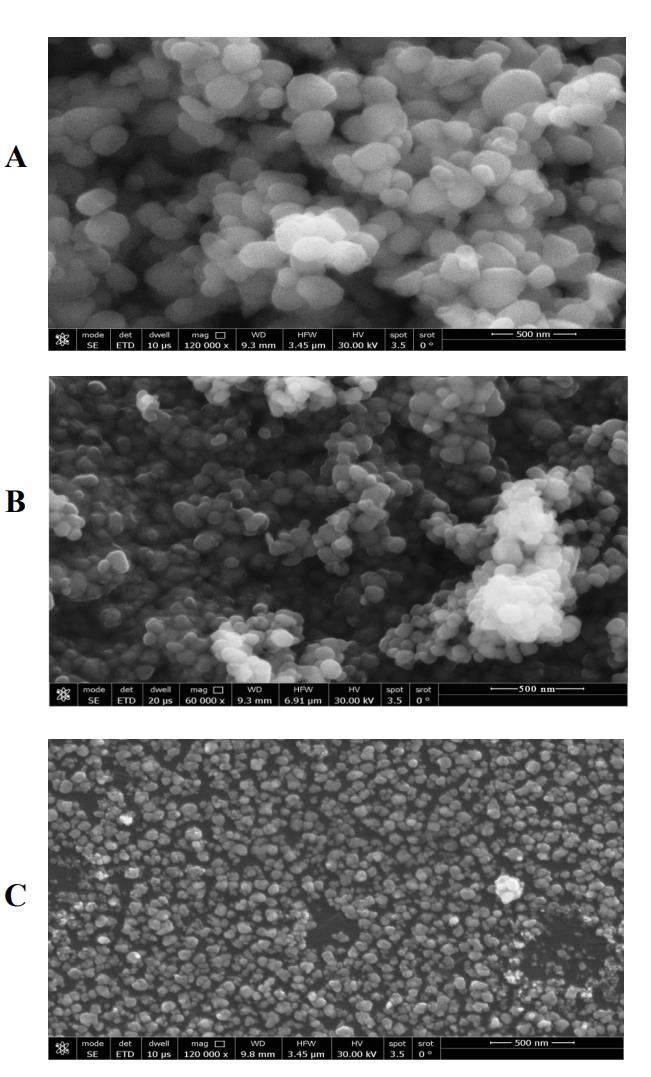
*

**Supplementary Figure 2.** SEM characterization for nanoparticles. **(A**) TiO_2_NPs, (**B**) CsNPs and (**C**) CNCs nanocomposite**.**

*
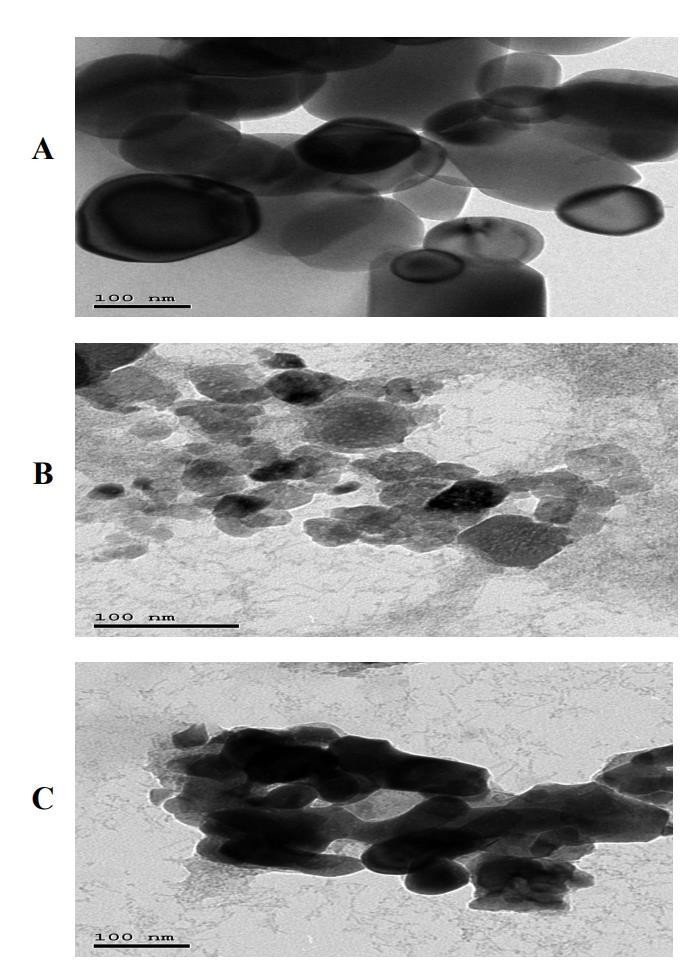
*

**Supplementary Figure 3.** TEM characterization for nanoparticles. **(A**) TiO_2_NPs, (**B**) CsNPs and (**C**) CNCs nanocomposite**.**

**
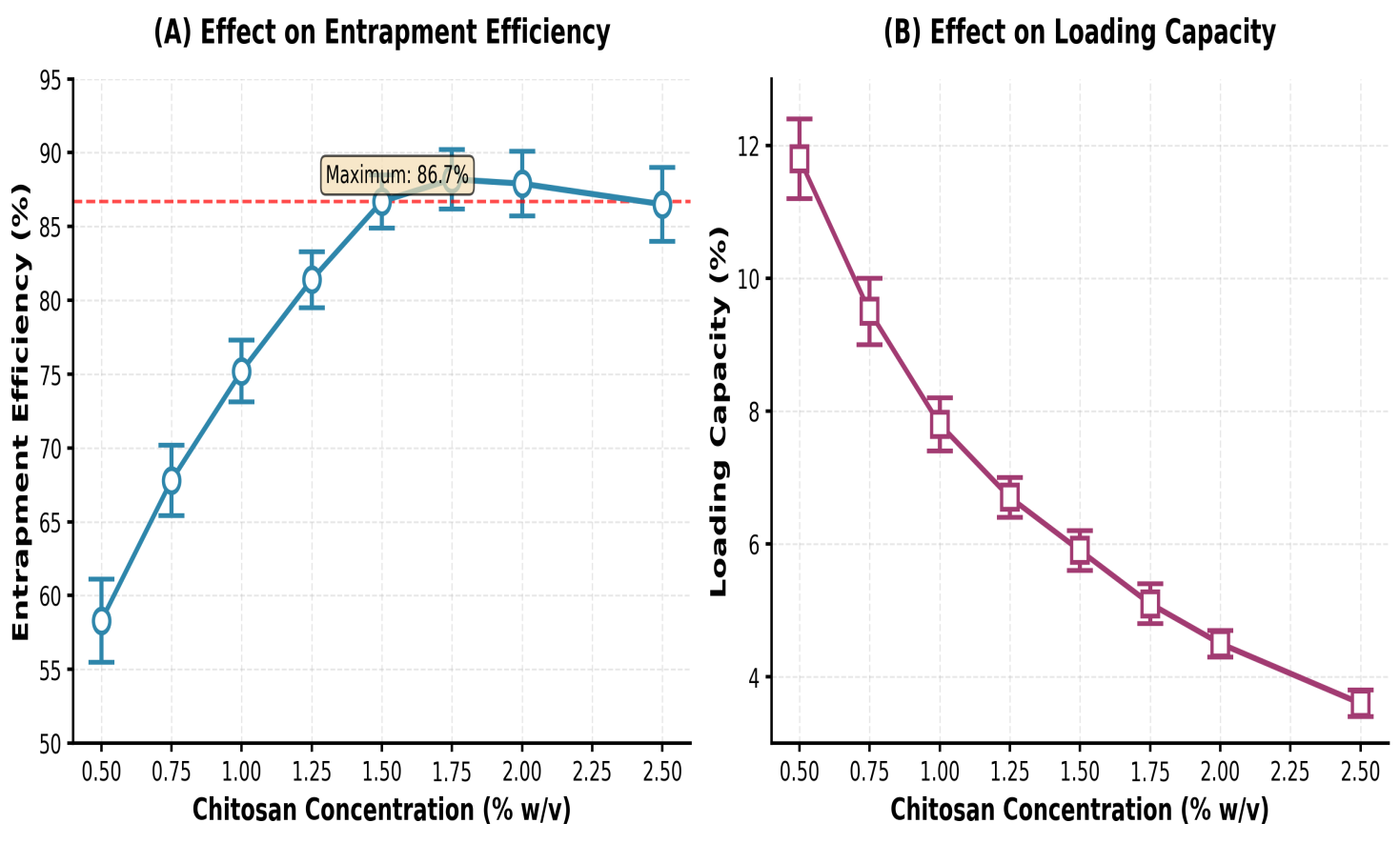
**

**Supplementary Figure 4.** Effect of chitosan concentration on drug loading performance. (A) Entrapment efficiency and (B) Loading Capacity

**
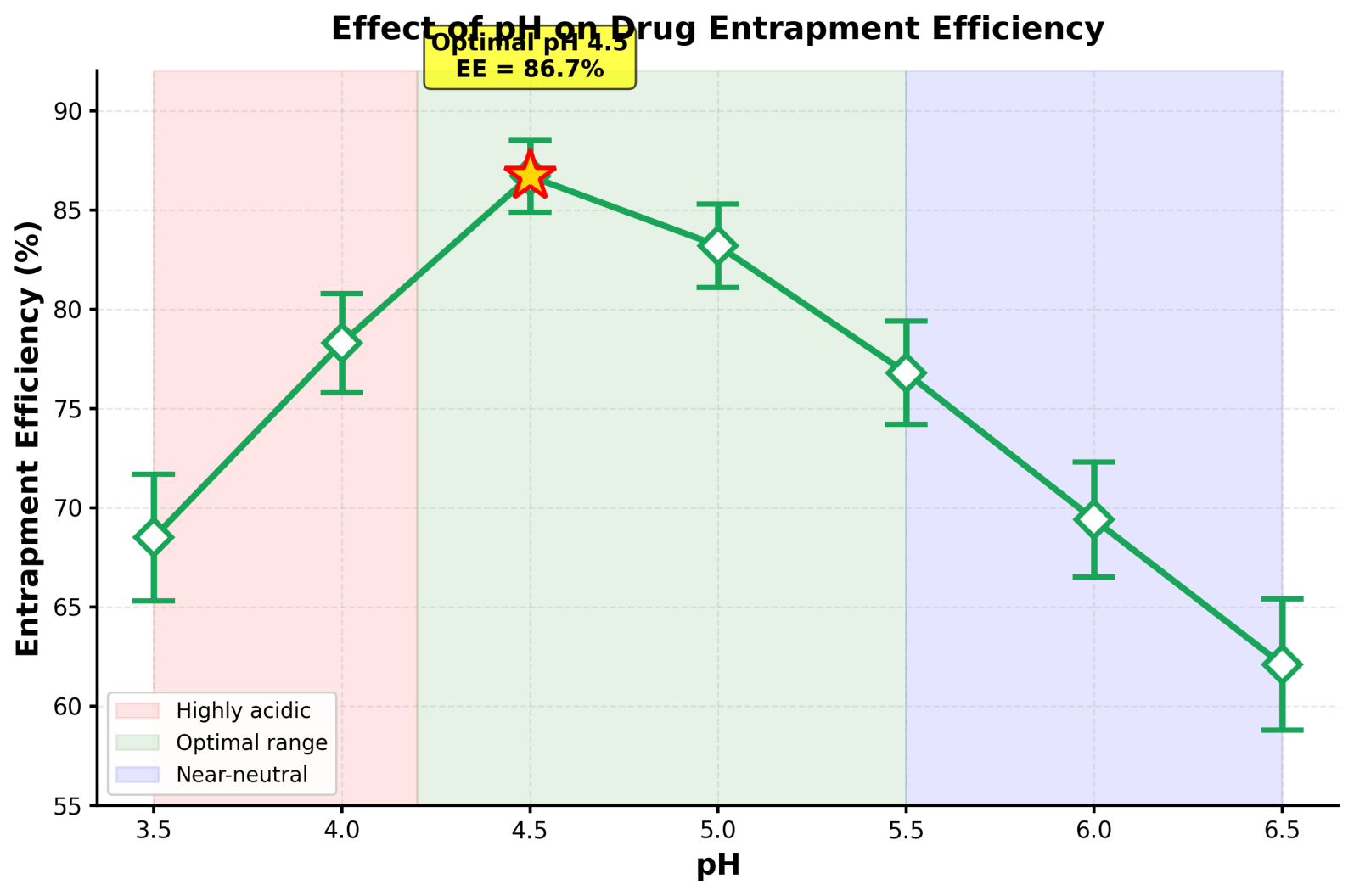
**

**Supplementary Figure 5.** Effect of pH on entrapment efficiency

**
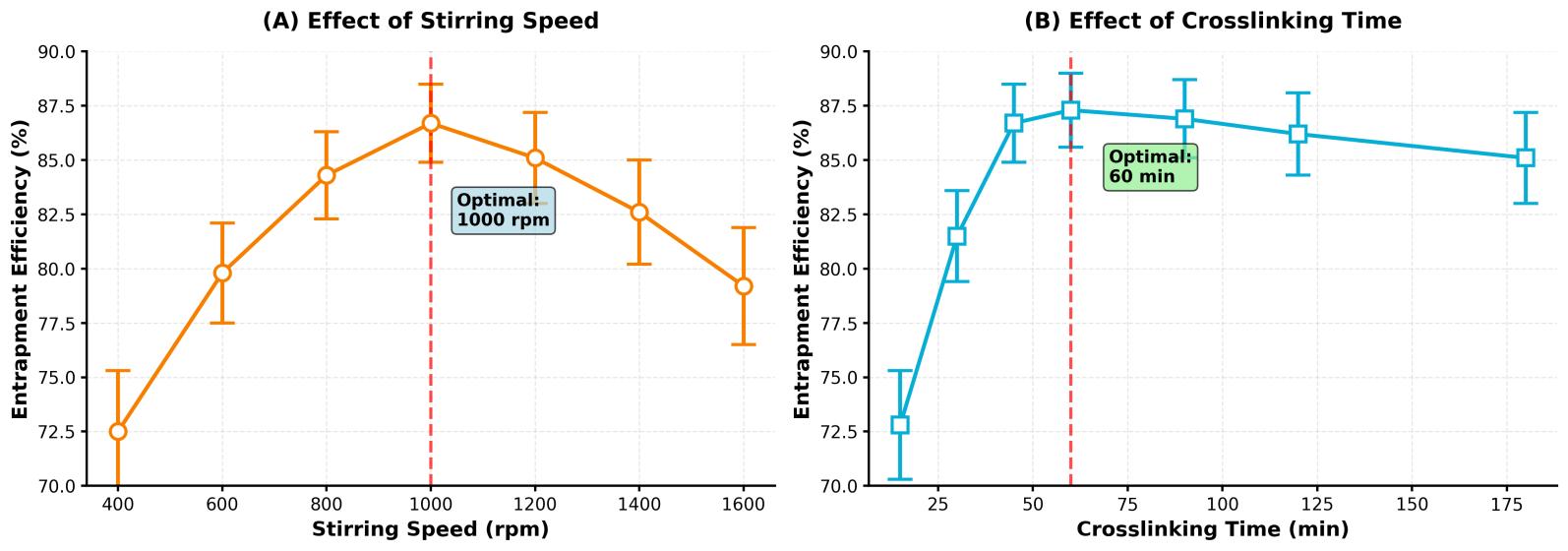
**

**Supplementary Figure 6.** Effect of process Parameter on entrapment efficiency. (A) Stirring Speed and (B) Crosslinking Time

**
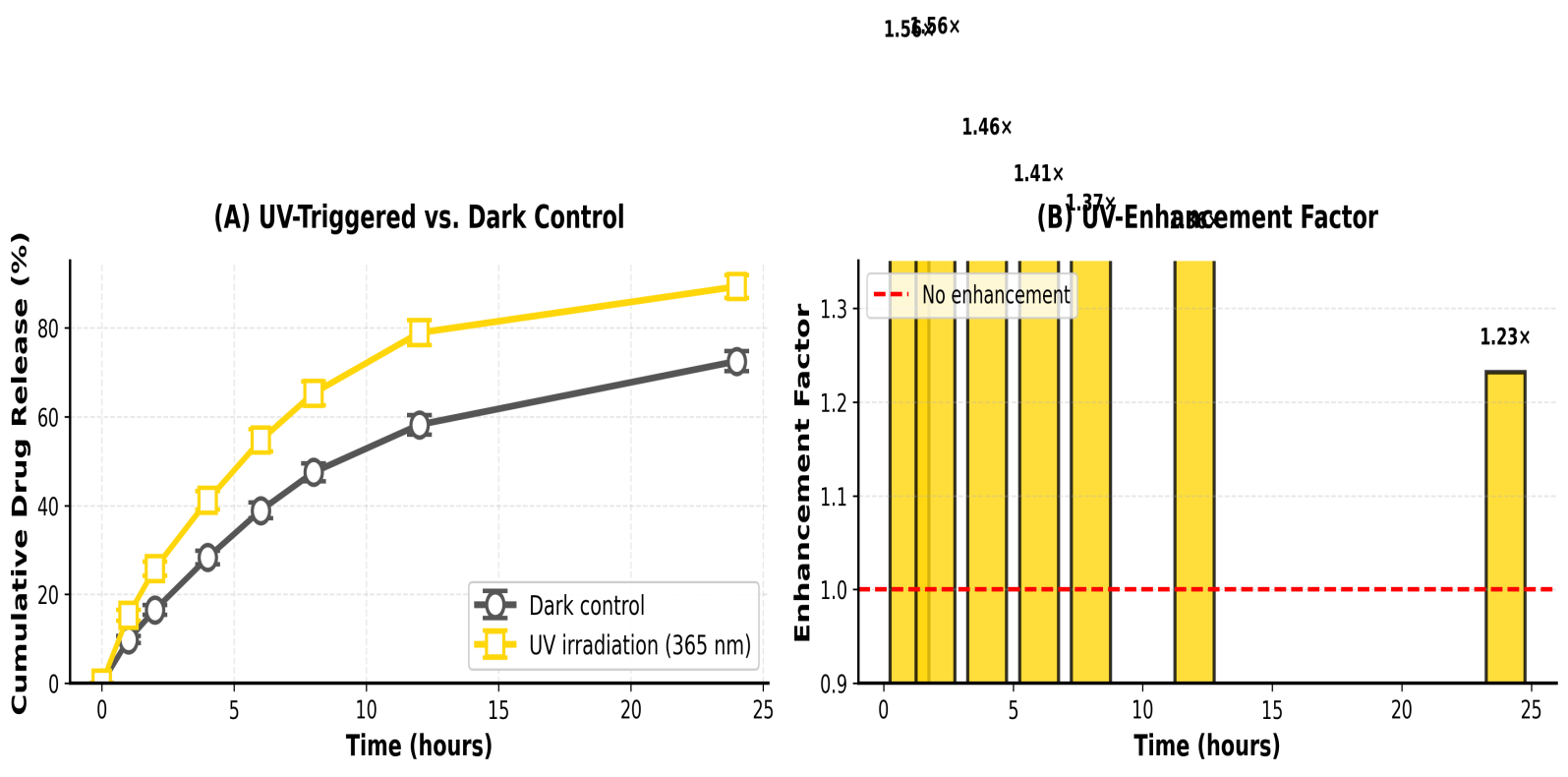
**

**Supplementary Figure 7.** Drug release enhancement (A) UV-Light Triggered and (B) Photo catalytic Release


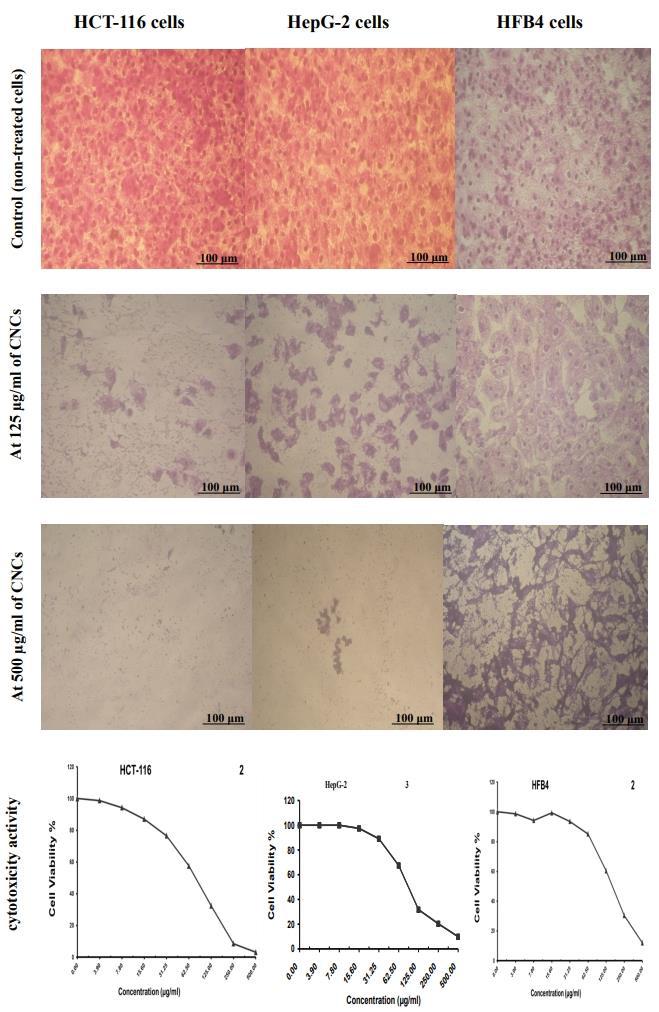


**Supplementary Figure 8.** Cytotoxicity and Inhibitory activities of different concentration of CNCs against HCT-116, HepG-2 cells and normal HFB4 cells. Inhibitory activity of CNCs against HCT-116 cells with IC_50 =_ 81.20 ± 4.22 µg/mL, Inhibitory activity of CNCs against hepatocellular carcinoma cells (HepG-2) with IC_50 =_ *93.1 ± 3.4* µg/mL and cytotoxicity activity of CNCs against normal HFB4 cells with CC_50_ values were 200.54 ± 7.06 µg/ mL.


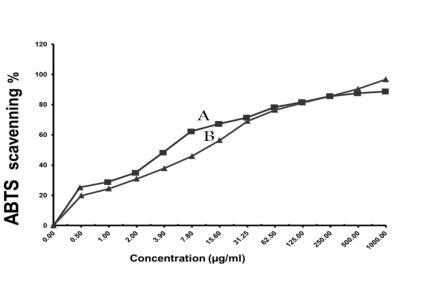


**Supplementary Figure 9.** ABTS Scavenging activity (%) of CNCs with IC_50_. ( **A**) CNCs and (**B**) Ascorbic acid standard.


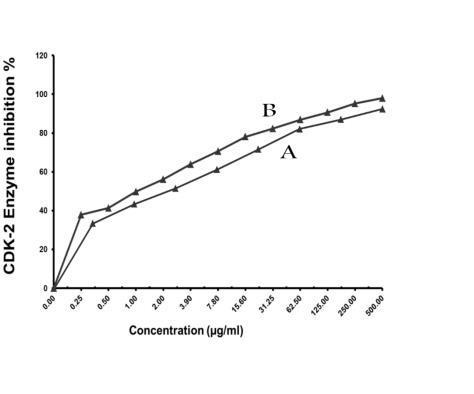


**Supplementary Figure 10.** CDK-2 Enzyme inhibition assay of CNCs. (**A)** CNCs and **(B)** Roscovitine; Reference Standard
