# Supplementary material for: Antimicrobial, anticancer activities and molecular docking of eco-friendly chitosan nanocapsule loaded with biosynthesized titanium nanoparticles by Aspergillus flavus
Source: Front Microbiol. 2026 Feb 11;17:1741753. doi: 10.3389/fmicb.2026.1741753 (PMC12932164; doi:10.3389/fmicb.2026.1741753)
Supplement: Supplementary file 2 [file Supplementary_file_1.docx]

**Supplementary Table 1. Antibiotic sensitivity test of the bacterial isolates based on inhibition zone diameter (mm)**

| ^   Bacterial strains^ |  | ^**Antibacterial resistance**^ |  |  |  |  |  |  |  |  |
| --- | --- | --- | --- | --- | --- | --- | --- | --- | --- | --- |
|  |  | ^**Patterns**^ |  |  |  |  |  |  |  | ^**MAR index**^ |
|  |  | ^**DA**^ | ^**AM**^ | ^**TE**^ | ^**GEN**^ | ^**OFX**^ | ^**AMC**^ | ^**P**^ | ^**CIP**^ |  |
| ^Gram^ ^positive^ | ***Staphylococcus*** ***aureus***  **ATCC 25923** | **R** | **20.03 ±** **0.89** | **27.93 ±** **0.90** | **18.17± 0.15** | **R** | **R** | **R** | **R** | ^**0.625**^ |
|  | ***Streptococcus pyogenes*** **ATCC 19615** | **R** | **R** | **31.50 ±** **1.32** | **18.53 ± 0.92** | **18.50±** **0.50** | **13.43** **±** **1.36** | **R** | **18.20 ±** **1.12** | ^**0.375**^ |
|  | ***Listeria innocua*** **LMG 11387** | **30.60** **± 0.66** | **R** | **20.50** **± 0.50** | **18.90 ± 0.78** | **R** | **21.50 ± 0.50** | **R** | **17.50** **± 0.50** | ^**0. 375**^ |
|  | ***Listeria monocytogenes*** **LMG 10470** | **R** | **R** | **19.43 ±** **1.29** | **18.53 ± 0.50** | **R** | **19.50 ± 0.50** | **R** | **18.40 ± 0.69** | ^**0.50**^ |
|  | ***Bacillus subtilis***  **RCMB 015 )NRRL 543(** | **30.63** **± 0.71** | **12.67** **± 0.70** | **16.50** **± 0.50** | **19.93 ± 0.81** | **R** | **19.63** **± 0.65** | **R** | **15.10** **± 1.42** | ^**0.25**^ |
| ^Gram negative^ | ***Escherichia coli*** **ATCC 25922** | **R** | **17.50** **± 0.50** | **R** | **19.30 ± 0.75** | **R** | **16.40** **± 0.40** | **R** | **18.50** **± 0.50** | ^**0.50**^ |
|  | ***Pseudomonas aeruginosa***  **LMG 8029** | **R** | **R** | **18.50** **± 0.50** | **18.97 ± 0.45** | **21.50 ± 0.50** | **R** | **R** | **19.10** **± 1.85** | ^**0. 50**^ |
|  | ***Klebsiella  pneumoniae*** **ATCC 13883** | **R** | **30.10** **± 1.28** | **R** | **18.67 ± 0.76** | **R** | **R** | **18.13** **± 0.99** | **R** | ^**0.625**^ |
|  | ***Enterobacter cloacae*** **ATCC 23355** | **12.67** **± 0.65** | **21.60** **± 0.66** | **R** | **18.27 ± 0.25** | **R** | **R** | **R** | **R** | ^**0.625**^ |
|  | ***Salmonella*** ***typhimurium*** **ATCC 14028** | **R** | **R** | **R** | **19.47 ± 0.50** | **R** | **R** | **R** | **30.46** **± 0.42** | ^**0.75**^ |
|  | ^**Isolates resistance**^ ^(%)^ | ^**7(10)**^ ^**70%**^ | ^**5(10)**^ ^**50%**^ | ^**4(10)**^ ^**40%**^ | ^**0(10)**^ ^**0%**^ | ^**8(10)**^ ^**80%**^ | ^**5(10)**^ ^**50%**^ | ^**9(10)**^ ^**90%**^ | ^**3(10)**^ ^**30%**^ |  |

Numeric values represent inhibition zone diameters (mm), while 'R' indicates resistant strains according to CLSI guideline. Data represented as mean ± standard deviation (SD). Clindamycin (DA: 2 µg), Ampicillin (AM: 10 µg), Tetracycline (TE: 30 µg), Gentamicin (GEN: 10 µg), Ofloxacin (OFX: 5 µg), Amoxicillin (AMC: 30 µg), Penicillin G (P: 10 µg), and Ciprofloxacin (CIP: 5 µg). R: Resistant. MAR Index = Number of antibiotics to which the isolate was resistant / Total number of antibiotics tested.

**Supplementary Table 2. Antifungal susceptibility results of fungal** **isolates** 	

| ^**Fungal  strains**^ |  | ^**Antifungal resistance**^ |  |  |  |  |  |  |  |  |
| --- | --- | --- | --- | --- | --- | --- | --- | --- | --- | --- |
|  |  | ^**Patterns**^ |  |  |  |  |  |  |  | ^**MAR index**^ |
|  |  | ^**FLZ**^ | ^**ITR**^ | ^**AMP**^ | ^**NYS**^ | ^**FUS**^ | ^**CAS**^ | ^**MFG**^ | ^**TER**^ |  |
|  | ***Penicillium aurantiogriseum*** | **17.43 ± 0.40** | **R** | **19.47** **± 0.46** | **20.37** **± 0.40** | **R** | **R** | **16.50 ±** **0.50** | **R** | ^**0.50**^ |
|  | ***Candida albicans*** | **17.87 ± 0.81** | **R** | **R** | **R** | **R** | **13.40** **± 1.35** | **R** | **R** | ^**0.75**^ |
|  | ***Aspergillus nivieus*** | **18.20 ± 0.35** | **13.23** **± 0.21** | **21.30** **± 0.26** | **13.43** **± 0.40** | **R** | **11.03** **± 1.17** | **R** | **16.60** **± 0.28** | ^**0.25**^ |
|  | ***Aspergillus flavus*** | **17.90 ± 0.17** | **R** | **16.36** **± 0.32** | **R** | **R** | **R** | **R** | **16.50 ±** **0.50** | ^**0.625**^ |
|  | ***Aspergillus fumigatus*** | **18.60 ± 0.26** | **R** | **R** | **R** | **R** | **R** | **R** | **R** | ^**0.875**^ |
|  | ^**Isolates resistance**^ ^**(%)**^ | ^**0(5)**^ ^**0%**^ | ^**4(5)**^ ^**80%**^ | ^**2(5)**^ ^**40%**^ | ^**3(5)**^ ^**60%**^ | ^**5(5)**^ ^**100%**^ | ^**3(5)**^ ^**60%**^ | ^**4(5)**^ ^**80%**^ | ^**3(5)**^ ^**60%**^ |  |

^1^

 

FLZ: fluconazole (25 µg), ITR: itraconazole (10 µg), AMP: amphotericin B (10 µg), NYS: nystatin (100 units), FUS: flucytosine (10 µg), CAS: caspofungin (5 µg), MFG: micafungin (5 µg), TER: terbinafine (30 µg). **R**: Resistant. Numeric values represent inhibition zone diameters (mm). Data represented as mean ± standard deviation (SD). MAR Index = Number of antifungals to which the isolate was resistant / Total number of antifungals tested.

**Supplementary Table3.** Polydispersity index analysis for all nanoparticle systems using dynamic light scattering (DLS) intensity autocorrelation function analysis via the cumulants method**.**

| Sample | PDI Value | Classification | Size Distribution |
| --- | --- | --- | --- |
| Chitosan NPs | 0.21 ± 0.02 | Highly Monodisperse | 40.6 ± 5.8 nm |
| TiO₂ NPs | 0.24 ± 0.03 | Monodisperse | 40.7 ± 6.2 nm |
| CNCs | 0.28 ± 0.03 | Monodisperse | 87.3 ± 12.5 nm |

**Supplementary Table4.** Temporal Evolution of Hydrodynamic Diameter

| Time (Days) | ChNPs Size (nm) | TiO₂ Size (nm) | CNCs Size (nm) |
| --- | --- | --- | --- |
| 0 | 40.6 ± 1.2 | 40.7 ± 1.3 | 87.3 ± 2.1 |
| 7 | 41.2 ± 1.3 | 41.5 ± 1.4 | 88.5 ± 2.3 |
| 14 | 41.8 ± 1.4 | 42.3 ± 1.5 | 89.8 ± 2.5 |
| 21 | 42.3 ± 1.5 | 43.1 ± 1.6 | 91.2 ± 2.7 |
| 30 | 42.7 ± 1.6 | 43.8 ± 1.7 | 92.5 ± 2.9 |

**Supplementary Table5.** Temporal Evolution of Zeta Potential

| Time (Days) | ChNPs ζ (mV) | TiO₂ ζ (mV) | CNCs ζ (mV) |
| --- | --- | --- | --- |
| 0 | +31.2 ± 3.8 | -30.1 ± 4.5 | +20.8 ± 4.3 |
| 7 | +30.6 ± 3.9 | -29.5 ± 4.6 | +20.3 ± 4.4 |
| 14 | +30.1 ± 4.0 | -28.9 ± 4.7 | +19.9 ± 4.5 |
| 21 | +29.5 ± 4.1 | -28.3 ± 4.8 | +19.5 ± 4.6 |
| 30 | +29.1 ± 4.2 | -27.8 ± 4.9 | +19.2 ± 4.7 |

## Characterization Values

| Sample | Mean Size (nm) | PDI | Zeta Potential (mV) | Colloidal Stability |
| --- | --- | --- | --- | --- |
| Chitosan NPs | 40.6 ± 5.8 | 0.21 ± 0.02 | +31.2 ± 3.8 | Excellent (>±30 mV) |
| TiO₂ NPs | 40.7 ± 6.2 | 0.24 ± 0.03 | -30.1 ± 4.5 | Excellent (>±30 mV) |
| CNCs | 87.3 ± 12.5 | 0.28 ± 0.03 | +20.8 ± 4.3 | Good (>±20 mV) |

**Supplementary Table 6. Optimized Formulation: Comprehensive Characterization** **via multiple** 

**analytical techniques** 

| Parameter | Value | Characterization Method |
| --- | --- | --- |
| Entrapment Efficiency (%) | 86.7 ± 1.8 | UV spectrophotometry (λ=278 nm) |
| Loading Capacity (%) | 5.9 ± 0.3 | Gravimetric + UV quantification |
| Mean Hydrodynamic Diameter (nm) | 45.8 ± 1.2 | Dynamic Light Scattering (DLS) |
| Polydispersity Index (PDI) | 0.24 ± 0.03 | Cumulants analysis (DLS) |
| Zeta Potential (mV) | +25.8 ± 4.1 | Electrophoretic light scattering |
| TEM Core Diameter (nm) | 38.2 ± 6.5 | Transmission electron microscopy |
| Drug Loading (mg/g) | 59 ± 3 | HPLC quantification |
| pH | 6.2 ± 0.3 | pH meter (aqueous suspension) |
| Colloidal Stability | Excellent (│ζ│ > 20 mV) | DLVO theory classification |
| Storage Stability (30 days) | <8% size change | DLS time-course monitoring |

**Supplementary Figure 1.** Phylogenetic tree based on ITS sequences of rDNA of *A. flavus* AUMC14446 (arrowed) isolated in the present aligned with closely related sequences accessed from the GenBank. (*A.* = *Aspergillus*, *A. fumigatus* is included as an outgroup strain. **Note:** ITS sequences of *A. flavus* AUMC14446 (Genank accession No.MT321104) showed 99.81% identity and 100% coverage with several strains of *A. flavus* and 99.06% and 100% covaerage with the type strain of *A. flavus* (ATCC16883,  gb:NR111041).

	**Supplementary Figure 2.** SEM characterization for nanoparticles. **(A**) TiO_2_NPs, (**B**) CsNPs and (**C**) CNCs nanocomposite**.**

**Supplementary Figure 3.** TEM characterization for nanoparticles. **(A**) TiO_2_NPs, (**B**) CsNPs and (**C**) CNCs nanocomposite**.**

**Supplementary Figure 4.** Effect of chitosan concentration on drug loading performance. (A) Entrapment efficiency and (B) Loading Capacity

**Supplementary Figure 5.** Effect of pH on entrapment efficiency

**Supplementary Figure 6.** Effect of process Parameter on entrapment efficiency. (A) Stirring Speed and (B) Crosslinking Time

**Supplementary Figure 7.** Drug release enhancement (A) UV-Light Triggered and (B) Photo catalytic Release

**Supplementary** **Figure 8.** Cytotoxicity and Inhibitory activities of different concentration of CNCs against HCT-116, HepG-2 cells and normal HFB4 cells. Inhibitory activity of CNCs against HCT-116 cells with IC_50 = _81.20 ± 4.22 µg/mL, Inhibitory activity of CNCs against hepatocellular carcinoma cells (HepG-2) with IC_50 = _*93.1 ± 3.4* µg/mL and cytotoxicity activity of CNCs against normal HFB4 cells with CC_50_ values were 200.54 ± 7.06 µg/ mL.

**Supplementary Figure 9.** ABTS Scavenging activity (%) of CNCs with IC_50_. ( **A**) CNCs and (**B**) Ascorbic acid standard. 

**Supplementary Figure 10.** CDK-2 Enzyme inhibition assay of CNCs. (**A)** CNCs and **(B)** Roscovitine; Reference Standard
